# Micellar “Click” Nanoreactors: Spiking Pluronic-Based Micelles with Polymeric Ligands

**DOI:** 10.1021/acs.macromol.4c01425

**Published:** 2024-11-04

**Authors:** Krishna Vippala, Shreyas Shankar Wagle, Parul Rathee, Keerthana Mulamukkil, Yousif Ayoub, Arthur Komlosh, Sharon Gazal, Bianca Avramovitch, Roey J. Amir

**Affiliations:** † Department of Organic Chemistry, School of Chemistry, Faculty of Exact Sciences, Tel-Aviv University, Tel- Aviv 6997801, Israel; ‡ Tel-Aviv University Center for Nanoscience and Nanotechnology, Tel-Aviv University, Tel-Aviv 6997801, Israel; § Analytical Technologies Unit R&D, Teva Pharmaceutical Industries, Kfar Saba, Teva 4410202, Israel; ∥ The Center for Physics and Chemistry of Living Systems, Tel-Aviv University, Tel-Aviv 6997801, Israel; ⊥ ADAMA Center for Novel Delivery Systems in Crop Protection, Tel-Aviv University, Tel-Aviv 6997801, Israel

## Abstract

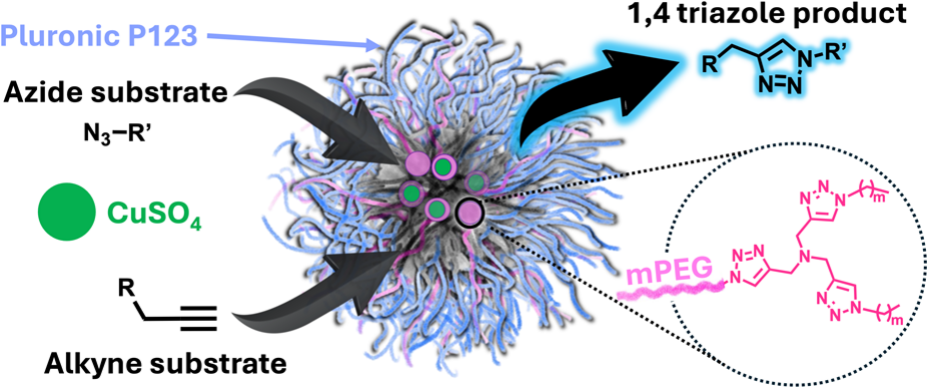

In recent years, the development of nanoreactors, such as micellar nanoreactors (MNRs) for catalytic transformations, has gained significant attention due to their potential in enhancing reaction rates, selectivity, efficiency, and, as importantly, the ability to conduct organic chemistry in aqueous solutions. Among these, the copper(I)-catalyzed azide–alkyne cycloaddition (CuAAC) reaction represents a pivotal transformation and is widely used in the synthesis of bioconjugates, pharmaceuticals, and advanced materials. This study aims toward advancing our understanding of the design and utilization of polymeric amphiphiles containing tris-triazole ligands as an integral element for CuAAC reactions within MNRs. Specifically, our investigation delves into three critical factors that influence the reaction rate within MNRs: hydrophobicity, architectural configuration of the polymeric ligands, and their concentration. Utilizing the high molecular precision of dendritic amphiphiles, we synthesized polymeric ligands with two distinct architectures, namely, PEG-ditris-triazole amphiphile (DTA) and PEG-monotris-triazole amphiphile (MTA), and explored their CuAAC reactivity through coassembly with commercially available Pluronic P123 amphiphiles. The results indicate that the architecture and the concentration of the polymeric ligands play more dominant roles in influencing the reaction rate than the hydrophobicity of the dendritic blocks. Notably, while MNRs assembled from solely DTA showed a dampened reaction rate, spiking P123 micelles with DTA yielded an MNR with significantly faster rates. Moreover, P123 MNRs spiked with the synthesized MTA demonstrated increased CuAAC reaction rates compared to those spiked with the DTA, and they even outperformed the widely used Tris(benzyltriazolylmethyl)amine ligand. These findings provide valuable insights into the design principles of polymer-based ligands for constructing reactive MNRs and other types of nanoreactors for efficient catalytic transformations.

## Introduction

The catalysis of abiotic reactions with transition metals (TMs) has garnered significant attention as a strategy to mimic the selectivity and efficiency of enzymes in nonbiological and biological systems.^1−10^ However, unlike enzymes, which are highly efficient and selective catalysts operating under mild conditions, TM-based catalysts often require harsh reaction conditions, necessitate the use of organic solvents, and are often noncompatible with aqueous media. Furthermore, the intrinsic toxicity of TMs hinders their viability for in vivo applications.^11,12^ Consequently, there has been a growing interest in developing strategies to construct nanosized scaffolds that can mimic the reactivity of enzymes in vitro and in nonbiological systems.^13−15^ For example, the Zimmerman’s group designed nanoreactors based on single-chain nanoparticles capable of performing abiotic catalysis both on the cell surface^16^ and within the cell.^17^ Among the various nanoscaffolds available, polymeric micelles, which are nanostructures composed of a hydrophobic core and a hydrophilic shell and can solubilize a wide range of hydrophobic molecules, have become an attractive option for creating enzyme-like catalysts in nonbiological systems.^18−25^ The use of micellar nanoreactors (MNRs) for the confinement of TMs can potentially minimize their toxicity while maintaining the catalytic activity of the TM, making MNRs a desirable platform for performing abiotic reactions in vitro and in vivo.^26^

Among the numerous TM-catalyzed reactions suitable for bioorthogonal transformations, the copper-catalyzed azide–alkyne cycloaddition (CuAAC) reaction is notable for its high regioselectivity, efficiency, and the ability to conduct it in cells.^27,28^ The CuAAC reaction rate can be influenced by a variety of factors, including the nature of the reactants, the solvent system, and the presence of additives.^29^ A key component of the CuAAC reaction is the ligand, which can significantly influence the reaction rate and stability of the metal catalyst.^30,31^ In recent years, various ligands have been developed to improve the efficiency and specificity of the CuAAC reaction, such as phosphine ligands,^32−34^ N-heterocyclic carbene (NHC) ligands,^35,36^ triazole-based ligands,^37,38^ and peptide-based ligands.^39^ Among these, tris-triazole-based ligands have demonstrated high efficiency in accelerating the CuAAC reaction rate.^40,41^

While the use of MNRs for conducting CuAAC has been documented^27^ and the utilization of modified tris-triazole ligands to enhance water solubility for CuAAC in aqueous surroundings has also been explored,^42,43^ deeper understanding of the structure–reactivity relations for MNR containing polymer-based tris-triazole ligands can broaden the applicability of such systems for both biomedical applications and green chemistry. Recently, we reported that increasing the hydrophobicity of the dendron in linear–dendritic amphiphiles can enhance the reaction rate of Pd-mediated depropargylation inside MNRs.^22,43^ Herein, we aimed to investigate if the same trend holds true for the MNRs that can catalyze the CuAAC reaction. With this objective in mind, we designed and synthesized PEG-dendron amphiphiles with tris-triazoles serving both as ligands and as dendritic branching motifs. These dendritic amphiphiles were functionalized with end-groups of increasing length and hydrophobicity, and the reactivity of their self-assembled MNRs for the CuAAC reaction in aqueous media was studied.

## Results and Discussion

### Design and Synthesis of Ditris-Triazole-Based Amphiphiles (DTAs)

The molecular design of the amphiphiles was based on utilizing tris-triazole to serve both as the ligand for copper ions as well as the branching units of the dendrons. The amphiphiles were composed of a 5 kDa methoxy polyethylene glycol (mPEG) as the hydrophilic block and a second-generation dendron with two tris-triazole ligands and a total of four linear alkyl chains of three different lengths as the hydrophobic block.

**Scheme 1:**
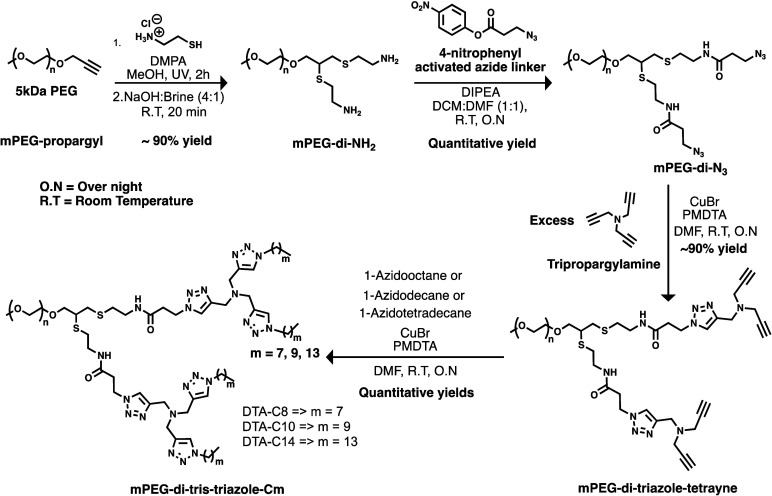
Synthetic Route for DTAs

The synthesis of DTAs was performed using a combination of orthogonal thiol–yne^44,45^ and CuAAC click chemistries (Scheme 1). First, mPEG-di-NH_2_ was synthesized by thiol–yne reaction between mPEG-propargyl and cysteamine hydrochloride using a previously reported method.^46^ Next, 3-azidopropanoic acid was activated with 4-nitrophenol to obtain 4-nitrophenyl 3-azidopropanoate,^47^ which was coupled to mPEG-di-NH_2_ to obtain mPEG-di-N_3_. The azide functionalized polymer mPEG-di-N_3_ was then reacted with an excess of tripropargylamine by CuAAC to yield branched mPEG-ditriazole-tetrayne. Finally, mPEG-ditriazole-tetrayne was reacted by another CuAAC reaction with an excess of 1-azidooctane, 1-azidodecane, or 1-azidotetradecane to obtain second-generation dendritic amphiphiles DTA-C8, DTA-C10, and DTA-C14, respectively, where the number of carbons in each of the four dendritic alkyl chains of DTA-*Cm* is denoted as m. To verify the conversion of the synthetic steps and the purity of the products, we used ^1^H NMR spectroscopy. Molecular weights and dispersity of the DTAs were determined using size exclusion chromatography (SEC). Additionally, IR spectroscopy was utilized to confirm the complete transformation of mPEG-di-N_3_ into mPEG-ditriazole-tetrayne and to ensure that the final DTAs were free of residual azido-alkanes. All experimental results showed good agreement with the theoretical values (Table 1 and Supporting Information).

**Table 1:** Properties of DTAs and Their Precursors

polymer	*M*_n_ (kDa)^a^	*M*_n_ (kDa)^b^	*Đ*^b^	CMC (μM)^c^	*D*_H_ (nm)^d^	*D*_H_ (nm)^e^
5 kDa mPEG	5.0	5.1	1.03			
mPEG-propargyl	5.2	5.1	1.03			
PEG-di-N_3_	5.4	5.2	1.03			
mPEG-ditriazole-tetrayne	5.7	5.9	1.03			
DTA-C8	6.3	6.1	1.03	20 ± 1	11 ± 2	11 ± 3
DTA-C10	6.4	6.7	1.03	15 ± 1	12 ± 3	13 ± 2
DTA-C14	6.7	6.7	1.03	13 ± 1	16 ± 3	17 ± 5

a *M*_n_ is theoretically calculated.

b *M*_n_ and dispersity of DTA analyzed by SEC using PEG standards.

c CMC determined using the Nile red method.

d Hydrodynamic diameter (*D*_H_) of micelles measured by DLS.

e *D*_H_ of micelles after encapsulating CuSO_4_.

### Characterization of the Self-Assembly of DTAs

To determine if the DTAs can self-assemble into micelles, we first assessed their critical micelle concentration (CMC) using the Nile red (NR) method.^48^ All three amphiphiles exhibited CMC values within a narrow range (13 to 20 μM) (Table 1 and Figure S24) with DTA-C8, which has the least hydrophobic dendron, showing the highest value and DTA-C14, with the most hydrophobic dendron showing the lowest CMC value.

**Figure 1:**
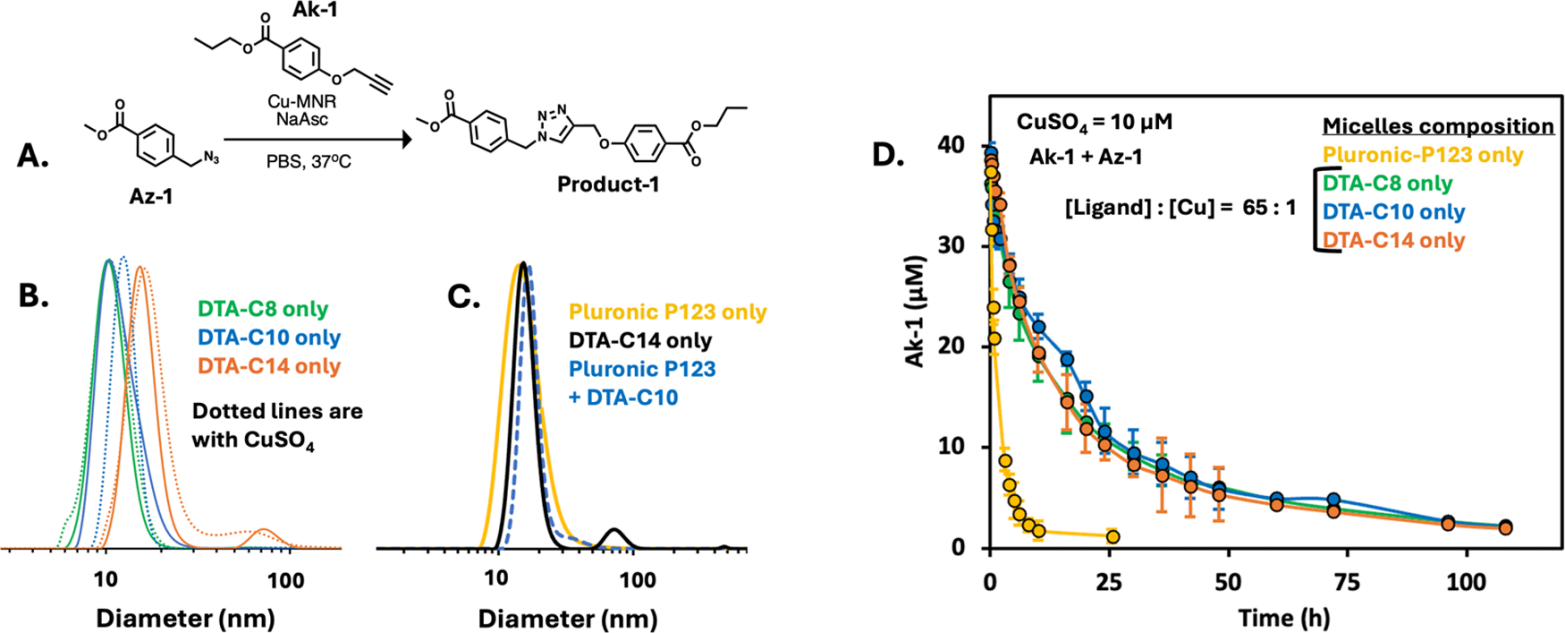
(A) Schematic illustration for the formation of product 1 with Cu-MNRs in PBS. (B) DLS data for micelles formed with DTAs in PBS (DTA = 2 mg/mL). Dotted lines are the sizes of DTA micelles loaded with CuSO_4_ (10 μM). (C) Comparison of hydrodynamic diameter of micelles formed with Pluronic P123 (2 mg/mL, 345 μM), DTA-C14, and Pluronic P123 micelles spiked with DTA-C10 (10 μM). (D) Ak-1 substrate consumption with MNRs made from DTAs and P123. ([amphiphile] = 2 mg/mL, [Ak-1] = 40 ± 5 μM, [Az-1] = 40 ± 5 μM, [NaAsc] = 2 mM, and [CuSO_4_] = 10 μM).

Next, we used dynamic light scattering (DLS) to determine the hydrodynamic diameter (*D*_H_) of the three DTA-based micelles. All three amphiphiles displayed comparable diameters of around 15 nm (Table 1 and Figure 1B) with slight increase in diameter as the number of carbons in the dendritic alkyl chains increases. Upon the addition of CuSO_4_, the micelles exhibited a slight swelling of around 1–2 nm (Table 1 and Figure 1B).

### Effect of DTA Hydrophobicity on CuAAC Reaction Rate in Micelles

Fluorescence measurements for determining the CMC values revealed an increase in NR intensity as the chain length of the DTA’s end-groups increased from octane (C8) to tetradecane (C14) (Figure S24), indicating that micelles self-assembled from DTA-C14 have a relatively higher hydrophobic core compared to those formed from DTA-C8. This prompted us to investigate whether the increase in hydrophobicity of the dendritic blocks of DTA-*Cm* will lead to MNR that will show faster kinetics in the CuAAC reaction. Toward this, we synthesized a pair of substrates: propyl-4-(prop-2-yn-1-yloxy)benzoate (Ak-1) and methyl-4-(azidomethyl)benzoate (Az-1) (Figure 1A). To perform the CuAAC reaction with copper loaded MNRs, micelles were first made by dissolving DTA-*Cm* amphiphiles (0.2 w/v %) in PBS, to which sodium ascorbate (NaAsc, 2 mM) was added, followed by Ak-1 and Az-1 (final [substrates] = 40 ± 5 μM each). The initial substrate concentrations were determined by HPLC. After that, CuSO_4_ was added to the solutions (final [CuSO_4_] = 10 μM), which were then vortexed for 20 s, and the samples were immediately injected into HPLC. The reactions were conducted at 37 °C, and their progress was monitored by HPLC at predetermined time points. The Ak-1 substrate consumption over time is plotted in Figure 1D.

Contrary to previously reported research that highlighted the increased CuAAC reaction rate with tris-triazoles ligands,^16,40^ the reaction rates with the DTA-based micelles were significantly slower and full consumption of the alkyne containing substrate Ak-1 was achieved only after 4 days. Moreover, in contrast to our previous study on depropargylation using palladium loaded MNRs, in which we observed linear correlation between the hydrophobicity of the dendron and the depropargylation reaction rate,^22^ no noticeable differences in the CuAAC reaction rate were observed between micelles made with the three DTA amphiphiles despite their different degrees of hydrophobicity (Figure 1D). Based on these observations, we hypothesized that as all amphiphiles contained the ditris-triazole ligands, the overall tris-triazole to the Cu ratio (∼65:1) was too high and the ligands could significantly compete with the binding of the substrates to the copper center and inhibit the reaction.^31,40^ To test our hypothesis, we conducted a control experiment with micelles made from commercially available Pluronic P123 (P123) amphiphiles, which have micellar size similar to that of the DTAs’ micelles (Figure 1C). We also used these Pluronic P123 micelles to ensure full solubility of the two substrates within the micelles at the tested concentration (Figure S25). Interestingly, the reaction took around 12 h and was much faster (Figure 1D, yellow line) when compared to micelles made from solely DTAs, which as mentioned above took around 4 days to complete. These results support the assumption that the excess of ligands in the DTA-based micelles might be inhibiting the CuAAC reaction. To remedy the catalyst inhibition in DTA-based micelles, we made new micellar formulations by spiking P123-based micelles with DTAs. Formulating micelles this way reduces the net tris-triazole content, while maintaining the same concentration of micelles, which ensures substrate’s solubility. DLS results showed that these DTA spiked micelles have a similar size (around 15 nm) as the micelles formed from either P123 or DTAs (Figure 1C).

**Figure 2:**
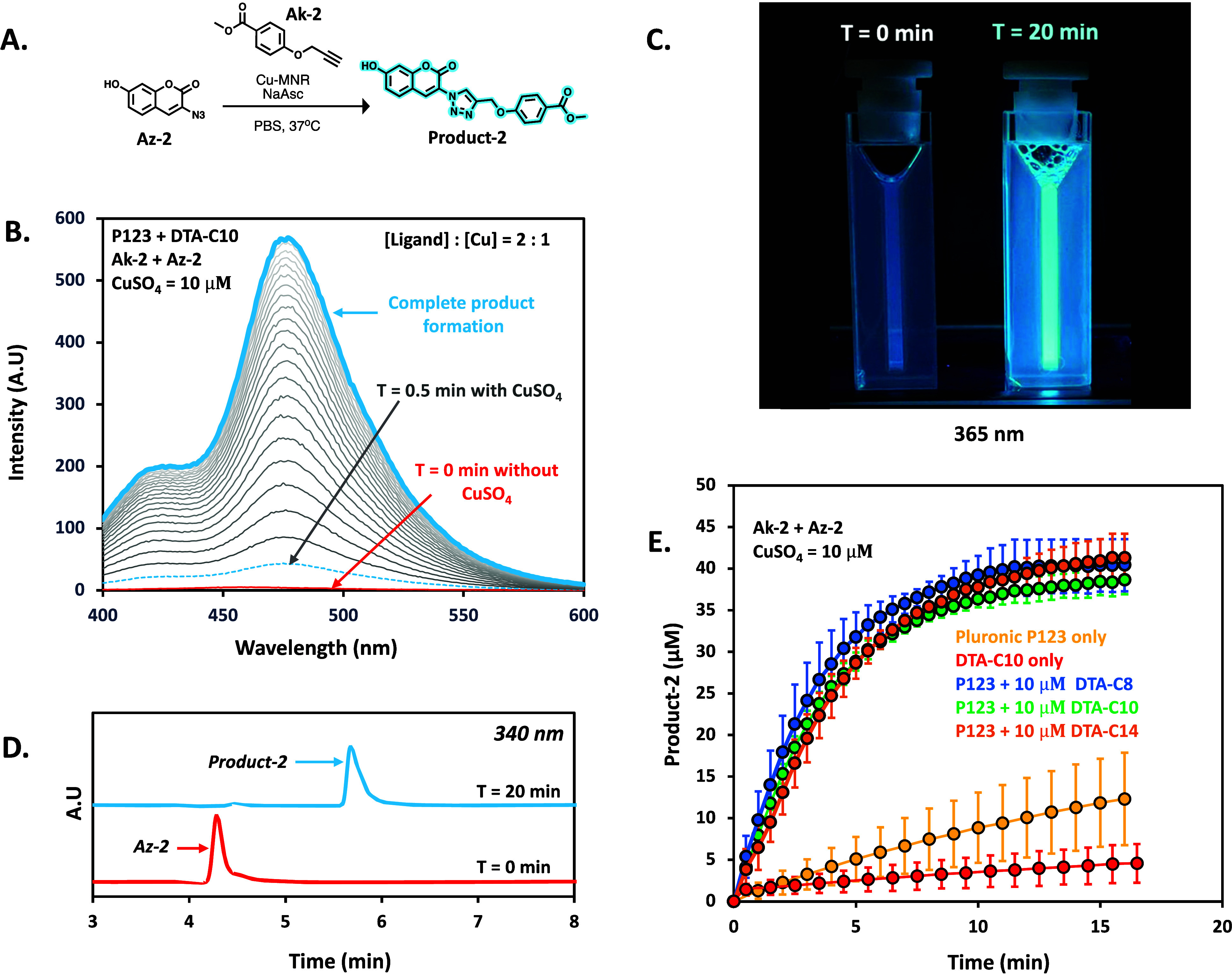
Analysis of product 2 formation with Cu-MNRs. (A) Schematic illustration of product 2 formation with Cu-MNRs in PBS. (B) Fluorescence spectra of product 2 (λ_ex_ = 370 nm, λ_em_ = 475 nm) overtime with Pluronic P123 micelles spiked with DTA-C10 ([P123] = 333 μM, [DTA-C10] = 10 μM, [Ak-2] = 40 ± 5 μM, [Az-2] = 40 ± 5 μM, [NaAsc] = 2 mM, and [CuSO_4_] = 10 μM). (C) Representative photograph of solution taken at time *T* = 0 (before adding CuSO_4_) and after addition at *T* = 20 min (λ_ex_ = 365 nm). (D) Representative HPLC chromatogram overlay (taken at 340 nm). (E) Product 2 formation over time with pure Pluronic P123 micelles, pure DTA-C10 micelles, and Pluronic P123 micelles spiked with different DTAs.

When repeating the CuAAC reaction with a tris-triazole to copper ratio of 2:1, the spiked micellar formulations increased the reaction rate by several fold and the reaction was completed within 20 min. In fact, the reaction was so fast that using HPLC as an analytical method became a limiting factor in monitoring the rate of the reaction. To overcome this challenge, we decided to employ fluorometric analysis as an alternative analytical method due to its significantly higher sampling rate. Taking inspiration from previously reported research by Zimmerman^16^ and Bai,^49^ we synthesized two additional substrates: A profluorogenic 3-azido-7-hydroxy-2H-chromen-2-one (Az-2) and methyl-4-(prop-2-yn-1-yloxy)benzoate (Ak-2). The profluorogenic Az-2 is quenched at the azido state and then can turn into a fluorescent product 2 upon reacting with Ak-2 in CuAAC reaction (Figure 2A), making it an ideal probe for tracking by a fluorimeter.

To carry out the CuAAC reaction with the spiked micelles, P123 was dissolved in PBS (0.2% w/v). To this solution, DTA-Cm stock (dissolved in PBS) was added (final concentration of tris-triazole = 20 μM (∼0.0065% w/v of DTA)), followed by the addition of sodium ascorbate at a final concentration of 2 mM. Subsequently, Ak-2 and Az-2 (in DMSO) were added to yield each of the substrates at a final concentration of 40 ± 5 μM (final DMSO concentration <0.8%), and the micellar solution was briefly vortexed to ensure the mixing of the amphiphiles and the formation of spiked micelles. The initial substrate concentrations in the freshly made solutions were determined using HPLC (Figure 2D) as Az-2 is prone to reduction overtime (Figure S28). To initiate the CuAAC reaction, CuSO_4_ (in PBS) was added to the micellar solutions (10 μM final concentration), and the solution was immediately placed into the fluorimeter and analyzed at different time points (Figure 2B). The rate of product 2 formation was calculated by measuring the difference in intensity at each time point. As can be seen in Figure 2C, the micellar solution in the absence of CuSO_4_ showed negligible florescence (λ_ex_ = 365 nm), while the one with CuSO_4_ showed strong fluorescence after 20 min, indicating the formation of product 2. HPLC chromatograms taken 20 min after the addition of CuSO_4_ further validated the complete conversion of Az-2 to product 2 (Figure 2D). The kinetic data of the formation of product 2 with P123-based micelles spiked with the three DTAs showed a dramatic increase in the reaction rate compared to that of pure P123 micelles (Figure 2E). However, no notable difference in the reaction rates was observed between the DTA-C8, DTA-C10, and DTA-14 spiked MNRs, potentially due to their relative low concentration (∼3 mol %) out of the total amount of amphiphiles composing the MNRs. These results demonstrated that the ditris-triazole ligands, which are part of the molecular framework of the dendritic amphiphiles, can indeed catalyze the CuAAC reaction inside the spiked MNRs. Furthermore, when the experiments were repeated with pure DTA-C10 micelles, the CuAAC reaction proceeded more slowly compared to pure P123 micelles, consistent with the observations for Ak-1 and Az-1 (Figure 2E). These results together with the strong inhibition of the CuAAC reaction when the MNRs were composed solely from DTAs prompted us to expand our research to further uncover how the ratio of polymeric ligand to copper influences the rate of the CuAAC reaction within the MNRs.

### Effect of DTA Concentration on CuAAC Reaction Rates in Micelles

**Figure 3:**
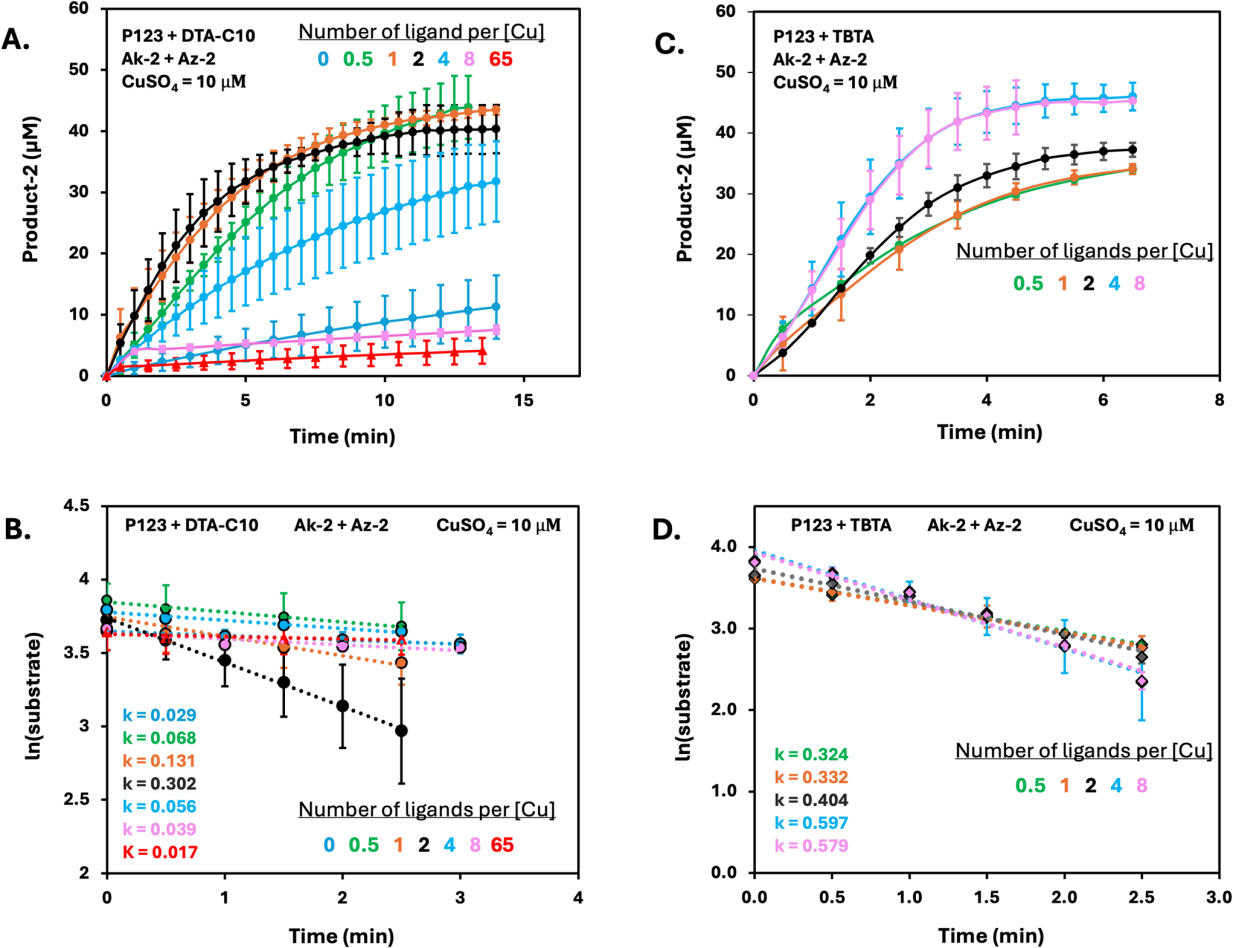
Formation of product 2 with Pluronic P123 micelles spiked with varying amount of DTA-C10 and TBTA. Number of ligands per one Cu ion is shown in different colors. (A) Product 2 formation from P123 and DTA spiked MNRs over time. ([total amphiphile] = 0.2% w/v, [Ak-2] = 40 ± 5 μM, [Az-2] = 40 ± 5 μM, [NaAsc] = 2 mM, and [CuSO_4_] = 10 μM). (B) Natural log of the normalized experimental kinetic data of (A). (C) Product 2 formation from different concentrations of TBTA. ([P123] = 345 μM, [Ak-1] = 40 ± 5 μM, [Az-1] = 40 ± 5 μM, [NaAsc] = 2 mM, and [CuSO_4_] = 10 μM). (D) Natural log of the normalized experimental kinetic data of (C).

As we did not observe any difference in the CuAAC reaction rate by spiking the P123 micelles with DTA-C8, DTA-10, or DTA-C14, we decided to use only DTA-C10 in the next step. To gain more insight about the effect of ligand to the copper ratio on the reactivity of the spiked MNRs, we mixed Pluronic P123 with varying concentrations of DTA-C10, while maintaining overall amphiphile concentration to be 0.2% w/v across all formulations. In brief, CuAAC experiments were conducted using spiked micelles with tris-triazole to copper ratios of 0.5, 1, 2, 4, and 8, where copper concentration was maintained at 10 μM. The detailed experimental setup is provided in the supplementary data. After the addition of CuSO_4_, the reaction was monitored by a fluorometer until a plateau in the fluorescence intensity was reached, indicating complete substrate conversion. Next, each of the samples were immediately analyzed with HPLC to confirm the completion of the reactions (Figures S32–S34). The formation of product 2 with Pluronic P123 micelles spiked with DTA-C10 at varying ratios of tris-triazole to copper is shown Figure 3A. We then converted the product 2 formation into the consumption of Az-2 substrate (Figure S42A) by taking into account its exact initial concentrations as quantified with HPLC (Figures S32–S34). To calculate the reaction rate, a natural log of the substrate’s concentration was plotted against time providing a linear equation correlating with a pseudo first-order reaction ln[*A*]_*t*_ = −*kt* + ln[*A*]_0_. The rate constant (*k*) values were calculated from the slope of the kinetic data (Figure 3B).

**Figure 4:**
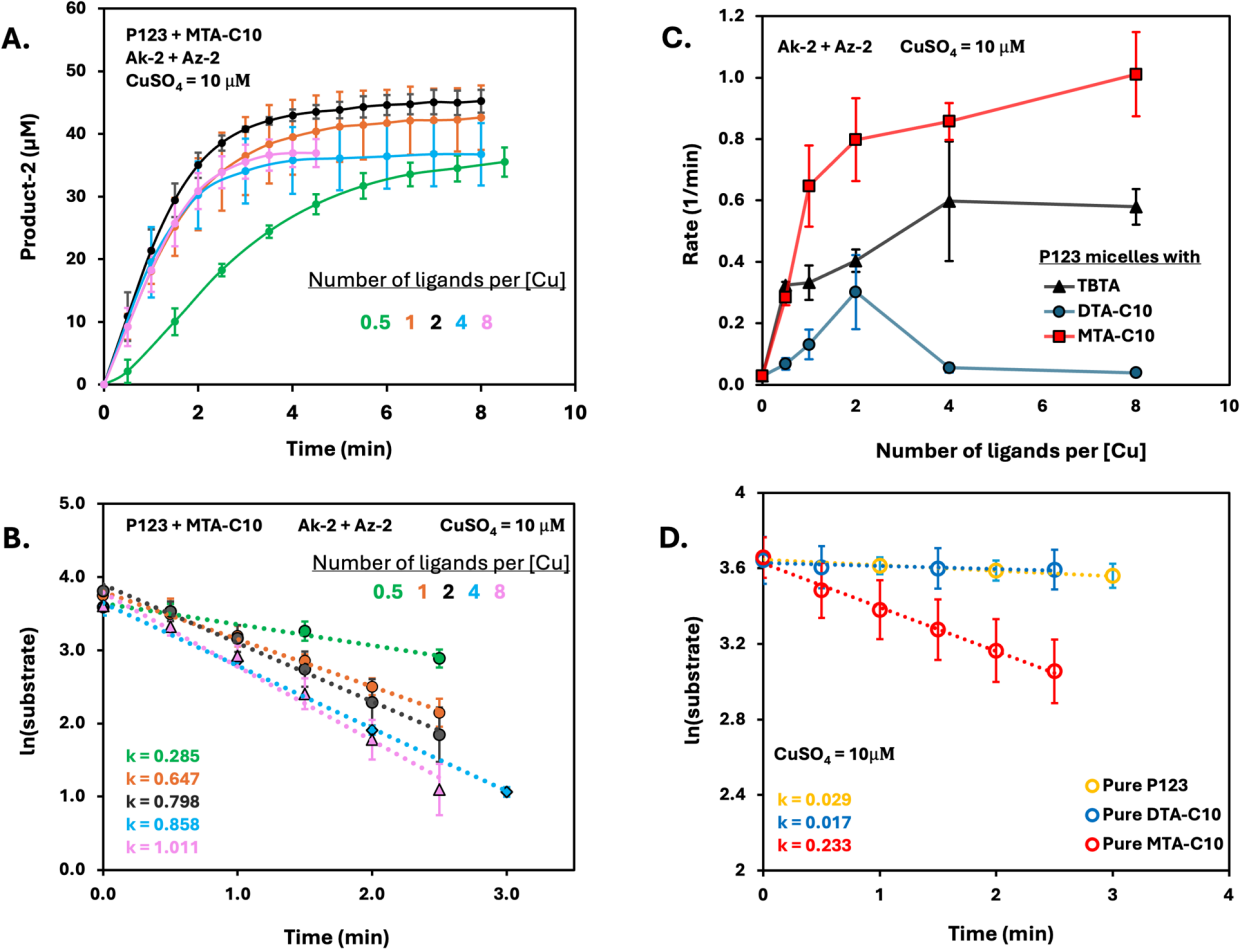
(A) Formation of product 2 with Pluronic P123 micelles spiked with varying amounts of MTA-C10. Number of ligands per one Cu ion is shown in different colors ([total amphiphile = 0.2% w/v], [Ak-2] = 40 ± 5 μM, [Az-2] = 40 ± 5 μM, [NaAsc] = 2 mM, and [CuSO_4_] = 10 μM). (B) Natural log of the normalized experimental kinetic data. (C) Relationship between CuAAC reaction rates and the ratio of [tris-triazole] to [Cu] in the P123–Cu-MNRs loaded with TBTA (black), spiked with DTA-C10 (blue), and spiked with MTA-C10 (red). Error bars are obtained by calculating the rates for the triplicates individually. (D) Initial rate of product 2 formation with pure micelles ([amphiphile = 0.2% w/v], [Ak-2] = 40 ± 5 μM, [Az-2] = 40 ± 5 μM, [NaAsc] = 2 mM, and [CuSO_4_] = 10 μM).

The kinetic results for MNRs composed of Pluronic P123 and spiked with varying amounts of DTA-C10 showed a clear increase in the rate of the CuAAC reaction when the tris-triazole to copper ratio was increased from 0 to 2. However, upon further increasing the tris-triazole to copper ratio from 2 to 4, a significant decline in the rate was observed and the rate dropped even further to nearly zero when the tris-triazole to copper ratio was set to 8 (Figure 3A,B, pink line). These results, which correlate well with the low reactivity that was observed for MNRs composed of solely DTAs (tris-triazole to copper ratio of 65, Figure 3A,B, red line), suggest that the optimum tris-triazole to copper ratio is around 2 for these DTAs. Following this observation, we were curios to compare our ditris-triazole-based amphiphilic ligands with Tris[(1-benzyl-1H-1,2,3-triazol-4-yl)methyl]amine (TBTA), which is one of the most common ligands for accelerating CuAAC reactions.^50^ Although TBTA is not soluble in water and is hence used in polar organic solvents such as DMF or DMSO, we assumed that the Pluronic P123-based micelles will be able to accommodate it. We conducted the CuAAC experiments in a similar manner with varying amounts of TBTA in Pluronic P123 micelles. TBTA was added to the P123 micelles from a stock of DMSO (final concentration of DMSO was less than 0.5% in all experiments). Reaction rates obtained with P123 micelles loaded with varying amounts of TBTA (Figure 3C,D) were significantly faster in comparison with MNR spiked with DTA. Furthermore, increasing the concentration of TBTA did not lead to inhibition of the reaction. Instead, upon increasing the TBTA to copper ratio above 2, the reaction rates increased until reaching a ratio of 4:1, where the rate stayed similar even when the ratio was increased to 8:1 (Figure 4C, black line).

Intrigued by the very different kinetic trends for MNRs containing TBTA, which were nearly independent of ligand to copper ratio, and DTA-C10 spiked MNRs, which showed an optimal ratio of around 2 and twice slower kinetics, we wished to explore a different amphiphilic architecture. We associated the slower kinetics and inhibitory effect of DTA with the close proximity of the two tris-triazole ligands in the case of the DTAs. Based on the observation that the hydrophobicity of the DTAs did not play a noticeable role in the reaction rates, we decided to design a monotris-triazole containing amphiphile (MTA).

### Effect of Amphiphile’s Architecture on CuAAC Reaction Rates in Micelles

**Scheme 2:**
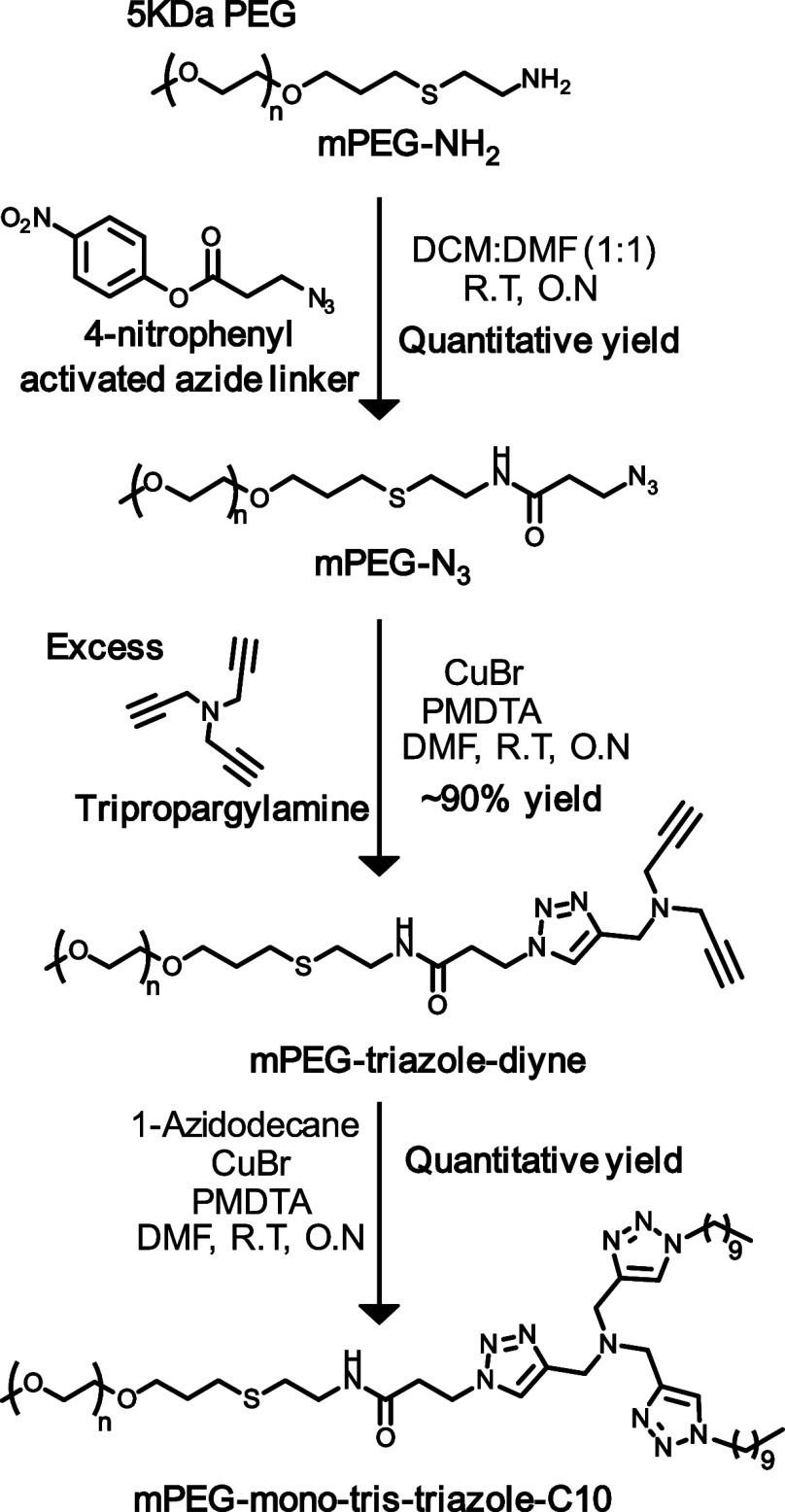
Synthetic Route for MTA-C10

The synthesis of MTA began by reacting mPEG-NH_2_ with a 4-nitrophenol-activated azide linker, generating mPEG-N_3_. This product was then reacted with an excess of tripropargylamine to produce mPEG-triazole-diyne, followed by its reaction with 1-azidodecane, to yield desired MTA-C10 (Scheme 2).

After characterizing MTA-C10 (Table 2), we confirmed its ability to form a spiked MNR when mixed with P123 amphiphiles (Figure S26B). Next, CuAAC reactions were conducted using P123 micelles spiked with MTA-C10, where the tris-triazole to Cu ratios were kept similar to prior experiments (0.5, 1, 2, 4, and 8). The formation of product 2 with P123-based micelles spiked with MTA-C10 is shown in Figure 4A, and the natural log of the substrate’s concentration was plotted against time, which provided a linear equation correlating with a pseudo first-order reaction ln[*A*]_*t*_ = −*kt* + ln[*A*]_0_ (Figure 4B). When the results are compared to those obtained from DTA-C10 spiked MNRs, two notable differences can be clearly seen. First, under the tris-triazole to Cu ratio of 2 to 1, the initial reaction rate became twice faster (Figure 4C). Second, the inhibition of the catalyst observed for the DTA-based micelle formulations was not observed in the case of MNRs spiked with MTA-C10 (Figure 4C). These results validate our hypothesis that the inhibition of the catalyst is likely due to the juxta-positioning of tris-triazoles in the DTA. Furthermore, Pluronic P123 micelles spiked with MTA-C10 outperformed the TBTA-loaded micelles, suggesting that amphiphile-based ligands might be a better choice for performing CuAAC in micellar systems.

**Table 2:** Properties of MTA and Its Precursors

polymer	*M*_n_ (kDa)^a^	*M*_n_ (kDa)^b^	*Đ*^b^
mPEG-mono-N_3_	5.3	5.1	1.03
mPEG-monotriazole-diyne	5.4	5.3	1.03
MTA-C10	5.8	5.8	1.03

a *M*_n_ is theoretically calculated.

b *M*_n_ and dispersity of MTA analyzed by SEC using PEG standards.

To further validate the superiority of the architecture of MTA-C10 over DTA-C10, we prepared MNRs from pure amphiphiles and loaded them with increasing amounts of copper ions. When looking at the obtained kinetic data, we observed that for both types of MNRs, increasing the concentration of copper ions led to increased reaction rates, but the MTA-MNRs remained significantly faster (Figures 4D, S39, S41, and S43). Moreover, when comparing MTA- and DTA-MNRs with similar ligand to copper ratios (i.e., ∼35 and ∼17), the MTA-MNR outperformed the DTA ones despite having half the amount of copper in the former (Figure S43). Another striking observation was the fact that when comparing the pure MTA-MNR to the spiked micelles, both with a copper concentration of 10 μM, the spiked MNRs outperformed the pure MTA-MNR even when the ligand to copper ratio was 0.5 for the spiked ones. These results provide further support to the “less is more” phenomena that was observed for the DTA-MNRs, demonstrating the potential of creating highly reacting MNRs by spiking commodity amphiphiles, such as Pluronic, with tailored made polymeric ligands.

## Conclusions

In this study, we investigated polymeric amphiphiles composed of linear PEG and tris-triazole-based dendritic blocks that can serve as polymeric ligands for conducting CuAAC reactions inside micellar nanoreactors. While MNRs composed of solely DTAs, which contain two tris-triazole ligands, showed poor reactivity, decreasing the ligand to copper ratio by spiking commercially available amphiphile Pluronic P123 with the DTAs, led to the formation of efficient catalytic MNRs. Our results indicate that for these MNRs, which are spiked with a small amount of the polymeric ligands, the hydrophobicity of the polymer has no impact on the reaction rate; however, the ligand to copper ratio plays a significant role on the rate of the reaction with a maximal acceleration at a ratio of 2.

We further show that the architecture of the tris-triazole-based amphiphiles plays a critical role in accelerating the CuAAC reaction rate in MNRs. We hypothesize that the proximity of two tris-triazole moieties in the dendritic block of the DTA can impede the reaction, potentially by forming a tight chelating cage around the copper ion that hinders the approaching of the substrates. To test this, we modified the architecture of the polymeric ligands and synthesized MTA that includes only one tris-triazole unit in its dendritic block. Spiking Pluronic P123-based micelles with MTA led to highly reactive MNRs, which outperformed both the MNRs made from pure MTA and Pluronic P123-based micelles loaded with the widely used TBTA ligand. Taken together, these results provide valuable insights into the use of polymer-based ligands to formulate highly reactive MNRs from readily available amphiphiles.

## Supporting Information Available

Synthetic procedures, amphiphile and micelle characterization data, and detailed experimental protocols and control experiments (PDF)

## Abbreviations

DIPEA: N,N-Diisopropylethylamine
DMPA: 2,2-Dimethoxy-2-phenylacetophenone
DTA: PEG-ditris-triazole amphiphile
MNR: micellar nano reactor
MTA: PEG-moon-tris-triazole amphiphile
PMDTA: N,N,N′,N′′,N′′-Pentamethyldiethylenetriamine
TMs: transition metals
