# Supplementary material for: Micellar “Click” Nanoreactors: Spiking Pluronic-Based Micelles with Polymeric Ligands
Source: Macromolecules. 2024 Nov 4;57(22):10557–66. doi: 10.1021/acs.macromol.4c01425 (PMC11605775; doi:10.1021/acs.macromol.4c01425)
Supplement: Supplementary file 1 — ma4c01425_si_001.pdf [file ma4c01425_si_001.pdf]

Supplementary Information

Micellar "Click" Nanoreactors: Spiking Pluronic Based Micelles With Polymeric Ligands

Krishna Vippala,[1,2,3] Shreyas Shankar Wagle,[1,2] Parul, Rathee,[1,2] Keerthana Mulamukkil,[1,2] Yousif Ayoub,[3] Arthur Komlosh,[3] Sharon Gazal,[3] Bianca Avramovitch,[3] and Roey J. Amir,[1,2,4,5]*

1 - Department of Organic Chemistry, School of Chemistry, Faculty of Exact Sciences, Tel-Aviv University, Tel-Aviv 6997801, Israel

2 - Tel-Aviv University Center for Nanoscience and Nanotechnology, Tel-Aviv University, Tel-Aviv 6997801, Israel

3 - Analytical Technologies Unit R&D, Teva Pharmaceutical Industries, Kfar Saba, Teva, 4410202, Israel

4 - The Center for Physics and Chemistry of Living Systems, Tel-Aviv University, Tel-Aviv 6997801, Israel

5 - ADAMA Center for Novel Delivery Systems in Crop Protection, Tel-Aviv University, Tel-Aviv 6997801, Israel

Table of Contents

Instrumentation and Materials 3

Instrumentation 3

Materials 3

Synthesis 4

Synthesis of azidoalkanes 4

General procedure for tosylating alcohols 4

General procedure for azidation of tosylated alcohols 4

Synthesis of azide linker 6

Synthesis of 3-azidopropanoic acid 6

Synthesis of 4-nitrophenyl 3-azidopropanoate 7

Synthesis of polymeric amphiphiles 9

Synthesis of mPEG-di-N_3 9

Synthesis of mPEG-N_3 10

Synthesis of mPEG-di-triazole-tetrayne 11

Synthesis of mPEG-mono-triazole-diyne 13

Synthesis of mPEG-di-tristriazole C_m 14

Synthesis of mPEG-mono-tristriazole C_{10} 18

Synthesis of azide and alkyne substrates: 20

Synthesis of methyl 4-(prop-2-yn-1-yloxy)benzoate (Ak-2) 20

Synthesis of methyl 4-(azidomethyl)benzoate (Az-1) 20

Synthesis of CuAAC products 21

Characterization of Amphiphiles 27

Size exclusion chromatography (SEC) 27

Infrared spectroscopy (IR) 28

Characterization of DTA micellar structures 29

Critical micelle concentration (CMC) 29

Loading capacity of Pluronic P123 micelles 30

Dynamic light scattering (DLS) 31

Kinetic measurements 32

HPLC measurements 32

Stability of azide-2 33

Reaction progress of product-1 monitored through HPLC 34

Fluorescence measurements and Reaction rates 39

Fluorescence spectra 40

Product-2 formation with Cu-MNRs made from P123 only 44

Product-2 formation with Cu-MNRs from DTA-C10 and MTA-C10 at varying [Cu] Levels 45

Azide-2 consumption with Pluronic micelles spiked with DTA-C10, MTA-C10 and TBTA 45

Azide-2 consumption with Cu-MNRs from DTA-C10 and MTA-C10 at varying [Cu] Levels 46

Cu concentration in micelles 47

References: 48

Instrumentation and Materials

Instrumentation

HPLC: All measurements were recorded on a Waters Alliance e2695 separations module equipped with a Waters 2998 photodiode array detector. HPLC grade solvents were purchased from Bio-Lab Chemicals and were used as received.

^{1}H-NMR: Spectra were recorded on Bruker Avance I and Avance III 400 MHz spectrometers. Chemical shifts are reported in ppm and referenced to the solvent.

SEC: All measurements were recorded on Viscotek GPCmax by Malvern using refractive index detector and PEG standards (purchased from Sigma-Aldrich) were used for calibration.

Fluorescence spectra: CMC measurements were recorded on a TECAN Infinite M200Pro device. Fluorescence Spectra of CuAAC reactions were recorded on an Agilent Technologies Cary Eclipse Fluorescence Spectrometer using quartz cuvettes.

LCMS: Measurements were conducted on a LCMS Xevo–TQD and analysis on Agilent 1260 system with single quadrupole MSD featured with multimode (ESI+APCI) ionization chamber.

ICP-MS: Measurements were conducted on Agilent 7800 ICP-MS equipped with SPS4 Autosampler.

DLS: All measurements were recorded on a Corduan Technology VASCOγ particle size analyser.

Infrared spectra: All measurements were recorded on a Bruker Tensor 27 FT-IR.

Materials

4-dimethylaminopyridine (4-DMAP, 99 %), 4-Nitrophenol (99.5 %), 1-(3- N,N'- dicyclohexylcarbodiimide (DCC, 99%), 1-octanol, 1-decanol, 1-tetradecanol, sodium azide, 1-propyl alcohol, N,N,N′,N′′,N′′-Pentamethyldiethylenetriamine (PMDTA), Copper Bromide (CuBr), Sodium Ascorbate (NaASC), Tris-propargyl amine, 3-Bromopropanoic acid, Copper sulfate pentahydrate ($CuSO_4.5H_2O$), Nile Red, Celite®545, and Sephadex®LH20 were purchased from Sigma-Aldrich. 4-Toluenesulfonyl chloride (TsCl) was purchased from Acros Organics. Diisopropylethylamine (DIPEA), and Sodium bicarbonate ($NaHCO_3$) were purchased from Merck. Silica Gel 60Å, 0.040- 0.063mm, Sodium Hydroxide (NaOH), Sodium Chloride (NaCl), Anhydrous Sodium Sulfate (Na_2SO_4), and all solvents were purchased from Bio-Lab and were used as received. Anhydrous Magnesium Sulfate ($MgSO_4$), and Iodine was purchased from Alfa Aesar. Ammonium Acetate(NH_4Ac) and Ammonium Chloride (NH_4Cl) were purchased from Fischer Chemical. Polytetrafluoroethylene (PTFE) and Nylon syringe filters (13 mm, 0.22 µm) were purchased from Agela Technologies. Deuterated solvents for NMR were purchased from Cambridge Isotope Laboratories (CIL), Inc.

Synthesis

Synthesis of azidoalkanes

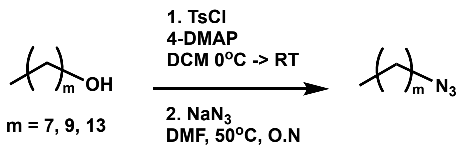

Scheme S1: *Synthetic route for azidoalkanes.*

General procedure for tosylating alcohols

Alcohols (1-octanol, 1-decanol and 1-tetradecanol) were added to a solution of 4-toluenesulfonyl chloride and 4-DMAP dissolved in DCM at $0^{O}C$ and stirred for 15 min. Then the reaction temperature was raised to $25^{O}C$ and stirred overnight. DCM was evaporated and the residual oil was resuspended in ethyl acetate (EtOAC, 50 mL) and transferred into a separating funnel. The organic phase was washed with saturated na_3 (2 x 50 mL) and once with brine (50 mL). The organic phase was dried with anhydrous $MgSO_4$, filtered, and evaporated. The crude product was purified using flash chromatography (EtOAC : Hexanes – 30 : 70 v/v). The tosylated alcohols were obtained as white solids with quantitative yields. Their purity was verified quantitatively with TLC before proceeding to the next step.

General procedure for azidation of tosylated alcohols

Sodium azide (NaN_3) (2 eq) was dissolved in DMF (20 mL DMF for 1 g of NaN_3) and stirred for 5 min. To this solution, tosylated-alcohol (1 eq) was added, and the reaction vessel was heated to $50^{O}C$. The solution was allowed to stir at $50^{O}C$ for overnight under nitrogen atmosphere. The next morning, the solution was allowed to cool down to room temperature. The reaction mixture was then transferred into a separating funnel followed by 100 mL of EtOAc The solution was washed two times with MilliQ H_2O (100 mL each) and then with 50 mL brine. The organic phase was dried with anhydrous Na_2SO_4 for 5 min and the salts were filtered off. Organic solvents were evaporated, and the product was dried under high vacuum.

1-azidooctane: 1-ocanol (2.0 g) was tosylated according to the general procedure above, followed by azidation with sodium azide (2.0 g) to yield 2.3 g (quantitative yield) of the product as yellowish oil.
^{1}H NMR (400 MHz, Chloroform-*d*) δ: 3.24 (t, *J* = 7.0 Hz, 2H, -C**H**$_2$-N$_3$), 1.58 (q, *J* = 7.2 Hz, 2H, -CH$_2$-C**H**$_2$-N$_3$), 1.43 – 1.13 (m, 10H, -CH$_2$-C**H**$_2$-C**H**$_2$-CH$_2$), 0.88 (t, *J* = 6.7 Hz, 3H, -CH$_2$-C**H**$_3$).

1-azidodecane: 1-decanol (2.0 g) was tosylated according to the general procedure above, followed by azidation with sodium azide (1.65 g) to yield 2.22 g (quantitative yield) of the product as yellowish oil.
^{1}H NMR (400 MHz, Chloroform-*d*) δ: 3.24 (t, *J* = 7.0 Hz, 2H, -C**H**$_2$-N$_3$), 1.58 (q, *J* = 7.2 Hz, 2H, -CH$_2$-C**H**$_2$-N$_3$), 1.43 – 1.13 (m, 14H, -CH$_2$-C**H**$_2$-C**H**$_2$-CH$_2$), 0.88 (t, *J* = 6.7 Hz, 3H, -CH$_2$-C**H**$_3$).

1-azidotetradecane: 1-tetradecanol (2.0 g) was tosylated according to the general procedure above, followed by azidation with sodium azide (1.21 g) to yield 2.14 g (quantitative yield) of the product as yellowish oil.
^{1}H NMR (400 MHz, Chloroform-*d*) δ: 3.24 (t, *J* = 7.0 Hz, 2H, -C**H**$_2$-N$_3$), 1.58 (q, *J* = 7.2 Hz, 2H, -CH$_2$-C**H**$_2$-N$_3$), 1.43 – 1.13 (m, 22H, -CH$_2$-C**H**$_2$-C**H**$_2$-CH$_2$), 0.88 (t, *J* = 6.7 Hz, 3H, -CH$_2$-C**H**$_3$).

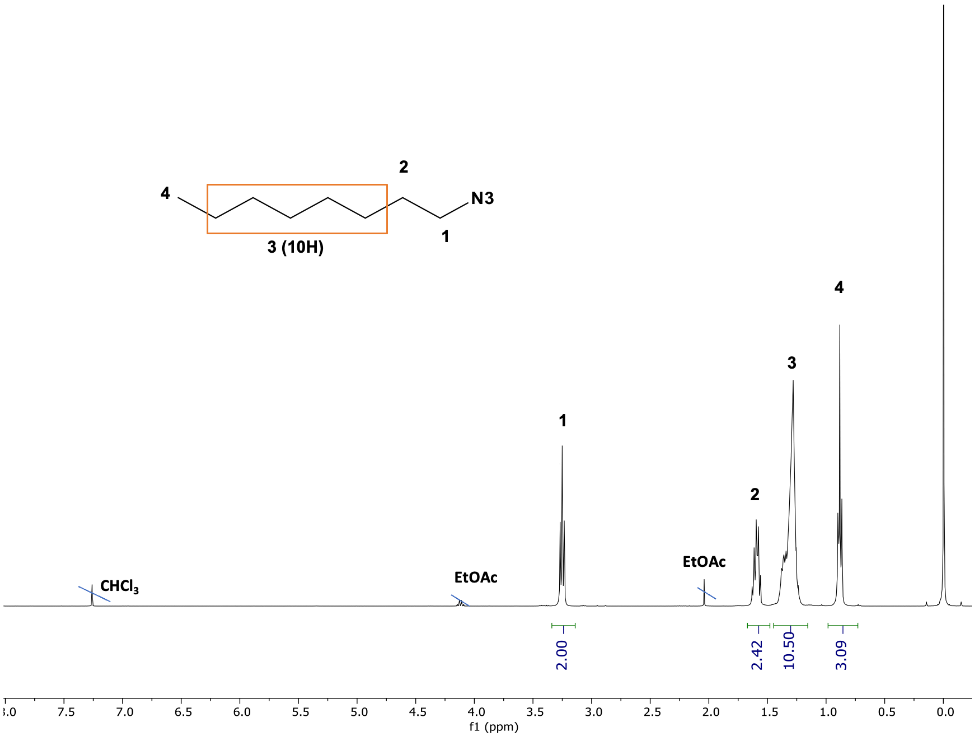

Figure S1: *^{1}H-NMR spectrum of 1- azidooctane in CDCl$_3$.*

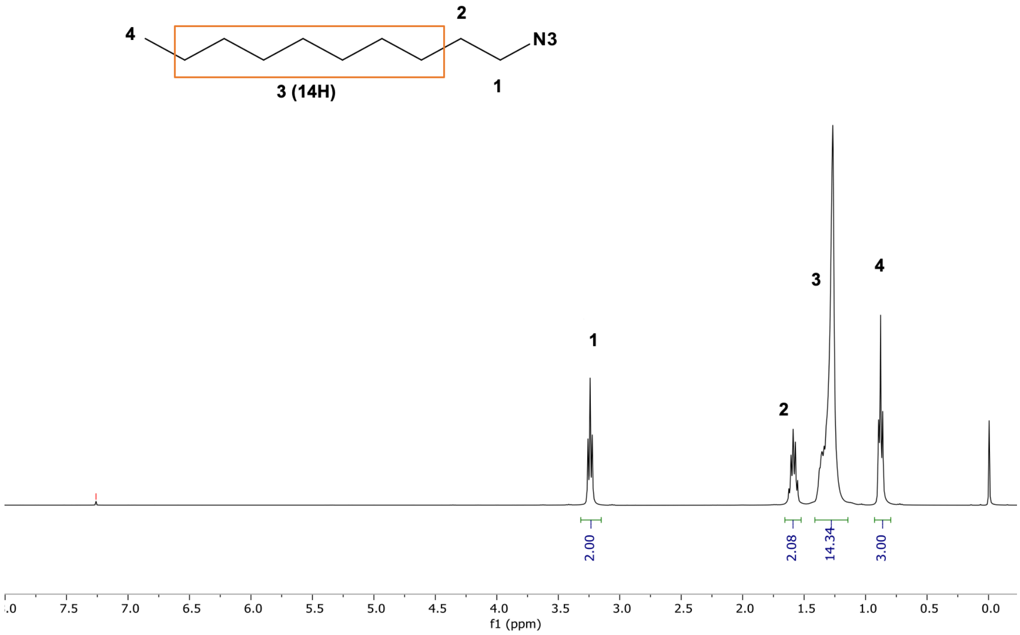

Figure S2: *^{1}H-NMR spectrum of 1- azidodecane in CDCl$_3$.*

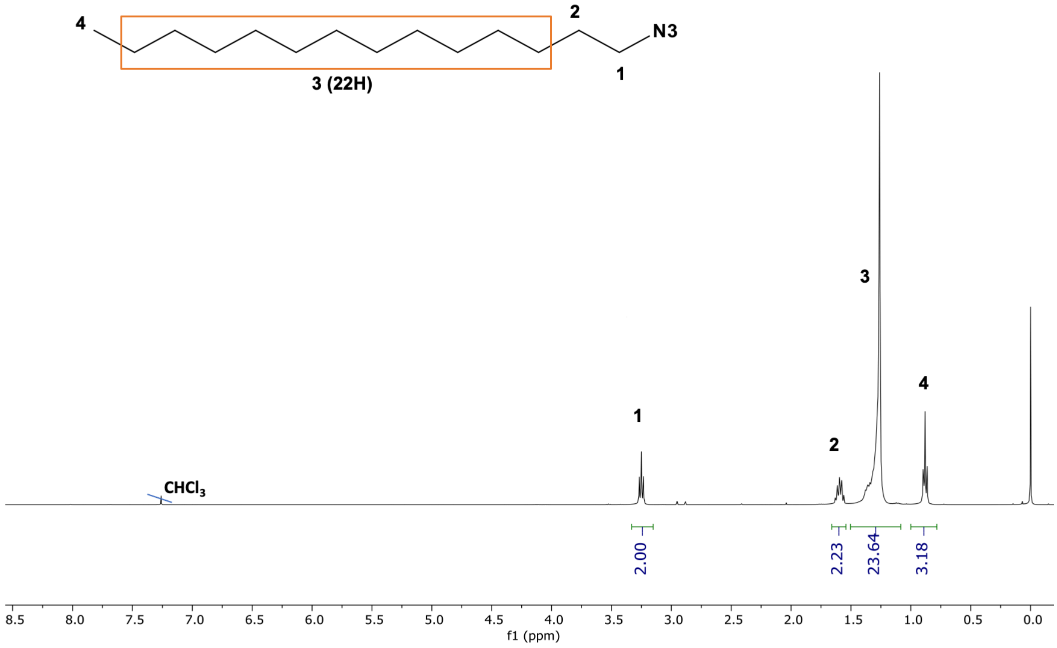

***Figure S3**: ^{1}H-NMR spectrum of 1- azidotetradecane in $CDCl_3$.*

Synthesis of azide linker

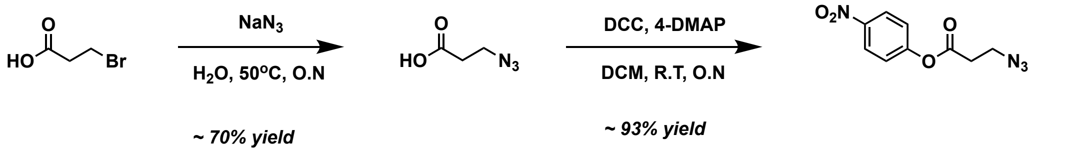

3-bromopropanoic acid **3-azidopropanoic acid** **4-nitrophenyl 3-azidopropanoate**

***Scheme S2:** Synthetic route for 4-nitrophenyl 3-azidopropanoate.*

Synthesis of 3-azidopropanoic acid

3-bromopropanoic acid (1 eq, 1.92 g) and sodium azide (2 eq, 1.8 g) were dissolved in DI water (20 mL) and stirred overnight at 50°C. The reaction solution was transferred to a separating funnel and the product was extracted with DCM (3 x 50 mL). DCM fractions were dried with anhydrous $MgSO_4$ for 5 min and the salts were filtered off. Organic solvents were evaporated, and the yellow liquid was dried under high vacuum. The product was obtained as a yellow liquid (1.01 g) with 70 % yield. All data fits previously reported values.[1]

^{1}H NMR (400 MHz, Chloroform-*d*):δ 3.57 (t, *J* = 6.5 Hz, 1H, -C**H**$_2$-N$_3$), 2.63 (t, *J* = 6.4 Hz, 2H, -C**H**$_2$-COO).

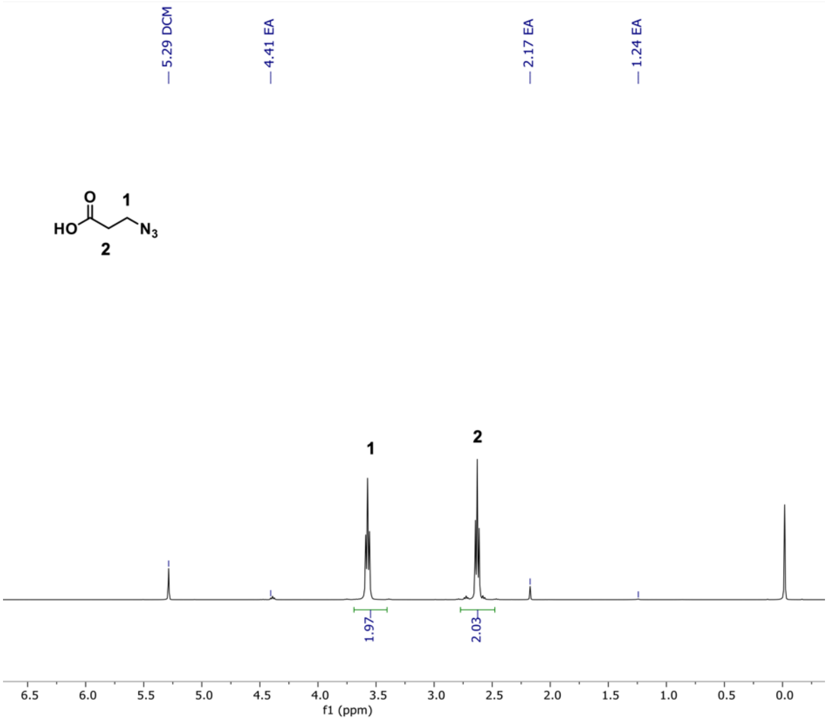

***Figure S4**: ^{1}H-NMR spectrum of 3-azidopropanoic acid in $CDCl_3$.*

Synthesis of 4-nitrophenyl 3-azidopropanoate

3-azidopropanoic acid (1 eq, 987 mg), 4-Nitrophenol (1.1 eq, 1.26 g), DCC (1.1 eq, 1.86 g) and 4-DMAP (0.3 eq, 300 mg) were added to DCM (10 mL) at 5°C. The reaction was stirred at this temperature for 15 min and then the temperature was raised to 25°C and stirred overnight. DCU was filtered off by passing through celite and the crude product was purified with flash chromatography (DCM : Hex 50 : 50 v/v). The fractions containing the product were identified with TLC. Organic solvents were evaporated to yield white solid, which was dried under high vacuum. The product was obtained as white solid (1.88 g), with 93 % yield.

^{1}H NMR (400 MHz, Chloroform-*d*):δ 8.27 (d, *J* = 9.1 Hz, 2H, Ar-H), 7.30 (d, *J* = 9.1 Hz, 2H, Ar-H), 3.71 (t, *J* = 6.3 Hz, 2H, -C**H**$_2$-N$_3$), 2.88 (t, *J* = 6.3 Hz, 2H, -CH$_2$-COO).

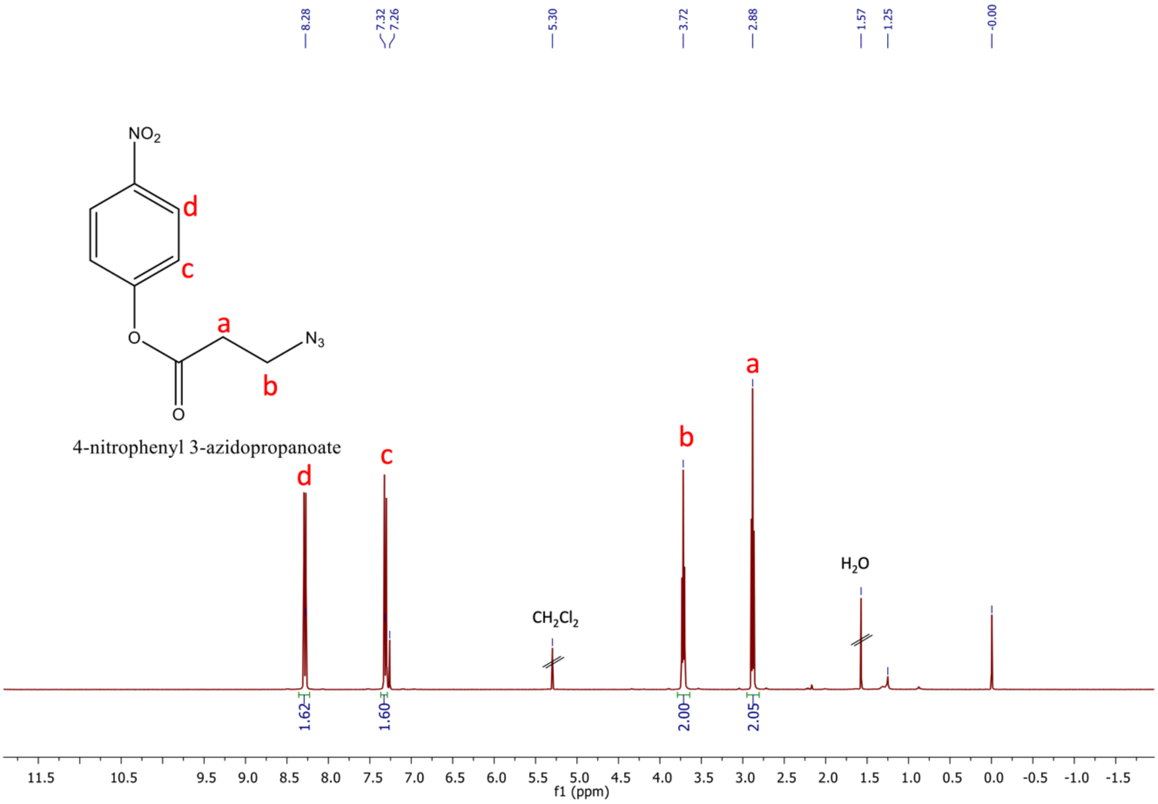

Figure S5: ^{1}H-NMR spectrum of 4-nitrophenyl 3-azidopropanoate in $CDCl_3$.

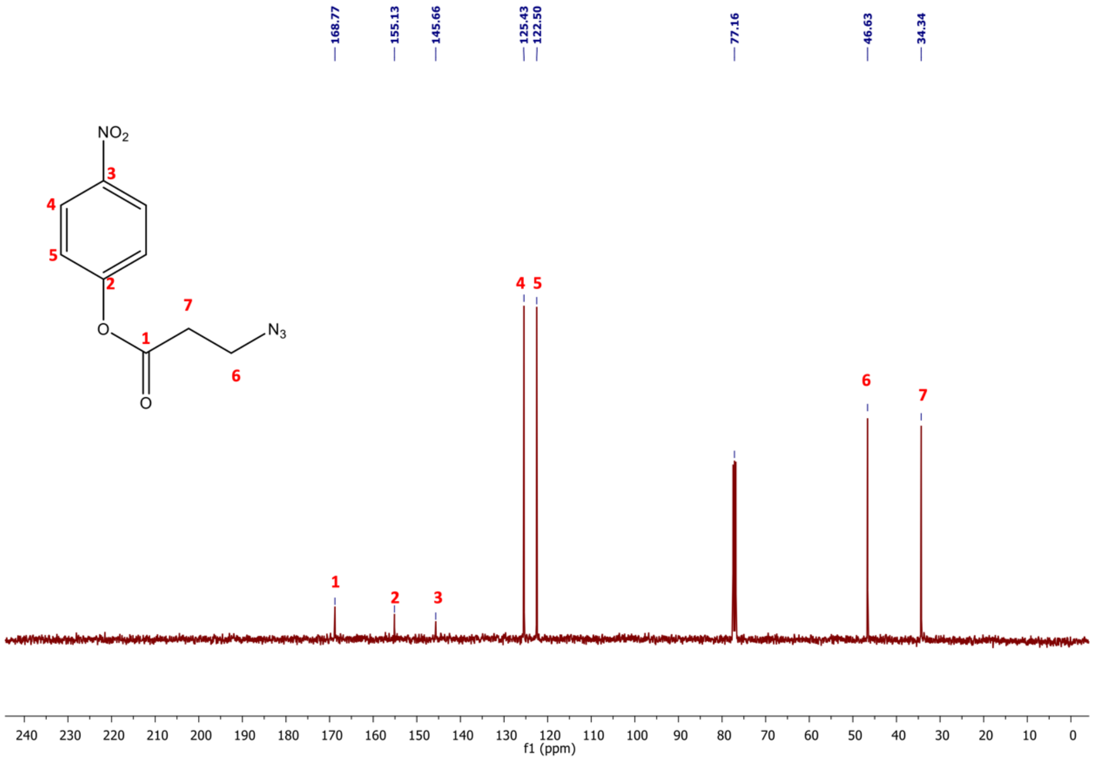

Figure S6: ^{13}C-NMR spectrum of 4-nitrophenyl 3-azidopropanoate in $CDCl_3$.

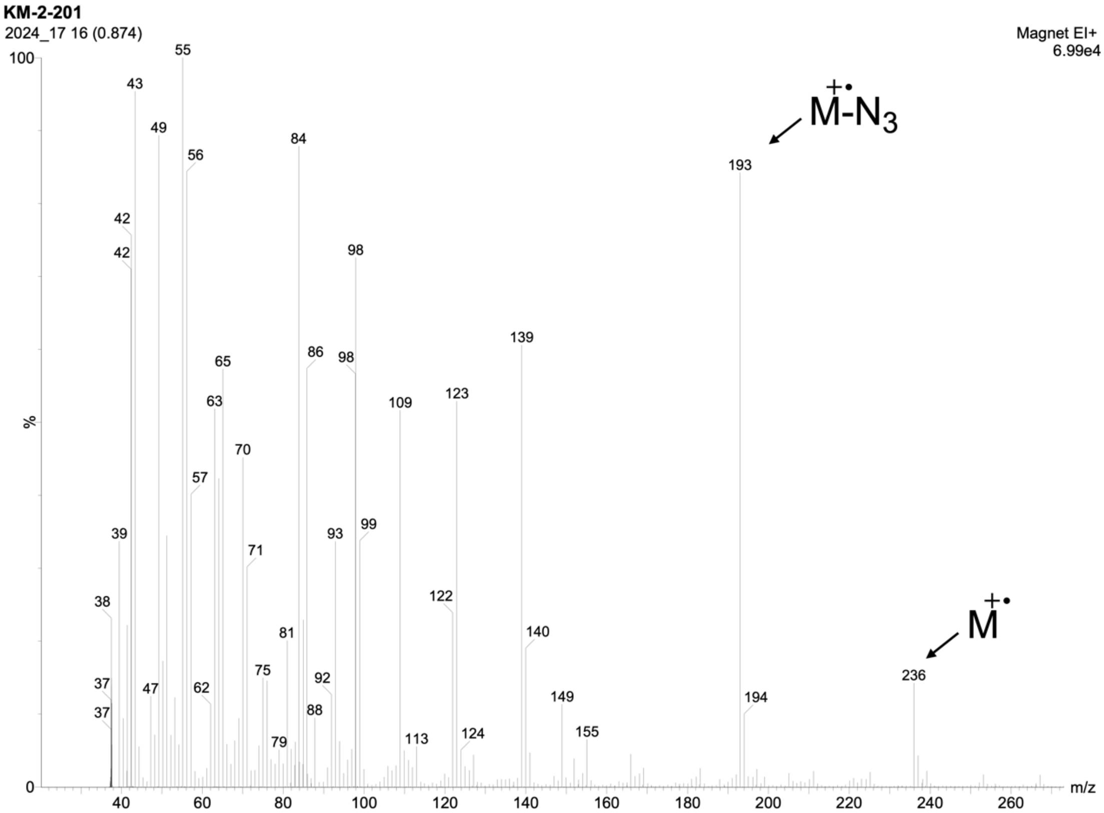

***Figure S7**: Mass spectrum of 4-nitrophenyl 3-azidopropanoate.*

Synthesis of polymeric amphiphiles

mPEG-NH_2 and mPEG-di-NH_2 were synthesised as previously reported,[2] and the spectroscopic characterization correlated well with these reports.

Synthesis of mPEG-di-N_3

mPEG-di-NH_2 (1 eq, 1.0 gr) and 4-nitrophenyl 3-azidopropanate (10 eq, 447 mg) and DIPEA (10 eq, 330 µL) were dissolved in DCM : DMF (1:1 v/v, 2 mL) and stirred at room temperature, overnight. The reaction mixture was loaded as-is on a LH20 (Sephadex®) size exclusion column and eluted with MeOH. Fractions that contained the product (identified by iodine stain) were unified and MeOH was evaporated to dryness. The residue was dried under high vacuum and the product is obtained as a white solid with 90% yield (930 mg).

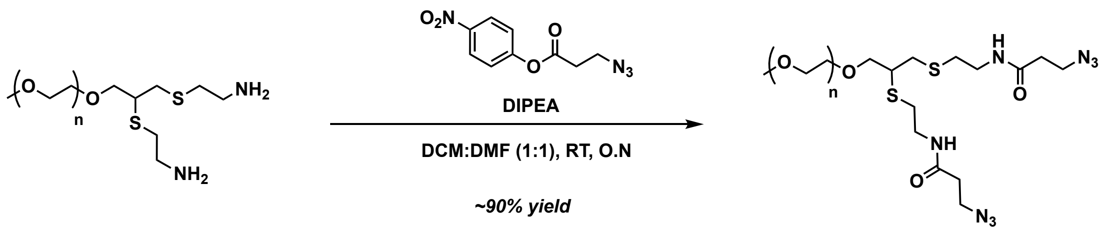

mPEG-di NH2 **mPEG-di N3**

***Scheme S3:** Synthetic route for mPEG-di-N_3.*

^{1}H NMR (400 MHz, Chloroform-*d*): δ 6.78 (s, 1H, -N**H**-CO-), 6.64 (s, 1H, -N**H**-CO-), 4.24 – 3.4 (m, 499H, PEG backbone, -O-C**H**$_2$-, -C**H**$_2$-N$_3$, -C**H**$_2$-NH-CO-), 3.38 (s, 4H, -PEG backbone-C**H**$_3$), 2.99 (p, *J* = 6.3 Hz, 1H, -S-C**H**-), 2.88 – 2.64 (m, 8H, -C**H**$_2$-S-), 2.45 (td, *J = 6.5 Hz*, 4H, -C**H**$_2$-CO-NH).

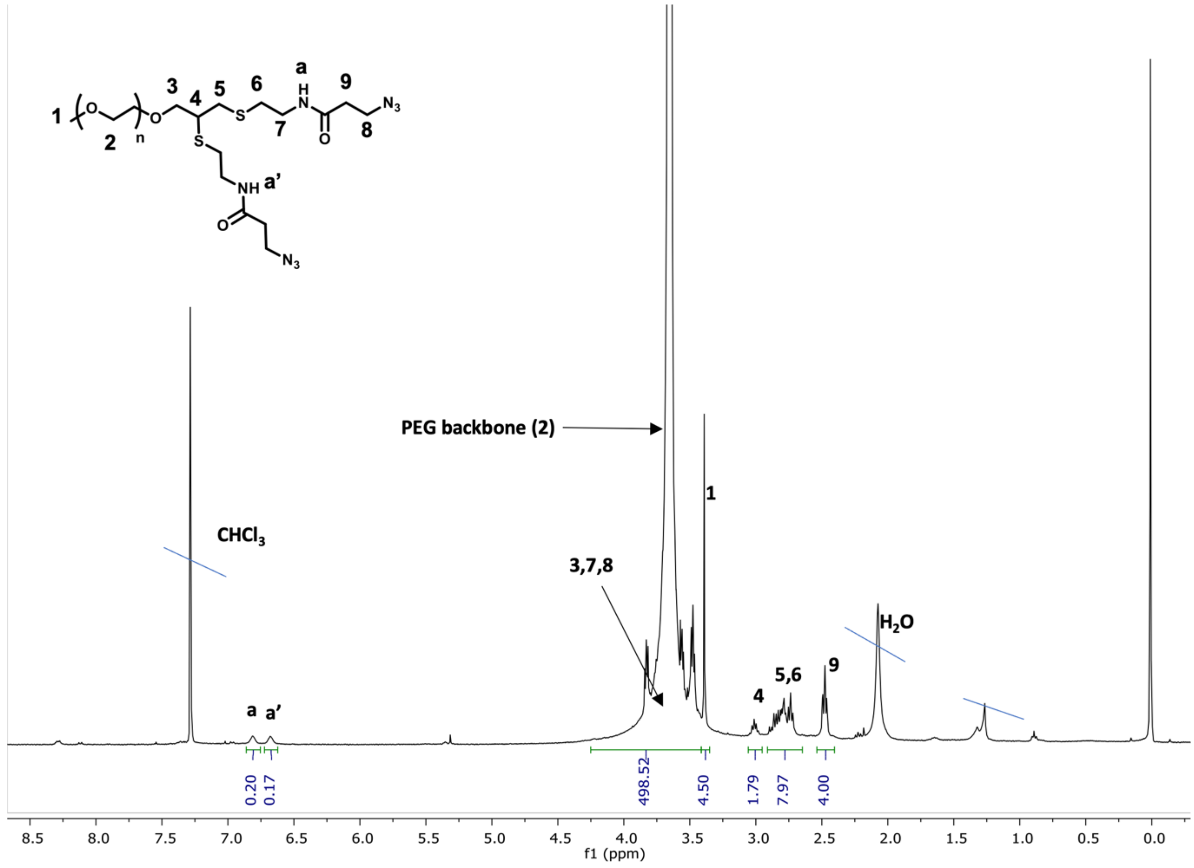

***Figure S8**: ^{1}H-NMR spectrum of mPEG-di-N$_3$ in CDCl$_3$.*

Synthesis of mPEG-N$_3$

mPEG-NH$_2$ (1 eq, 1.0 g) and 4-nitrophenyl 3-azidopropanate (10 eq, 454 mg) and DIPEA (10 eq, 335 µL) were dissolved in DCM : DMF (1:1 v/v, 2 mL) and stirred at room temperature, overnight. The reaction mixture was loaded as-is on a LH20 (Sephadex®) size exclusion column and eluted with MeOH. The initial fractions were collected and pooled until the fractions containing 4-nitrophenol (identified by UV light on TLC) began to elute. Organic solvent in the product pool was evaporated to dryness. The solid residue was dried under high vacuum and the product was obtained as a white solid with 90 % yield (916 mg).

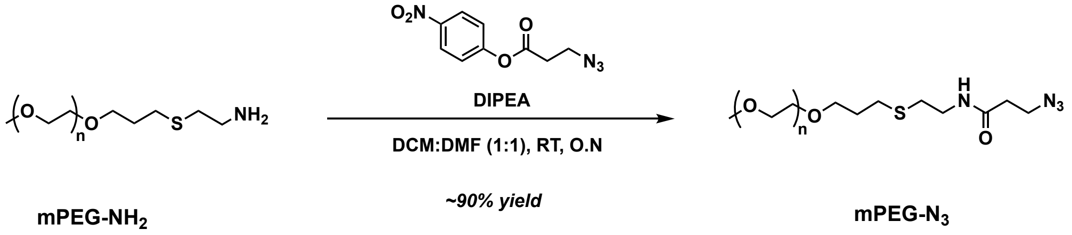

***Scheme S4:** Synthetic route for mPEG-N$_3$.*

^{1}H NMR (400 MHz, Chloroform-*d*) δ 6.25(bs, 1H, -N**H**-CO-), 3.89-3.40 (m, PEG backbone, -O-C**H**$_2$-, -C**H**$_2$-NH-, -C**H**$_2$-N$_3$), 3.38 (s, 3H, -O-C**H**$_3$), 2.76- 2.54 (m, 4H, -C**H**$_2$-S-C**H**$_2$-), 2.44 (t, *J* = 6.4 Hz, 2H, -C**H**$_2$-CO-NH-), 1.91- 1.79 (m, 2H, -O-CH$_2$-C**H**$_2$-CH$_2$-).

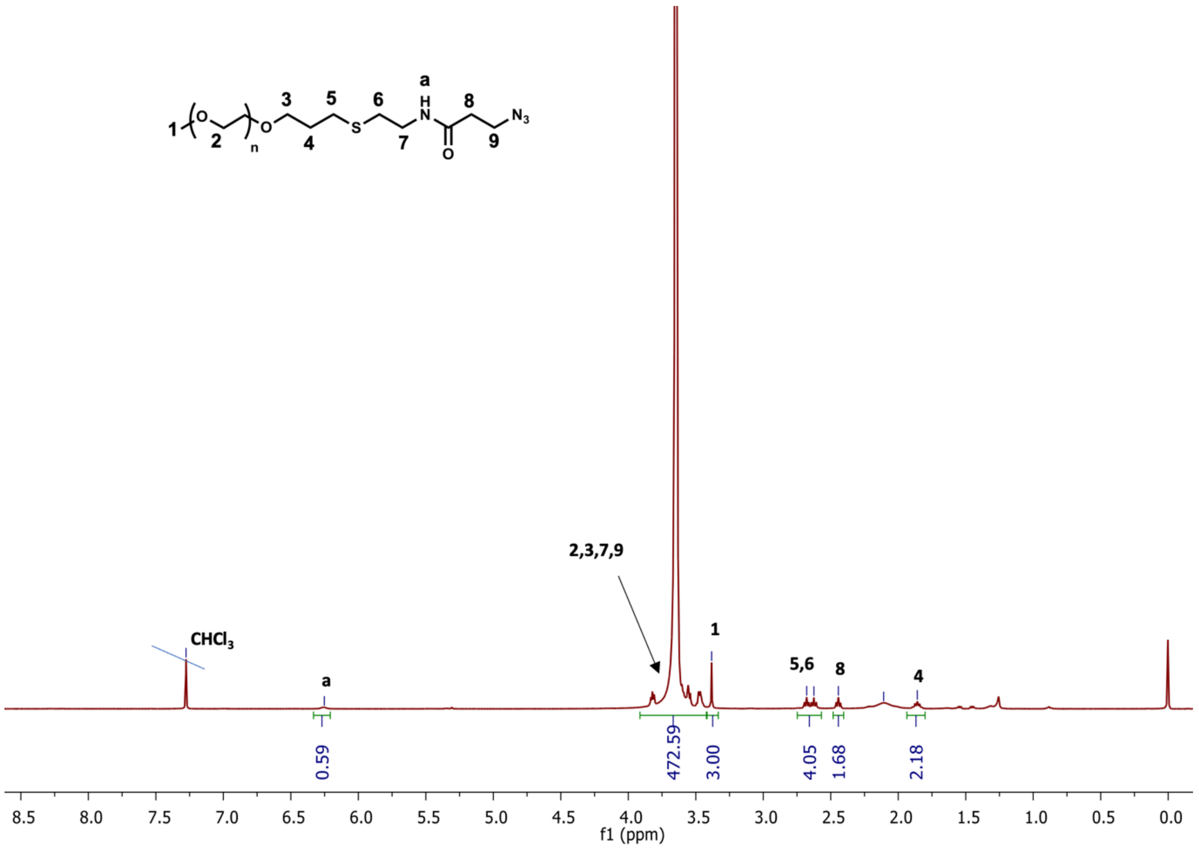

Figure S9: *^{1}H-NMR spectrum of mPEG-N$_3$ in CDCl$_3$.*

Synthesis of mPEG-di-triazole-tetrayne

mPEG-di-N$_3$ (1 eq, 800 mg), tripropargylamine (100 eq, 2.7 mL), and PMDTA (2 eq, 60 µL) were dissolved in DCM : DMF (2 : 1 v/v, 2 mL) and solution was purged with nitrogen for 15 min. To this solution CuBr (1 eq, 21 mg dissolved in DMF (1 mL)) was added under inert conditions and the solutions was stirred overnight at room temperature. The reaction mixture was loaded as-is on a LH20 (Sephadex®) size exclusion column and eluted with MeOH. Fractions that contained the product (identified by iodine stain) were unified and MeOH was evaporated to dryness. The solid residue was dried under high vacuum and the product is obtained as a pale-yellow solid at 90 % yield (750 mg).

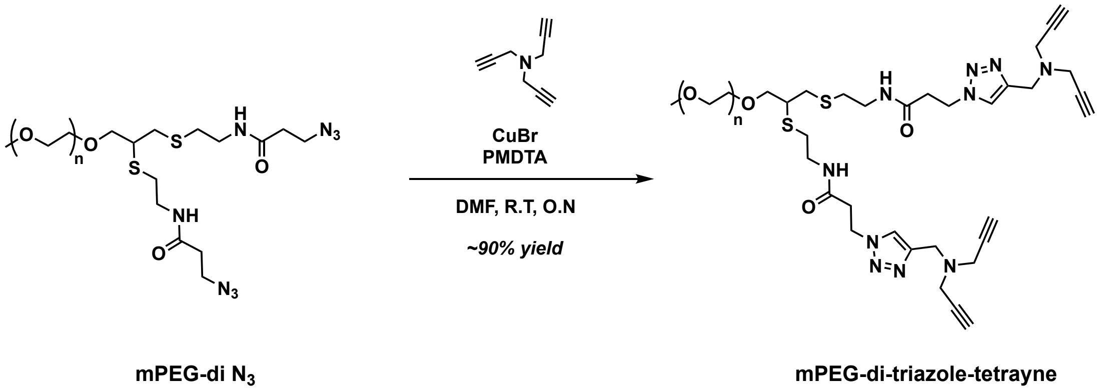

Scheme S5: *Synthetic route for mPEG-di-triazole-tetrayne.*

<u>^{1}H NMR (400 MHz, Chloroform-*d*) δ:</u> 7.63 (s, 2H, N-C**H**=C-), 7.10 – 6.99 (m, 2H, -N**H**-CO-), 4.66 (t, *J* = 6.3 Hz, 4H, -C**H**$_2$-N-N-), 3.89 - 3.37 (m, 675H, PEG backbone, -O-CH$_2$-, -C**H**$_2$-NH-CO-CH$_2$-C**H**$_2$-N-N-, -C**H**$_2$-N-C**H**$_2$-), 3.37 (s, 3H, -O-C**H**$_3$) 2.96 – 2.52 (m, 10H, -S-C**H**-, -C**H**$_2$-S-, -CO-C**H**$_2$-,), 2.27 (s, 4H, -C-C**H**).

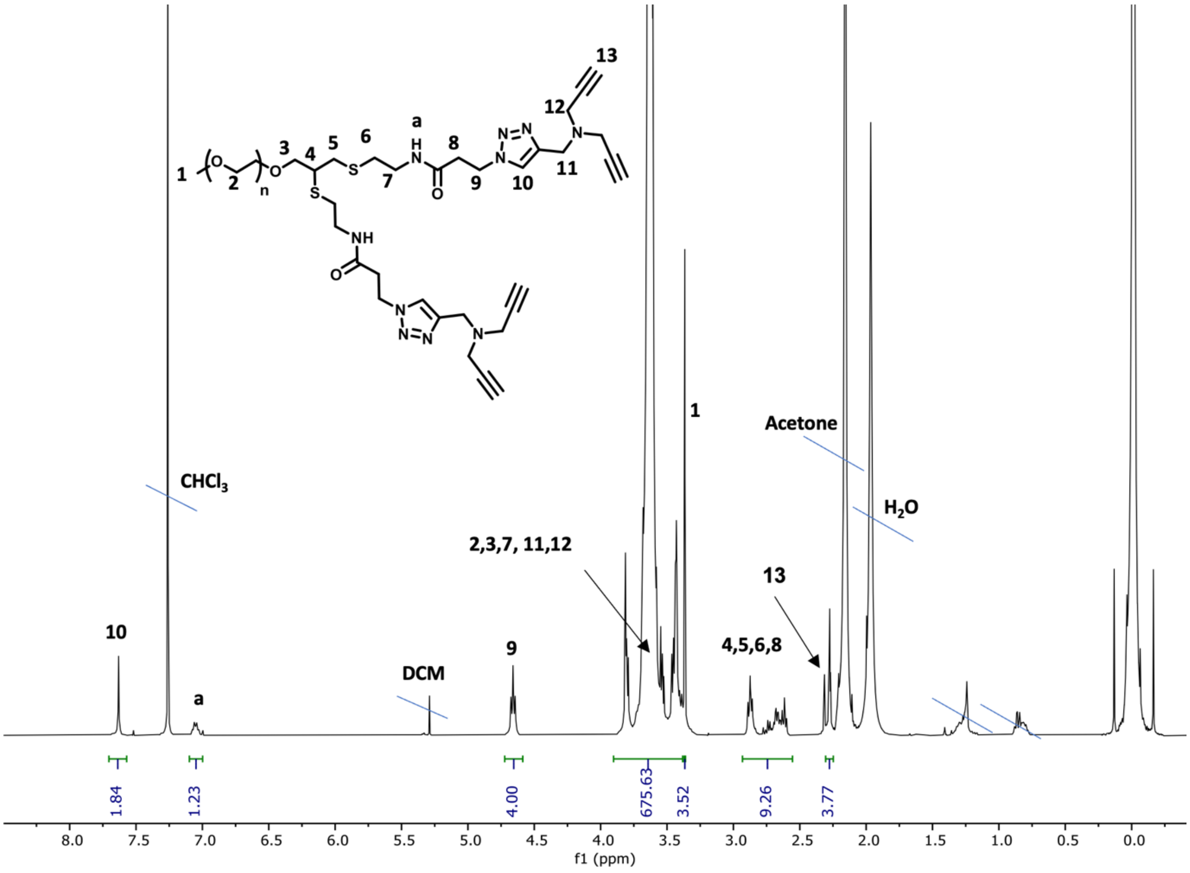

***Figure S10**: ^{1}H-NMR spectrum of mPEG-di-triazole-tetrayne in CDCl$_3$.*

Synthesis of mPEG-mono-triazole-diyne

mPEG-N_3 (1 eq, 200 mg), tripropargylamine (50 eq, 267 µL), and PMDTA (2 eq, 16 µL) were dissolved in DCM : DMF (2 : 1 v/v, 2 mL) and solution was purged with nitrogen for 15 min. To this solution CuBr (1 eq, 5.4 mg dissolved in DMF (1 mL)) was added under inter conditions and the solutions was stirred overnight at room temperature. The reaction mixture was loaded as-is on a LH20 (Sephadex®) size exclusion column and eluted with MeOH. Fractions that contained the product (identified by iodine staining) were unified and MeOH was evaporated to dryness. The solid residue was dried under high vacuum and the product is obtained as a pale-yellow solid with 90 % yield (185 mg).

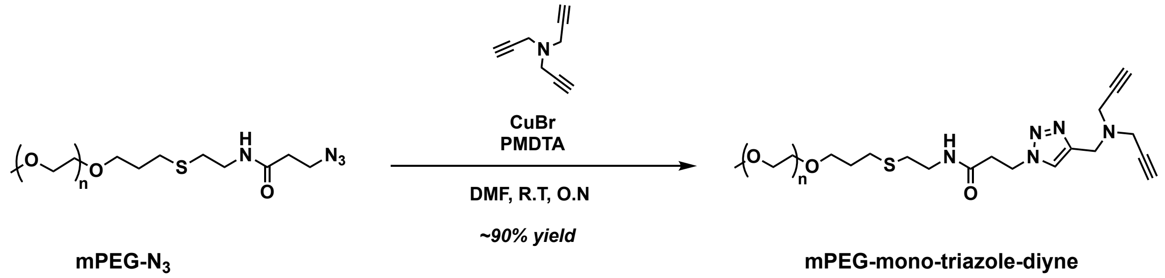

Scheme S6: *Synthetic route for mPEG-mono-triazole-diyne.*

<u>^{1}H NMR (400 MHz, Chloroform-*d*):</u> δ 7.61(s, 1H, -N-C**H**=C-), 6.25(bs, 1H, -N**H**-CO-), 4.66 (t, *J* = 6.1 Hz, 2H, -C**H**$_2$-N-N-), 3.89-3.37 (m, PEG backbone, -O-C**H**$_2$-, -C**H**$_2$-NH-CO-, -C**H**$_2$-N-C**H**$_2$-), 3.37(s, 3H, -O-C**H**$_3$), 2.82 (t, *J* = 6.2 Hz, 2H, -S-C**H**$_2$-CH$_2$-NH-), 2.71- 2.50 (m, 4H, -C**H**$_2$-S- , -NH-CO-C**H**$_2$-), 2.26 (s, 2H, -C-C**H**), 1.88- 1.75 (m, 2H, -O-CH$_2$-C**H**$_2$-CH$_2$-S-).

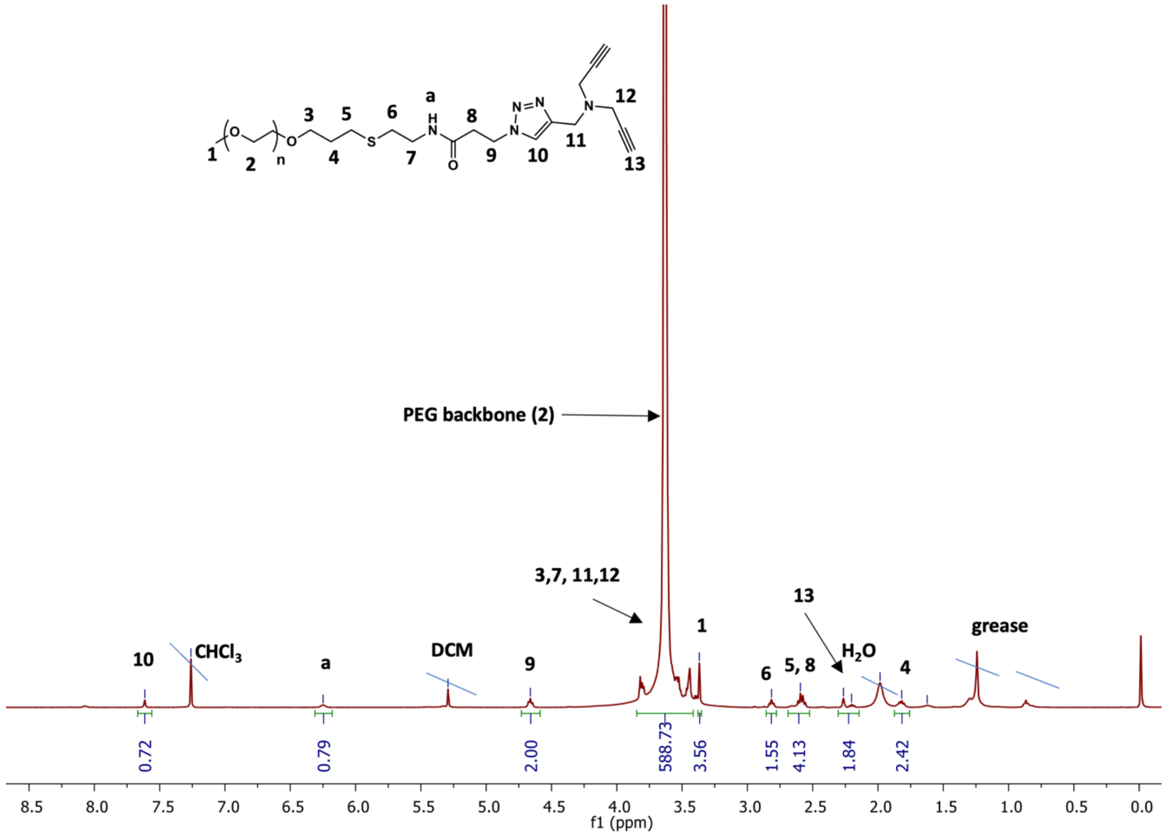

***Figure S11**: ^{1}H-NMR spectrum of mPEG-mono-triazole-diyne in CDCl$_3$.*

<u>Synthesis of mPEG-di-tristriazole C_m</u>

mPEG-di-triazole-tetrayne (1 eq, 200 mg), 1-azdidoalkanes (20 eq) (1-azidoctane (108 mg) or 1-azidodecane (128 mg), or 1-azidotetradecane (167 mg)), and PMDTA (2 eq, 14.6 µL) were dissolved in DCM : DMF (2 : 1 v/v, 2 mL) and solution was purged with nitrogen for 15 min. To this solution CuBr (1 eq (5 mg), dissolved in DMF (1 mL)) was added under inter conditions and the solutions was stirred overnight at room temperature. The reaction mixture was loaded as-is on a LH20 (Sephadex®) size exclusion column and eluted with MeOH. Fractions that contained the product (identified by UV light and/or iodine staining) were unified and MeOH was evaporated to dryness. The solid residues were dried under high vacuum and the products were obtained as a pale-yellow solid with quantitative yields.

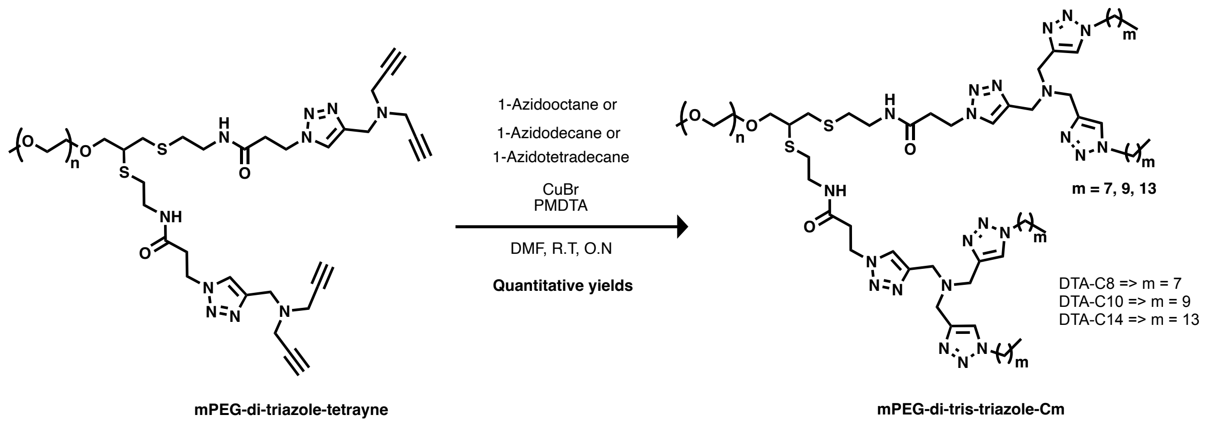

Scheme S7: *Synthetic route for mPEG-di-tristriazole C_m.*

mPEG-di-tristriazole C_8 210 mg.

^{1}H NMR (400 MHz, Chloroform-*d*) δ: 7.8 – 7.7 (m, 6H, N-C**H**=C-, -N**H**-CO), 4.68 (t, *J* = 6.3 Hz, 4H, -C**H**$_2$-N-N-N-C-), 4.33 (t, *J* = 7.3 Hz, 7H, -N-C**H**$_2$-CH$_2$-), 3.89 - 3.37 (m, 390H, PEG backbone, -O-CH$_2$-, -C**H**$_2$-N**H**-CO-CH$_2$-C**H**$_2$-N-N-, -C**H**$_2$-N-C**H**$_2$-), 3.37 (s, 3H, -O-C**H**$_3$) 3.1 – 2.49 (m, 12H, -S-C**H**-, -C**H**$_2$-S-, -CO-C**H**$_2$-), 1.99 – 1.87 (m, 8H, -N-CH$_2$-C**H**$_2$-CH$_2$-), 1.35 – 1.18 (m, 40H, -N-CH$_2$-C**H**$_2$-C**H**$_2$-CH$_3$), 0.95 – 0.76 (m, 12H, CH$_2$-C**H**$_3$).

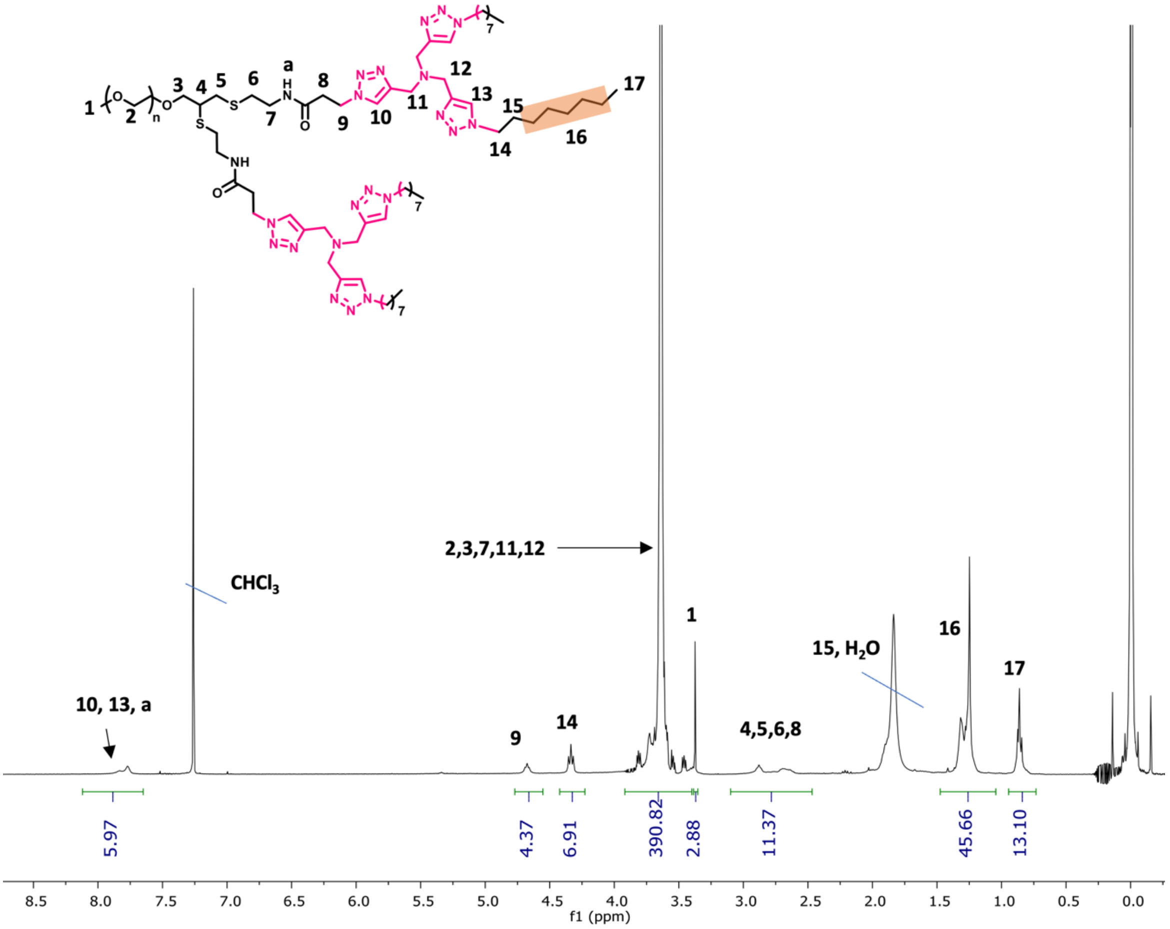

***Figure S12**: ^{1}H-NMR spectrum of mPEG-di-tristriazole C_8 (DTA-C8) in $CDCl_3$.*

mPEG-di-tristriazole C_{10} 214 mg.

^{1}H NMR (400 MHz, Chloroform-*d*) δ: 7.80 (s, 2H, -N-C**H**=C-), 7.74 (s, 4H, N-C**H**=C-), 7.23 – 7.13 (m, 1H,-N**H**-CO), 4.68 (t, *J* = 6.3 Hz, 4H, -C**H**$_2$-N-N-N-C-), 4.33 (t, *J* = 7.3 Hz, 8H, -N-C**H**$_2$-CH$_2$-), 3.89 - 3.37 (m, 672H, PEG backbone, -O-C**H**$_2$-, -C**H**$_2$-N**H**-CO-CH$_2$-C**H**$_2$-N-N-, -C**H**$_2$-N-C**H**$_2$-), 3.37 (s, 4H, -O-C**H**$_3$) 3.1 – 2.55 (m, 13H, -S-C**H**-, -C**H**$_2$-S-, -CO-C**H**$_2$-), 1.99 – 1.87 (m, 10H, -N-CH$_2$-C**H**$_2$-CH$_2$-), 1.35 – 1.18 (m, 56H, -N-CH$_2$-CH$_2$-C**H**$_2$-CH$_3$), 0.95 – 0.76 (m, 12H, CH$_2$-C**H**$_3$).

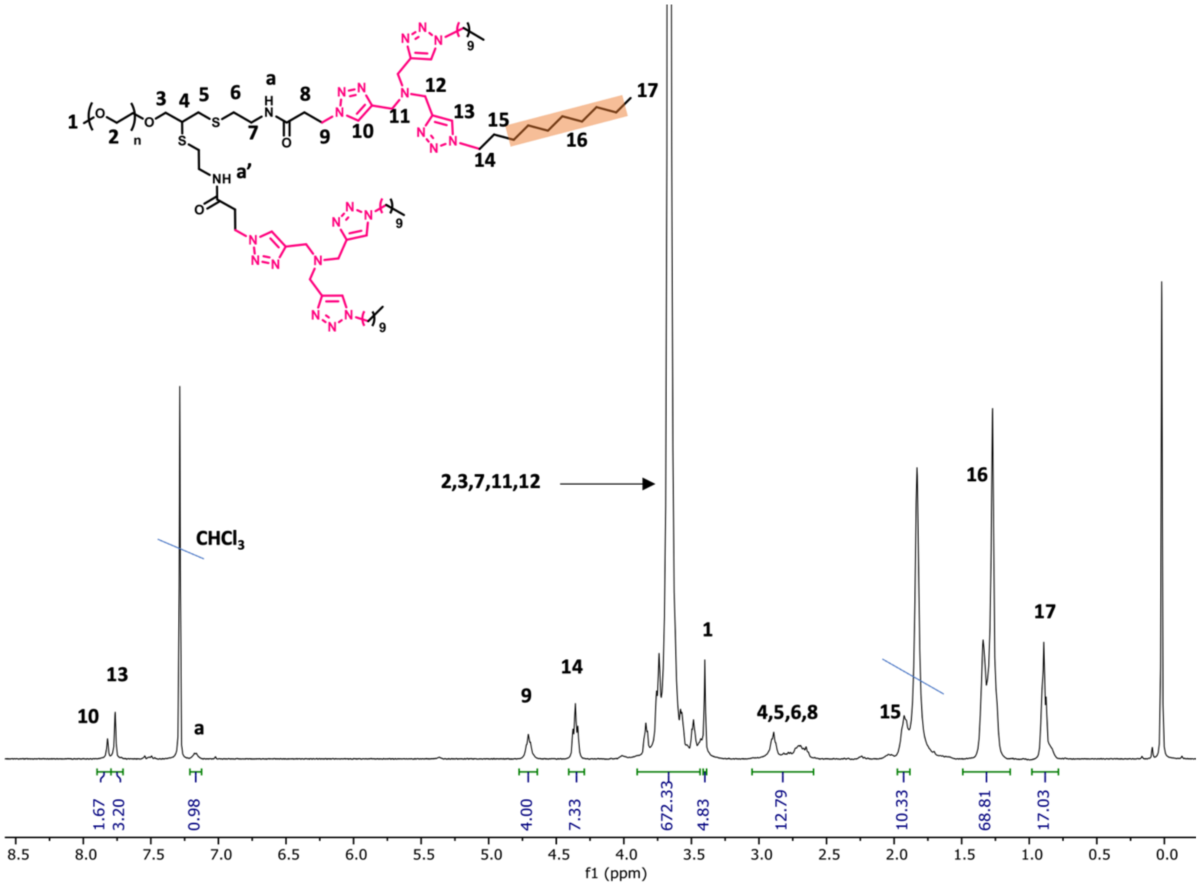

***Figure S13**: ^{1}H-NMR spectrum of mPEG-di-tristriazole C_{10} (DTA-C10) in $CDCl_3$.*

mPEG-di-tristriazole C_{14} 216 mg.

^{1}H NMR (400 MHz, Chloroform-*d*) δ: 7.80 (s, 2H, N-C**H**=C-), 7.74 (s, 4H, N-C**H**=C-), 7.23 - 7.11 (m, 1H,-N**H**-CO), 4.68 (t, *J* = 6.3 Hz, 4H, -C**H**$_2$-N-N-N-C-), 4.33 (t, *J* = 7.3 Hz, 8H, -N-C**H**$_2$-CH$_2$-), 3.89 - 3.37 (m, PEG backbone, -O-C**H**$_2$-, -C**H**$_2$-N**H**-CO-CH$_2$-C**H**$_2$-N-N-, -C**H**$_2$-N-C**H**$_2$-), 3.37 (s, 4H, -O-C**H**$_3$), 2.95 – 2.55 (m, 8H, -S-C**H**-, -C**H**$_2$-S-, -CO-C**H**$_2$-), 1.35 – 1.18 (m, 88H, -N-CH$_2$-C**H**$_2$-C**H**$_2$-CH$_3$), 0.95 – 0.76 (m, 12H, CH$_2$-C**H**$_3$).

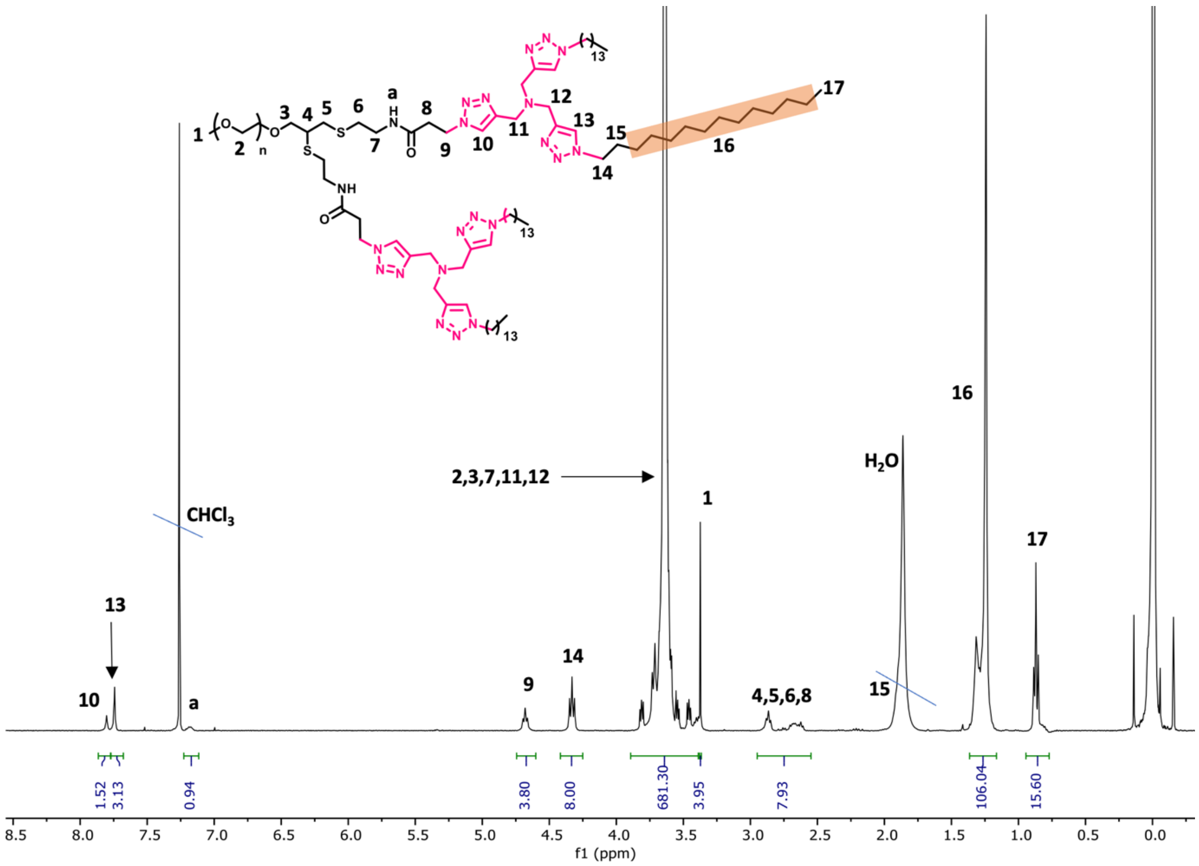

***Figure S14**: ^{1}H-NMR spectrum of mPEG-di-tristriazole C_{14} (DTA-C14) in $CDCl_3$.*

Synthesis of mPEG-mono-tristriazole C_{10}

mPEG-mono-triazole-diyne (1 eq, 100 mg), 1-azidodecane (10 eq, 33 mg), and PMDTA (2 eq, 7.8 µL) were dissolved in DCM : DMF (2 : 1 v/v, 2 mL) and solution was purged with nitrogen for 15 min. To this solution CuBr (1 eq, 2.65 mg, dissolved in DMF (1 mL)) was added under inter conditions and the solutions was stirred overnight at room temperature. The reaction mixture was loaded as-is on a LH20 (Sephadex®) size exclusion column and eluted with MeOH. Fractions that contained the product (identified by iodine stain) were unified and MeOH was evaporated to dryness. The solid residue was dried under high vacuum and the product is obtained as a pale-yellow solid with quantitative yield (100 mg).

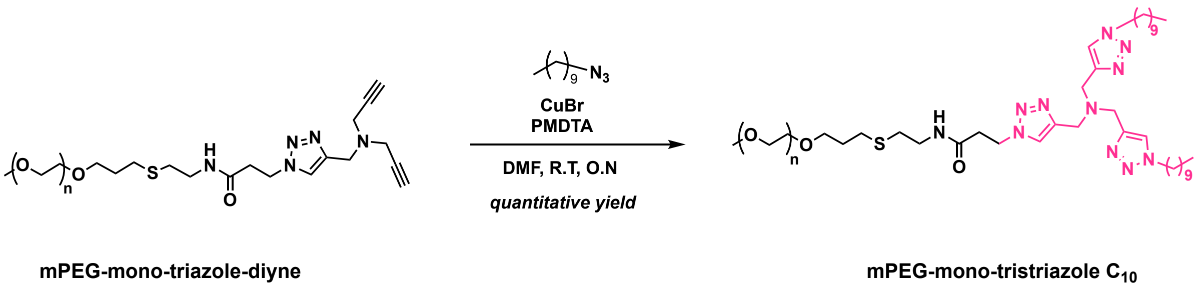

Scheme S8: *Synthetic route for mPEG-mono-tristriazole C_{10}.*

^{1}H NMR (400 MHz, Chloroform-*d*) δ: ^{1}H NMR (400 MHz, $CDCl_3$) δ ^{1}H NMR (400 MHz, $CDCl_3$) δ 7.77 (s, 1H, -N-C**H**=C-), 7.72 (s, 2H, -C=C**H**=N-), 6.30 (bs, 1H, -N**H**-CO-), 4.73-4.59 (m, 2H, CO-CH_2-C**H**$_2$-N-), 4.32 (t, *J* = 7.0 Hz, 4H, Ar-N-C**H**$_2$-CH_2-), 3.93- 3.37 (m, PEG backbone, -O-C**H**$_2$-, -C**H**$_2$-NH-CO-, -C**H**$_2$-N-C**H**$_2$-), 3.36 (s, 3H, -O-C**H**$_3$), 2.89- 2.75 (m, 2H, -S-C**H**$_2$-CH_2-NH-), 2.72- 2.48 (m, 4H, -C**H**$_2$-S- , -NH-CO-C**H**$_2$-,), 1.98- 1.73 (m, 6H, -O-CH_2-C**H**$_2$-CH_2, Ar-N-CH_2-C**H**$_2$-CH_2-), 1.49- 1.08 (m, 28H, -CH_2-C**H**$_2$-CH_2-), 0.92-0.78 (m, 6H, -CH_2-C**H**$_3$).

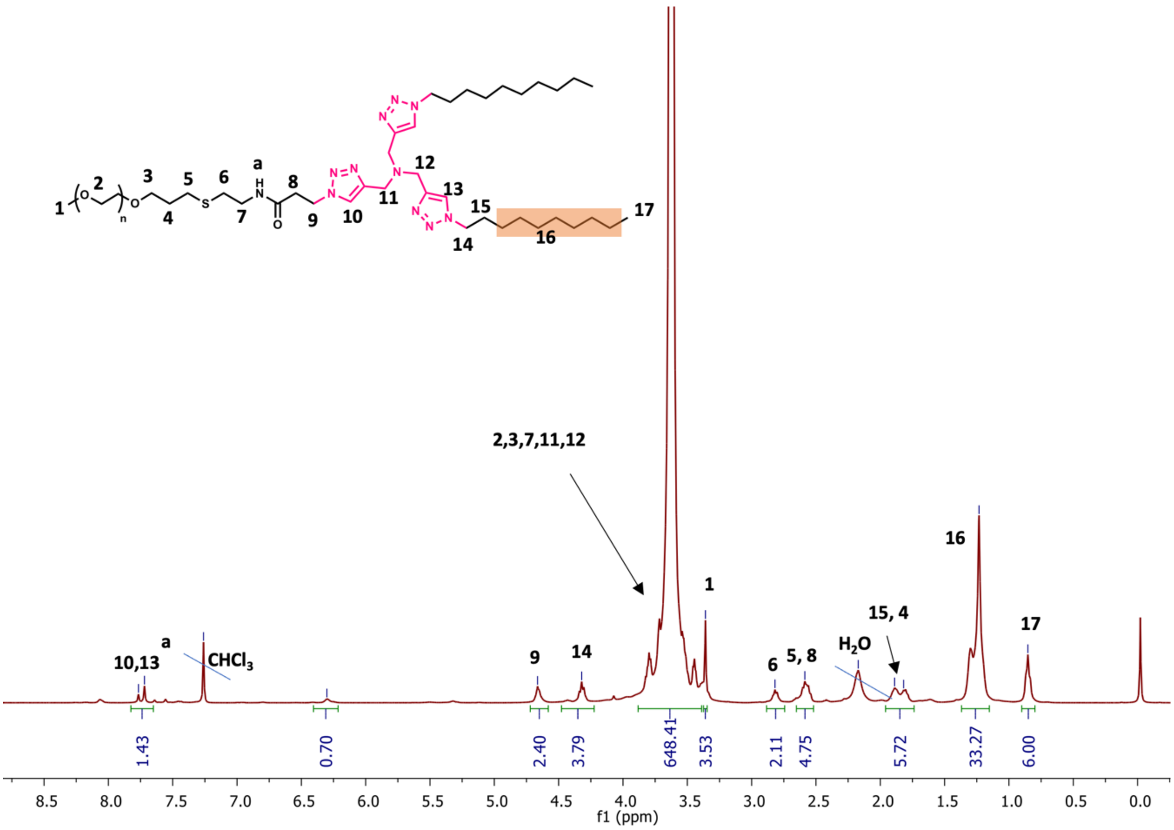

Figure S15: *^{1}H-NMR spectrum of mPEG-mono-tristriazole C_{10} (MTA-C10) in $CDCl_3$.*

Synthesis of azide and alkyne substrates:

propyl 4-(prop-2-yn-1-yloxy)benzoate (Ak-1), was synthesised as previously reported,[2] and the spectroscopic characterization correlated well with these reports.

Synthesis of methyl 4-(prop-2-yn-1-yloxy)benzoate (Ak-2)

First, 4-(prop-2-yn-1-yloxy)benzoic acid was synthesised as previously reported.[2] Then, 4-(prop-2-yn-1-yloxy)benzoic acid (100 mg, 1 eq), methanol (2 eq, 36 mg), DCC (2 eq, 234 mg) and 4-DMAP (2 eq, 138 mg) were dissolved in a DCM (5 mL) and stirred overnight at room temperature. The reaction was filtered through celite, and the celite was washed with DCM. Organic solvents were evaporated to dryness. Product was purified using flash silica chromatography (Hex: EtOAc, 90:10). Product was obtained as a viscous oil in 86 % yield (450 mg). The spectroscopic characterization correlated well with these reports.[3]

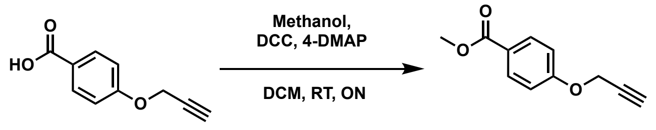

4-(prop-2-yn-1-yloxy)benzoic acid   methyl 4-(prop-2-yn-1-yloxy)benzoate (Ak-2)

Scheme S9: Synthetic route for methyl 4-(prop-2-yn-1-yloxy)benzoate (Ak-2).

Synthesis of methyl 4-(azidomethyl)benzoate (Az-1)

Methyl 4-(bromomethyl)benzoate (1 eq, 100 mg) and sodium azide (2 eq, 56 mg) were dissolved in DMF (5 mL) and the reaction was stirred overnight at 50°C. Then, the reaction mixture was transferred to a separating funnel followed by EtOAc (20 mL). The organic phase was washed with saturated $NaHCO_3$: brine (1 : 1 v/v) twice and the organic layer was dried with anhydrous Na_2SO_4. The organic fraction was evaporated to dryness and the residue was dried under high vacuum. Product is collected as clear liquid with 92 % yield (76 mg). The spectroscopic characterization correlated well with these reports.[4]

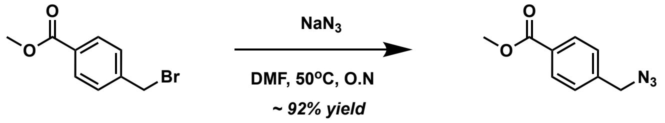

methyl 4-(bromomethyl)benzoate   methyl 4-(azidomethyl)benzoate (Az-1)

Scheme S10: Synthetic route for methyl 4-(azidomethyl)benzoate (Az-1).

^{1}H NMR (400 MHz, Chloroform-*d*) δ 8.05 (d, *J* = 8.3, Hz, 2H, Ar-**H**), 7.39 (d, *J* = 8.3 Hz, 2H, Ar-**H**), 4.41 (s, 2H, -C**H**$_2$-N$_3$), 3.92 (s, 3H, C**H**$_3$-O-).

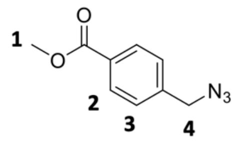

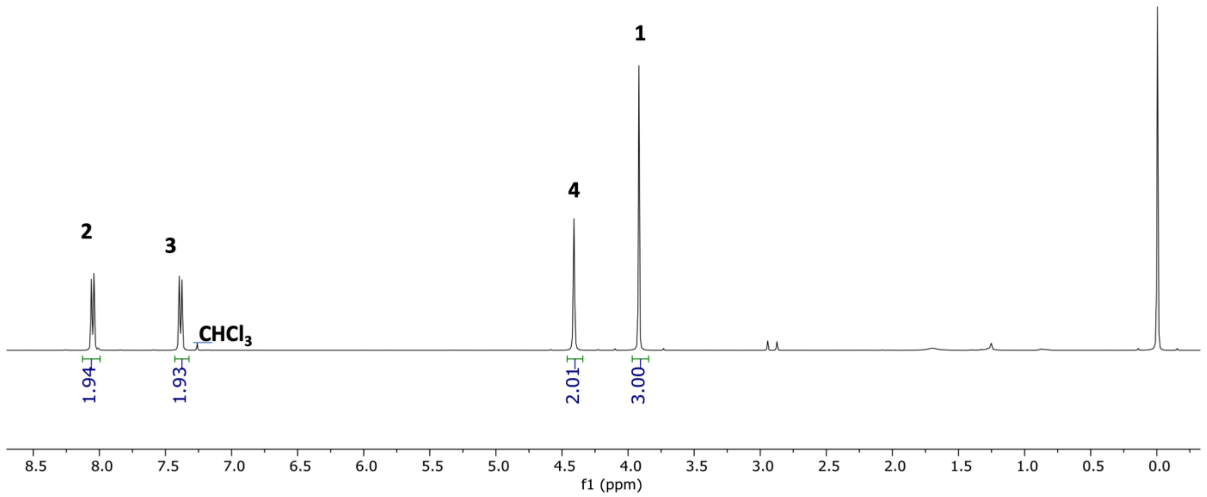

***Figure S16**: ^{1}H-NMR spectrum of Az-1 in CDCl$_3$.*

3-azido-7-hydroxy-coumarin (Az-2) was synthesised as previously reported,[5] and the spectroscopic characterization correlated well with these reports.

Synthesis of CuAAC products:

Procedure for making product-1:

Ak-1 (80 mg, 1 eq), Az-1 (84 mg, 1.2 eq), and NaASC (72 mg, 1 eq) were dissolved in a DMF : H2O mixture (4:1 v/v). The solution was purged with nitrogen for 10 minutes. $CuSO_4 \cdot 5H_2O$ (58 mg, dissolved in 1 mL H_2O) was then added under inert conditions, and the mixture was stirred at room temperature for one hour. Following this, the solution was added to 100 mL of saturated NH_4Cl solution, and the crude product was extracted three times with 50 mL of DCM. The combined DCM extracts were dried over $MgSO_4$. DCM was evaporated and the crude product was purified by flash chromatography using a 50:50 v/v mixture of EtOAc : hexanes. The final product (Product-1) was obtained as a white solid in 81% yield.

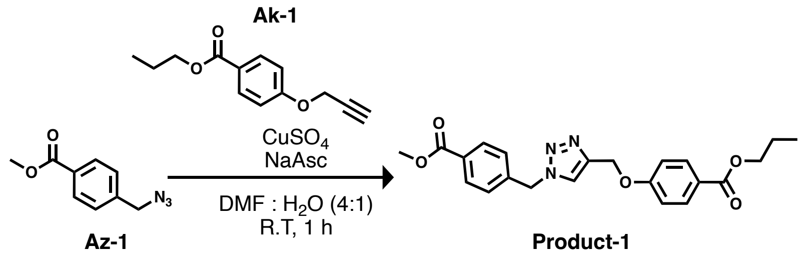

Scheme S11: *Synthetic route for CuAAC product-1.*

^{1}H NMR (400 MHz, Chloroform-*d*) δ: 8.03 (d, 2H, J = 8.3 Hz, 2H, Ar-**H**), 7.98 (d, 2H, J = 8.8 Hz, Ar-**H**), 7.57 (s, 1H, Triazole-H), 7.31 (d, J= 8.4 Hz, 2H, Ar-**H**), 6.98 (d, J= 8.9 Hz, 2H, Ar-**H**), 5.59 (s, 2H, -N-C**H**$_2$-Ar-), 5.24 (s, 2H, -O-C**H**$_2$-C=C-), 4.24 (t, *J* = 6.7 Hz, 2H, C**H**$_2$-O-CO-), 3.91 (s, 3H, -O-C**H**$_3$), 1.82 – 1.71 (m, 3H, CH$_3$-C**H**$_2$-), 1.01 (t, *J* = 7.4 Hz, 3H, C**H**$_3$-CH$_2$-).

^{13}C NMR (100 MHz, CDCl$_3$) δ 166.44, 161.85, 144.40, 139.31, 131.72, 130.82, 130.55, 128.22, 128.01, 123.73, 122.95, 114.43, 66.45, 62.18, 53.93, 52.45, 22.27, 10.66.

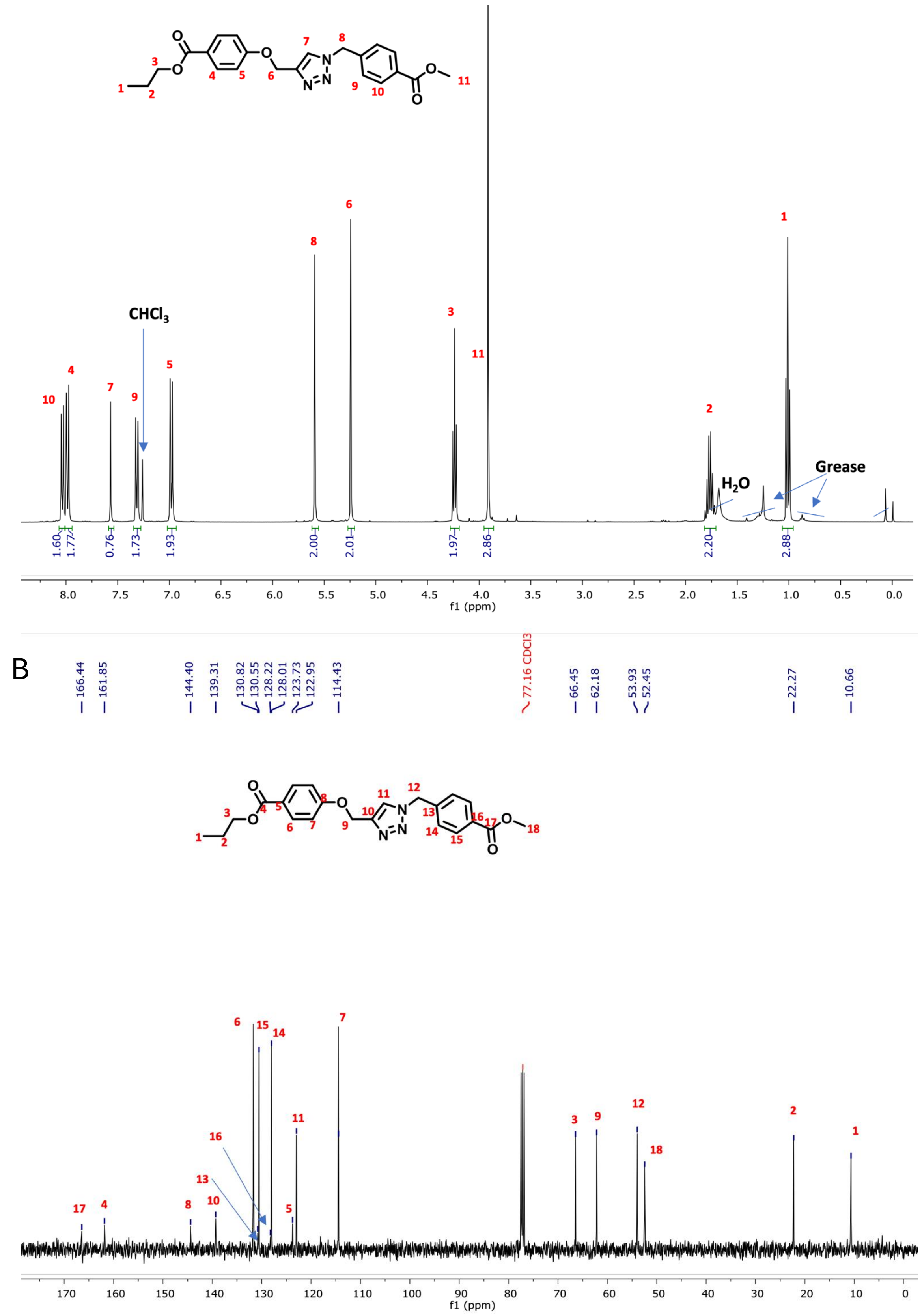

***Figure S17. A**: ^{1}H-NMR spectrum of Product-1 in $CDCl_3$. **B**: ^{13}C-NMR spectrum of Product-1 in $CDCl_3$.*

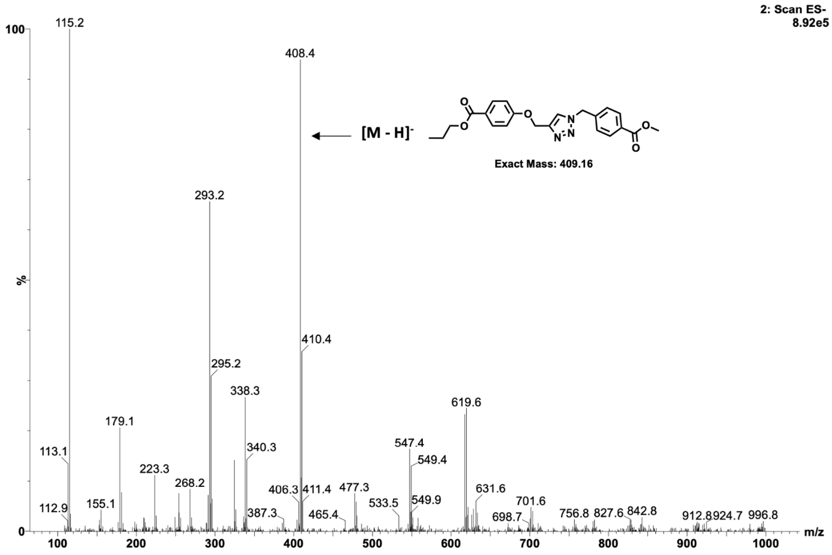

***Figure S18**: MS spectrum of CuAAC product-1.*

Procedure for making product-2:

Ak-2 (40 mg, 1 eq), Az-2 (51.27 mg, 1.2 eq), and NaASC (41.5 mg, 1 eq) were dissolved in a DMF : H_2O mixture (4:1 v/v). The solution was purged with nitrogen for 10 minutes. $CuSO_4 \cdot 5H_2O$ (52.5 mg, dissolved in 1 mL H_2O) was then added under inert conditions, and the mixture was stirred at room temperature for one hour. Following this, the solution was added to 100 mL of saturated NH_4Cl solution, and the crude product was extracted three times with 50 mL of DCM. The combined DCM extracts were dried over $MgSO_4$. DCM fraction was evaporated and the crude product was purified by flash chromatography using a 50:50 v/v mixture of EtOAc : hexanes. The final product (Product-2) was obtained as a white solid in 75 % yield.

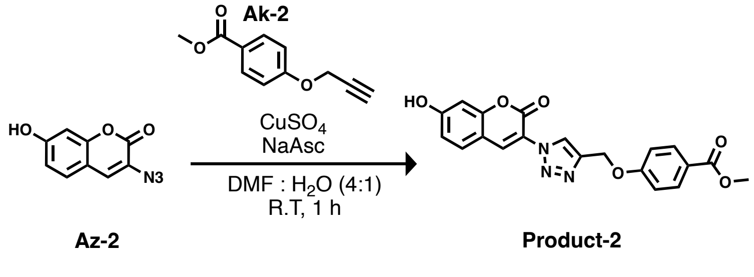

***Scheme S12:** Synthetic route for CuAAC product-2.*

^{1}H NMR (400 MHz, DMSO-d_6) δ: 8.71 (s, 1**H**, -N-C=C-), 8.59 (s, 1**H**, Ar-H), 7.93 (d, *J* = 8.3 Hz, 2**H**, Ar-H), 7.73 (d, *J* = 8.5 Hz, 1**H**, Ar-**H**), 7.20 (d, *J* = 8.2 Hz, 2**H**, Ar-H), 6.88 (d, *J* = 8.5 Hz, 1H, Ar-**H**), 6.82 (s, 1**H**, Ar-H), 5.35 (s, 2**H**, -O-CH_2-), 3.82 (s, 3**H**, -O-C**H**$_3$).

^{13}C NMR (100 MHz, DMSO) δ: 162.54, 161.86, 156.38, 154.71, 142.36, 136.61, 131.27, 131.26, 131.04, 129.62, 125.87, 122.26, 119.29, 114.82, 114.34, 110.35, 102.21, 69.81.

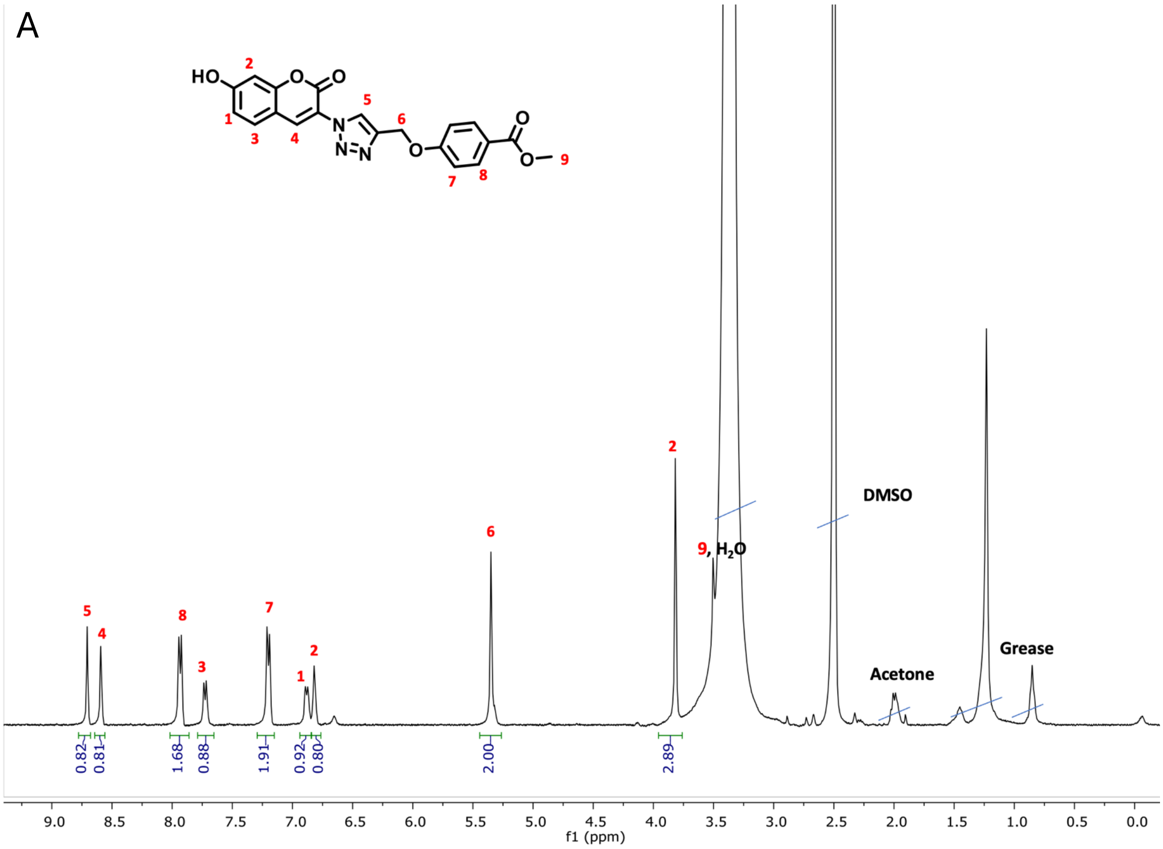

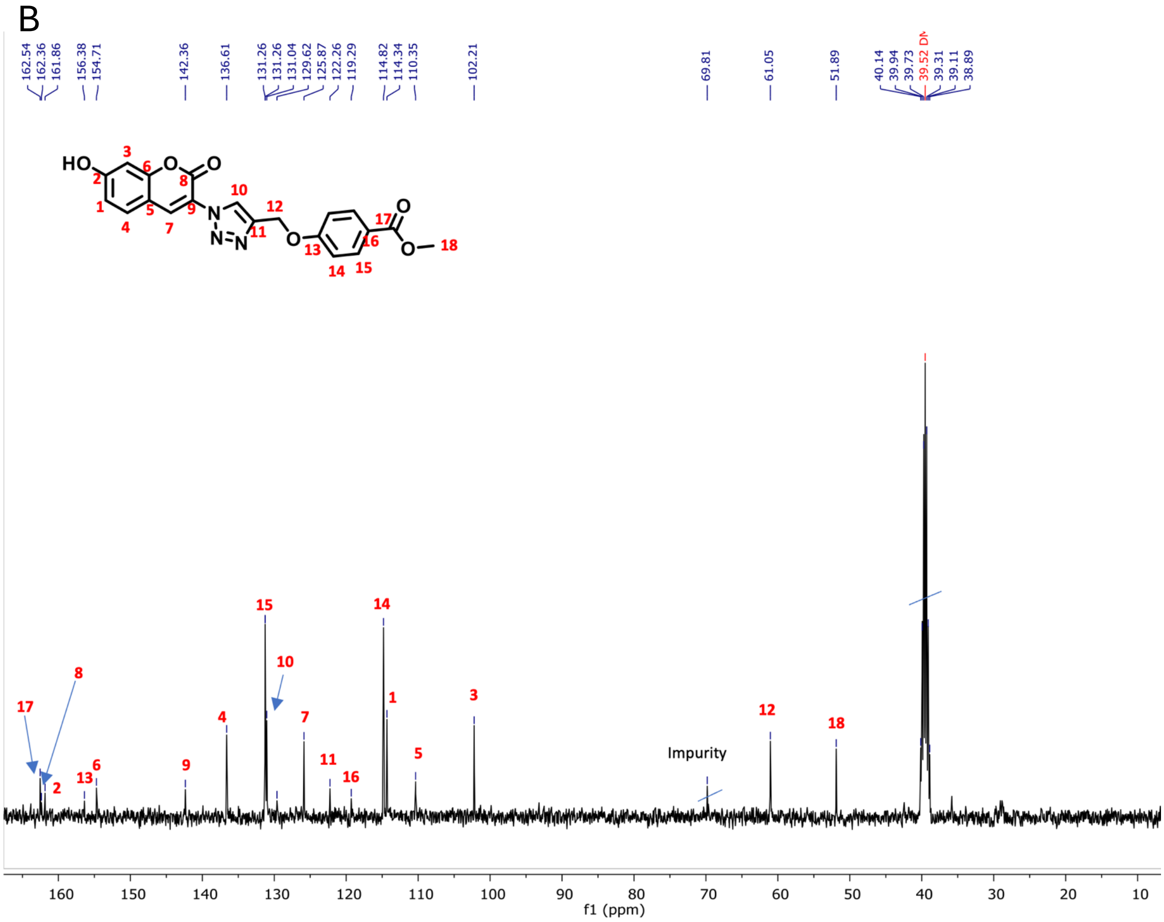

***Figure S19: A**. ^{1}H-NMR spectrum of Product-2 in DMSO. **B.** ^{13}C-NMR spectrum of Product-2 in DMSO.*

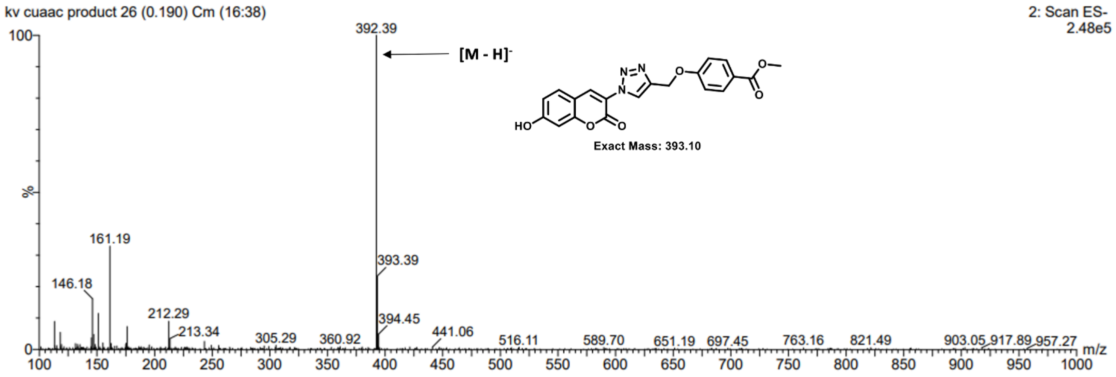

***Figure S20**: MS spectrum of CuAAC product-2.*

Characterization of Amphiphiles

Size exclusion chromatography (SEC)

Instrument: Malvern Viscotek GPCmax

Columns: 2xPSS GRAM 1000Å

Column temperature*:* 50°C

Flow rate: 0.5 mL/min

Injection time: 60 min

Injection volume: 50 μL from a 10 mg/mL sample

Diluent + mobile phase: DMF + 25 mM Ac

Needle wash: DMF

Detector: Viscotek VE3580 RI detector

Sample preparation: The amphiphiles were directly dissolved in the diluent to give a final concentration of 10 mg/mL and filtered with 0.45 μm PTFE syringe filter.

A.

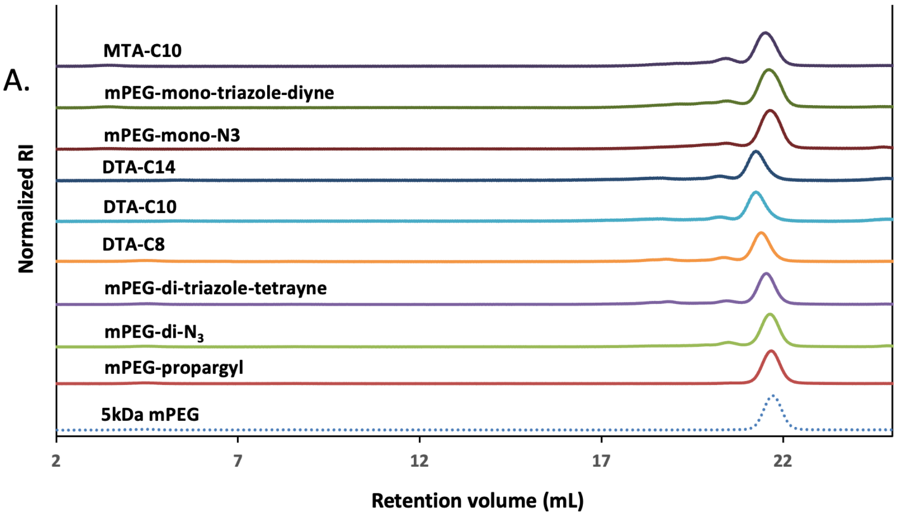

B.

Sample Name	Mn (kDa)	Đ
5kDa mPEG	5,107	1.03
mPEG-propargyl	5,132	1.03
mPEG-di-N3	5,216	1.03
mPEG-di-triazole-diyne	5,866	1.03
DTA-C8	6,139	1.03
DTA-C10	6,702	1.03
DTA-C14	6,718	1.03
mPEG-mono-N3	5,137	1.03
mPEG-mono-triazole-diyne	5,312	1.03
MTA-C10	5,773	1.03

***Figure S21**: **A.** SEC traces overlay polymers and amphiphiles. **B.** Experimental M_n and Dispersity based on PEG standards.*

Infrared spectroscopy (IR)

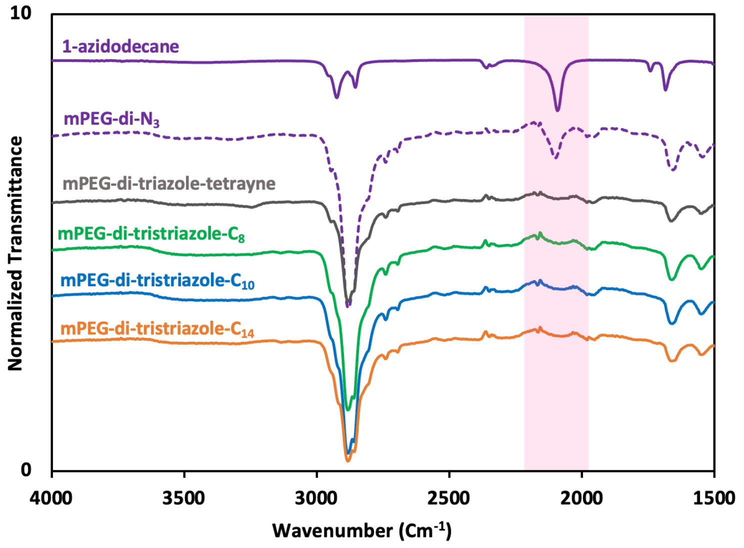

***Figure S22**: IR transmittance spectra overlay of mPEG-di-azide, mPEG-di-triazole-tetrayne, and final DTAs. The range for azide IR absorbance band is highlighted in pink.*

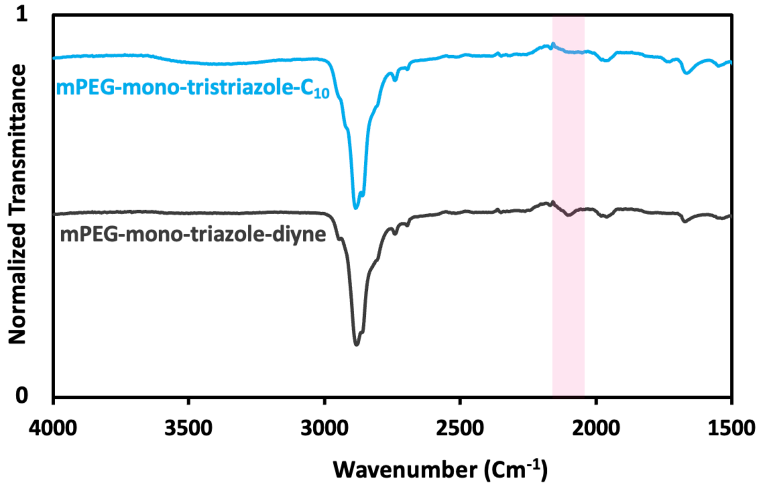

***Figure S23**: IR transmittance spectra overlay of mPEG-mono-triazole-diyne and MTA-C10. The range for azide IR absorbance band is highlighted in pink.*

Characterization of DTA micellar structures

Critical micelle concentration (CMC)

General procedure and measurement:

Preparation of diluent:

Nile Red (NR) stock solution (0.88 mg/mL in ethanol) was diluted into a phosphate buffer saline (pH 7.4) to afford a final concentration of 1.25 µM.

Preparation and measurement of samples:

DTAs were directly dissolved in the diluent to give a final concentration of 500 µM. Solution was vortexed vigorously until the amphiphile completely dissolved and further sonicated for 15 minutes in an ultrasonic bath. The solutions were consecutively diluted by a factor of 1.5 with the diluent to afford a series of 24 samples for each amphiphile. 150 µL of each sample was loaded onto a 96 well plate and a fluorescence emission scan was performed for each well. To determine the hybrid's CMC – the maximum emission of Nile Red (at about 630 nm) was plotted as a function of the amphiphile's concentration. This procedure was repeated thrice for each amphiphile, and mean value is reported as the CMC value and the standard deviation as measurement error.

Instrument method:

Instrument: TECAN Infinite M200Pro

Excitation: 550 nm

Emission intensity scan: 580-800 nm

Step: 2 nm

Number of flashes: 15

Gain: 100

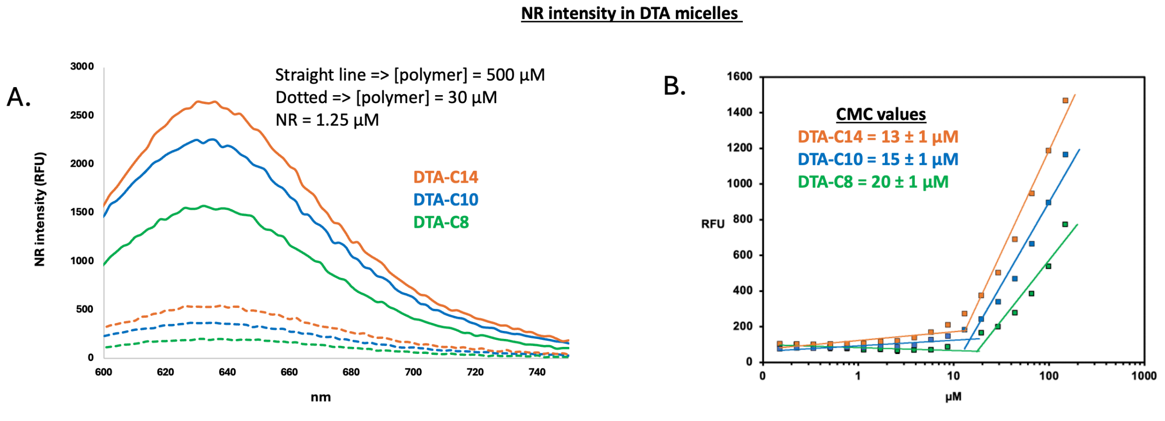

Figure S24: CMC measurements of DTAs. **A.** Fluorescent spectra showing NR intensity at two different DTA concentrations (NR = 1.25 µM). **B.** CMC values calculated by plotting concentration of NR against relative fluorescence units (RFU).

Loading capacity of Pluronic P123 micelles

Pluronic micelles were loaded with 100 µM each of Ak-1 and Az-1, and separately with 100 µM each of Ak-2 and Az-2, by adding the 3µL of respective substrate stock solutions to the Pluronic P123 micellar solution (2 mg/mL in PBS, substrate stock = 33.3 mM in DMSO, final volume = 1 mL, final DSMO concentration is 0.6 %). The vials were briefly vortexed, and the resulting solution was filtered through a 0.22 µm nylon syringe filter to remove any undissolved substrates. The concentration of substrates in the filtrate was then quantified using HPLC.

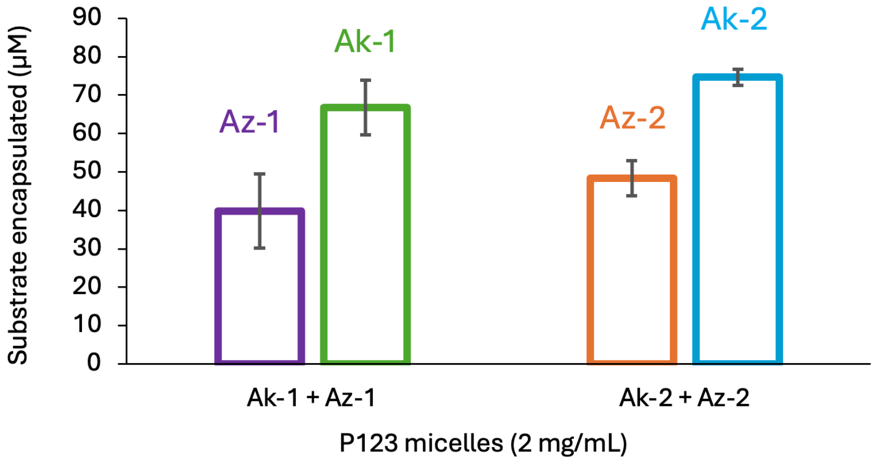

***Figure S25**: Loading capacity of Pluronic P123 micelle in PBS (P123 = 2 mg/mL).*

Dynamic light scattering (DLS)

Sample preparation:

Amphiphile solution (0.2 %w/v) in PBS was filtered through 0.22 µm nylon syringe filter and the micelle size was analyzed. For analysis of Cu loaded MNR's size, 5 µL of $CuSO_4$ (stock solution in H_2O is 2 mM, final concentration = 10 µM) was added to the filtered micelle solution (1 mL, PBS) and vortexed for a min.

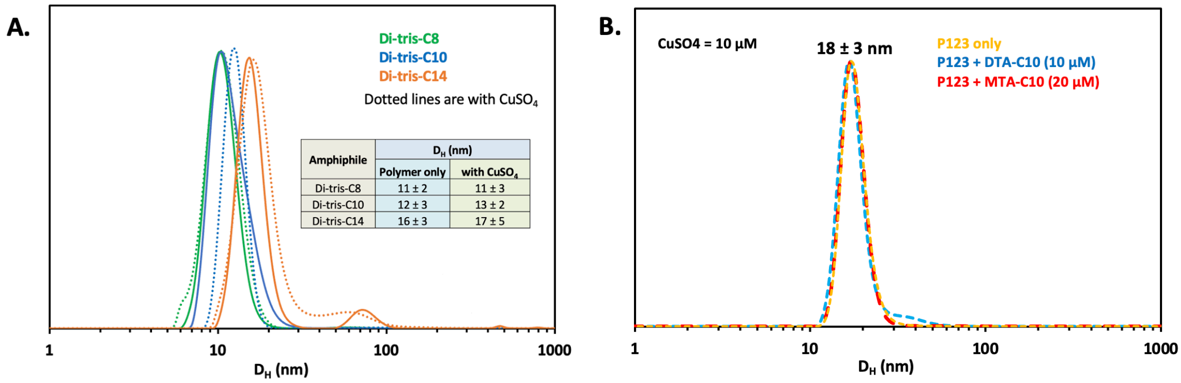

***Figure S26**: **A.** DLS measurements overlay of DTAs (bold line) and in presence of $CuSO_4$ (10 µM, dashed line). **B.** DLS measurements of Cu-MNRs made from P123 only (dotted yellow line), and mixed micelles made with P123 (2 mg/mL) and DTA-C10 (10 µM) showed in dotted blue line, and mixed micelles made with P123 (2 mg/mL) and MTA-C10 (20 µM) showed in dotted red line.*

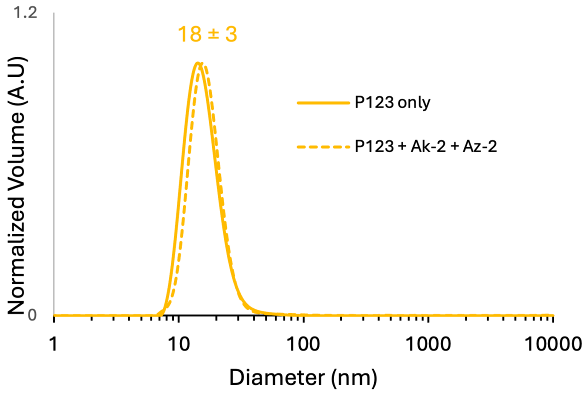

***Figure S27**: DLS measurements overlay of pure Pluronic P123 micelles before and after loading with 40 µM of Ak-2 and 40 µM of Az-2 (polymer = 2 mg/mL in PBS).*

Kinetic measurements

HPLC measurements

Instrument: Waters Alliance e2695

Column: Waters XBridge Protein BEH C4 Column, 300Å, 3.5 µm, 4.6 mm X 150 mm

Column temperature: 25°C

Sample temperature: 37°C

Solution A: 0.1% $HClO_4$ in MilliQ H_2O : ACN = 95 : 5 v/v

Solution B: 0.1% $HClO_4$ in MilliQ H_2O : ACN = 5 : 95 v/v

Solution C: MeCN

Flow rate: 1 mL/min

Injection volume: 10 µL

Seal wash: H_2O : MeOH = 90 : 10 v/v

Needle wash: MeOH

Detector: Waters 2998 photodiode array detector

Sampling rate: 2 points/sec

Gradient program for 15 minutes method:

Time [min]	Solution A [%]	Solution B [%]	Solution C [%]
0.00	80	15	5
1.00	80	15	5
7.00	0	95	5
10.00	0	95	5
10.10	80	15	5
15.00	80	15	5

Gradient program for 10 minutes method:

Time [min]	Solution A [%]	Solution B [%]	Solution C [%]
0.00	70	30	0
1.00	70	30	0
7.00	0	95	5
9.00	0	95	5
9.10	70	30	0
10.00	70	30	0

Stability of azide-2

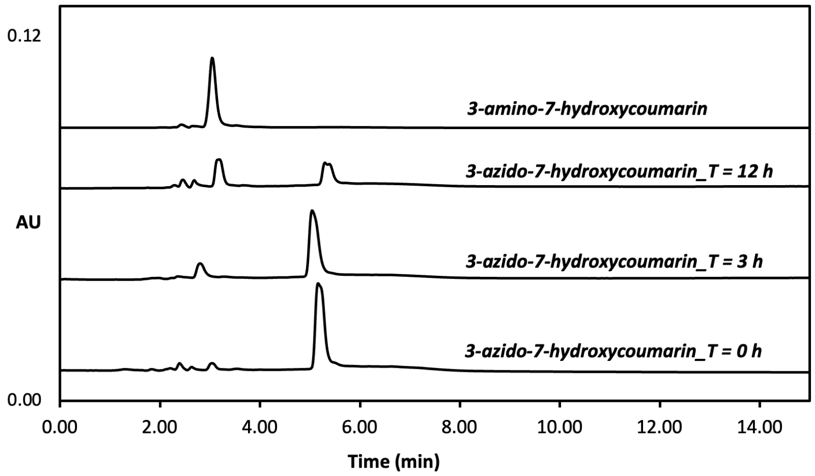

Figure S28: *Representative HPLC chromatogram overlay (taken at 340 nm), showing, the reduction of 3-azido-7-hydroxycoumarin to 3-amino-7-hydroxycoumarin in H_2O : MeCN (1 : 1 v/v) at 37°C.*

Reaction progress of product-1 monitored through HPLC

General procedure for setting up CuAAC in micelles.

Micelles were made by directly dissolving amphiphiles in PBS at pH 7.4 resulting in a final solution containing 0.2 % w/v of amphiphiles (1 mL). Then the 3.5 µL of Ak-1 and Az-1 (substrate stock solution in DMSO, 11.43 mM each, final concentrations of Ak-2 and Az-2 are 40 ± 5 µM each) were added to the micellar solution, along with 5 µL of NaASC (stock solution in H_2O is 400 mM, final concentration = 2 mM) and the solutions were briefly vortexed (final concentration of DMSO is less than one percent). Initial concentration of substrates was determined by HPLC. Finally, to initiate the CuAAC rection, 5 µL of $CuSO_4$ (stock solution in H_2O is 2 mM, final concentration = 10 µM) was added to the micellar solution and the reaction progress was monitored by re-injecting the sample into HPLC at predetermined time points. The experiments were conducted at 37°C.

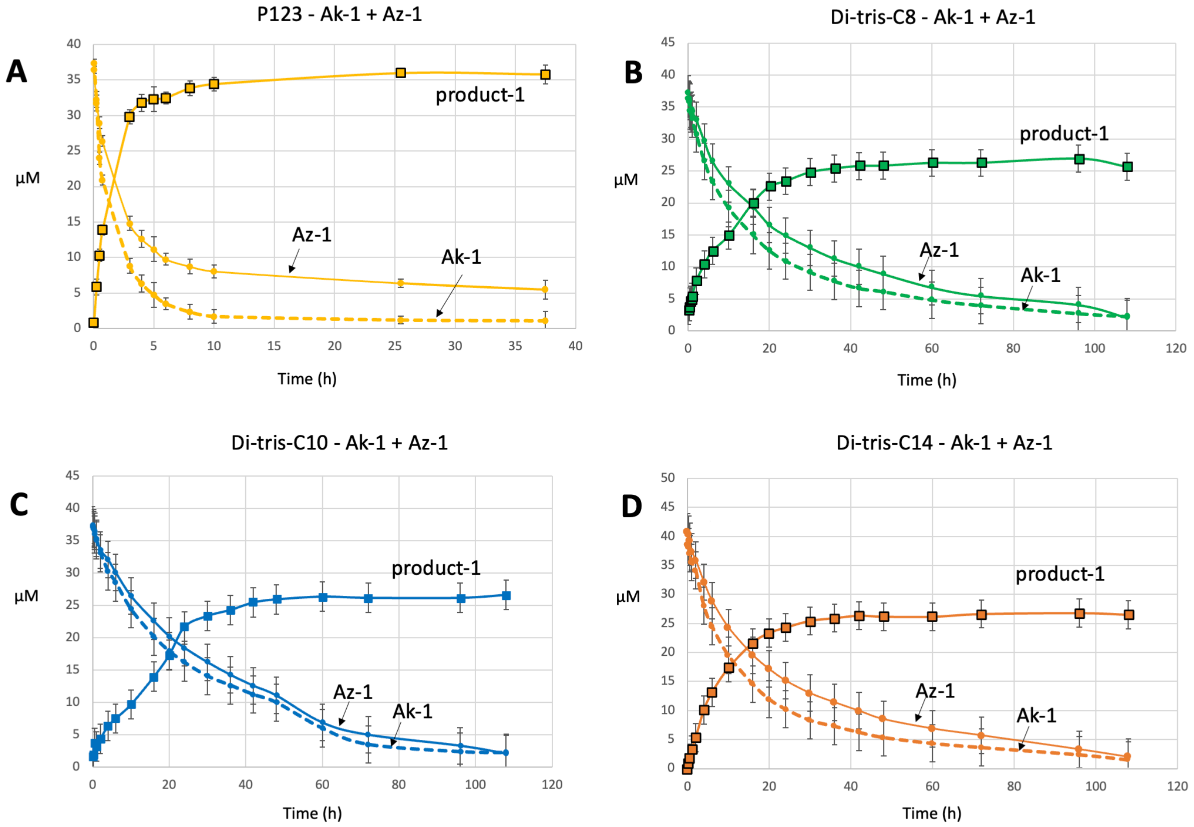

***Figure S29**: Ak-1 and Az-1 consumption and Product-1 formation with Cu-MNRs monitored through HPLC. **A.** Micelles made with P123 only, **B**, **C** and **D** represent micelles made from DTA-C8, DTA-C10 and DTA-C14 amphiphiles respectively. Amphiphile = 2 mg/mL, [Alkyne-2] = 40 ± 5 µM, [Azide-2] = 40 ± 5 µM, [NaASC] = 2 mM, [$CuSO_4$] = 10 µM. **B**, **C** and **D** has [tris-triazole] to [Cu] in a ratio of 65 : 1.*

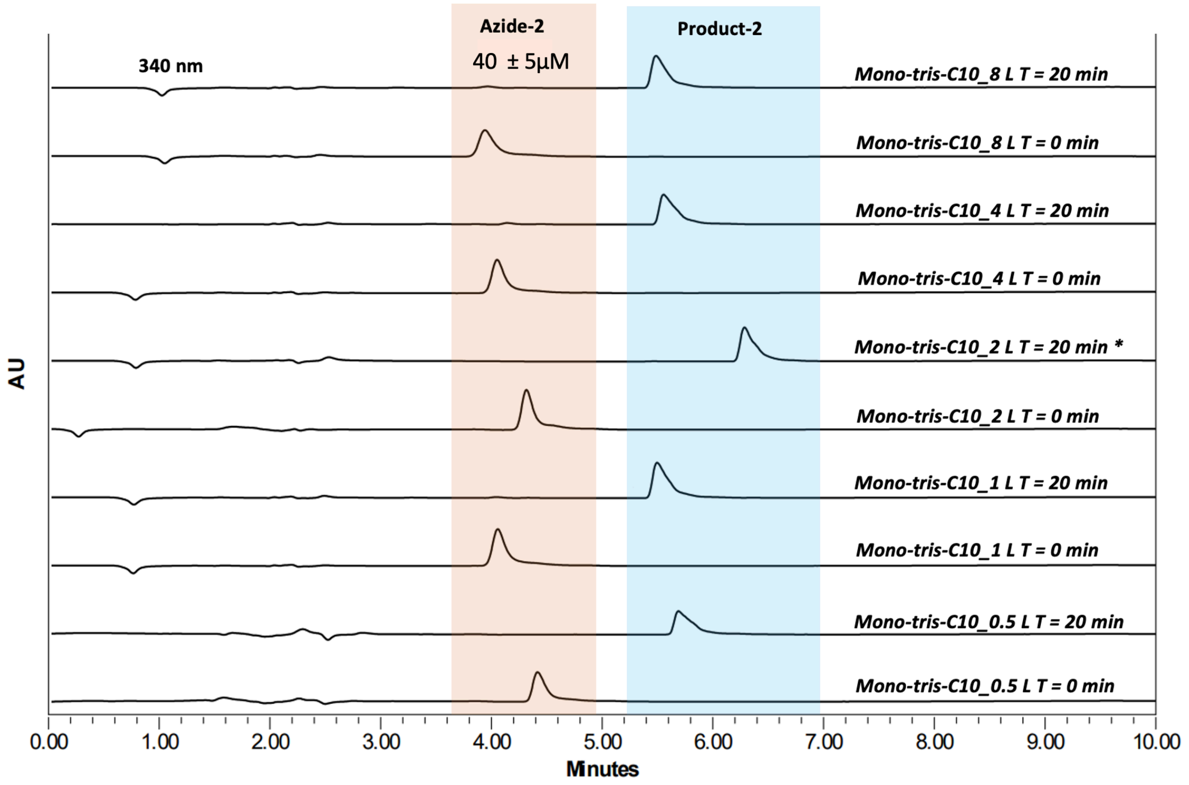

***Figure S30**: Representative HPLC chromatogram overlay (taken at 340 nm), showing, the transformation of azide-2 to product-2 with Cu-MNRs made from mixing P123 and varying amount of MTA-C10 amphiphiles. The final ratio of tris-triazole ligands for per one [Cu] ion are shown as 0.5 L, 1 L, 2 L, 4 L and 8 L. T = 0 is the chromatogram taken before adding $CuSO_4$ and T = 20 min after observing the florescence intensity plateau with fluorometer. Amphiphile (P123 + MTA-C10) = 2 mg/mL, [Alkyne-2] = 40 ± 5 µM, [Azide-2] = 40 ± 5 µM, [NaASC] = 2 mM, [$CuSO_4$] = 10 µM. The chromatogram with asterisk mark was obtained with 15 min HPLC method while the rest are obtained with 10 min HPLC method.*

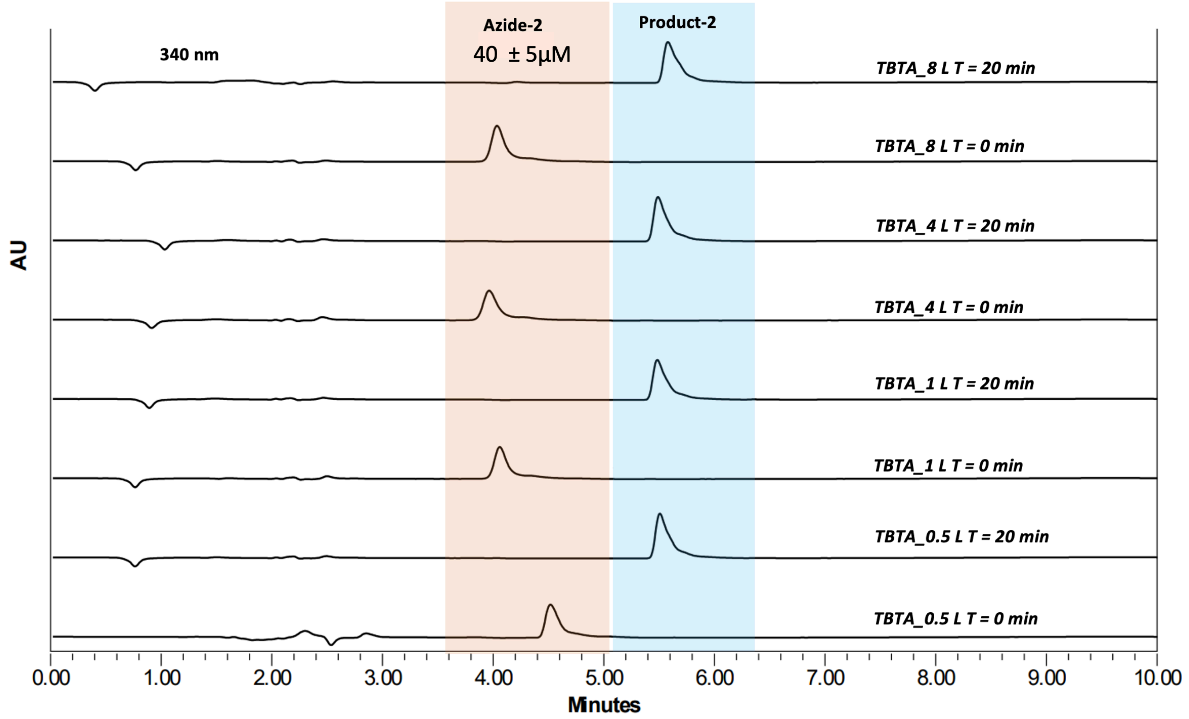

***Figure S31**: Representative HPLC chromatogram overlay (taken at 340 nm), showing, the transformation of azide-2 to product-2 with Cu-MNRS made by encapsulating P123 and varying amount TBTA. The final ratio of tristriazole ligands for per one [Cu] ion are shown as 0.5 L, 1 L, 4L, and 8 L. T = 0 is the chromatogram taken before adding $CuSO_4$ and T = 20 min after observing the florescence intensity plateau with fluorometer. Amphiphile = 2 mg/mL, [Alkyne-2] = 40 ± 5 µM, [Azide-2] = 40 ± 5 µM, [NaASC] = 2 mM, [$CuSO_4$] = 10 µM. The chromatograms are obtained with 10 min HPLC method.*

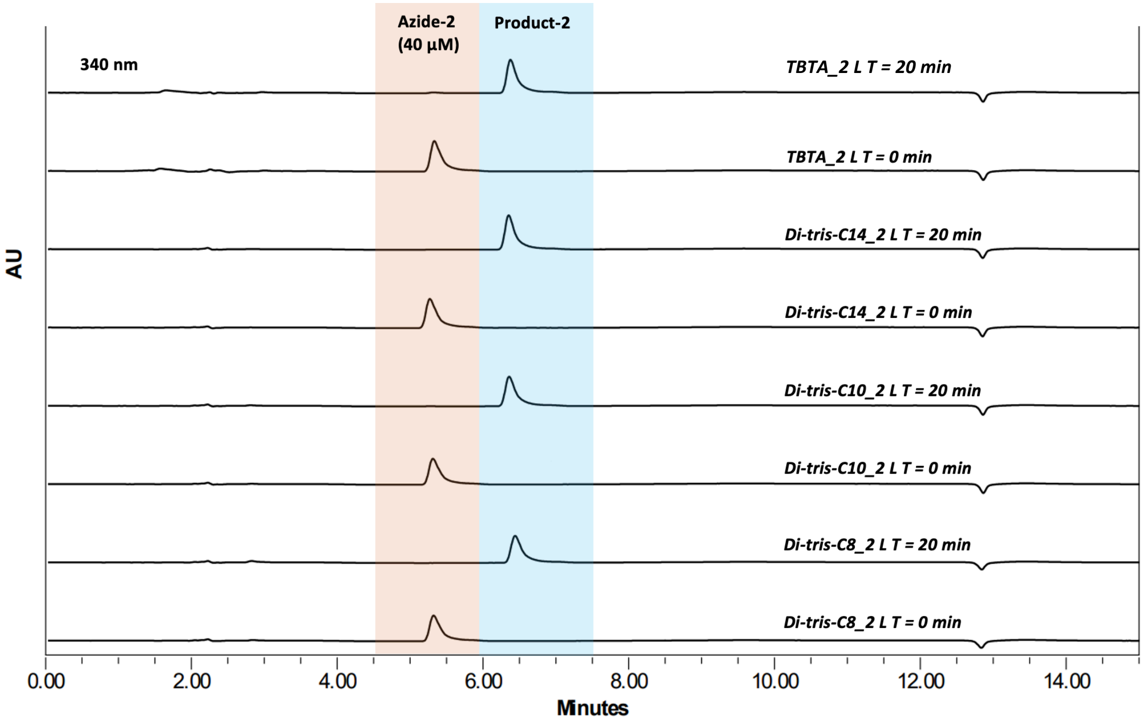

***Figure S32**: Representative HPLC chromatogram overlay (taken at 340 nm), showing, the transformation of azide-2 to product-2 with Cu-MNRS made from mixing P123 and varying amount of DTA amphiphiles and TBTA. (ratio of tristriazole ligands to Cu is 2:1). T = 0 chromatogram was taken before adding $CuSO_4$ and T = 20 min is after observing the florescence intensity plateau with fluorometer. Amphiphile = 2 mg/mL, [Alkyne-2] = 40 ± 5 µM, [Azide-2] = 40 ± 5 µM, [NaASC] = 2 mM, [$CuSO_4$] = 10 µM. The chromatograms are obtained with 15 min HPLC method.*

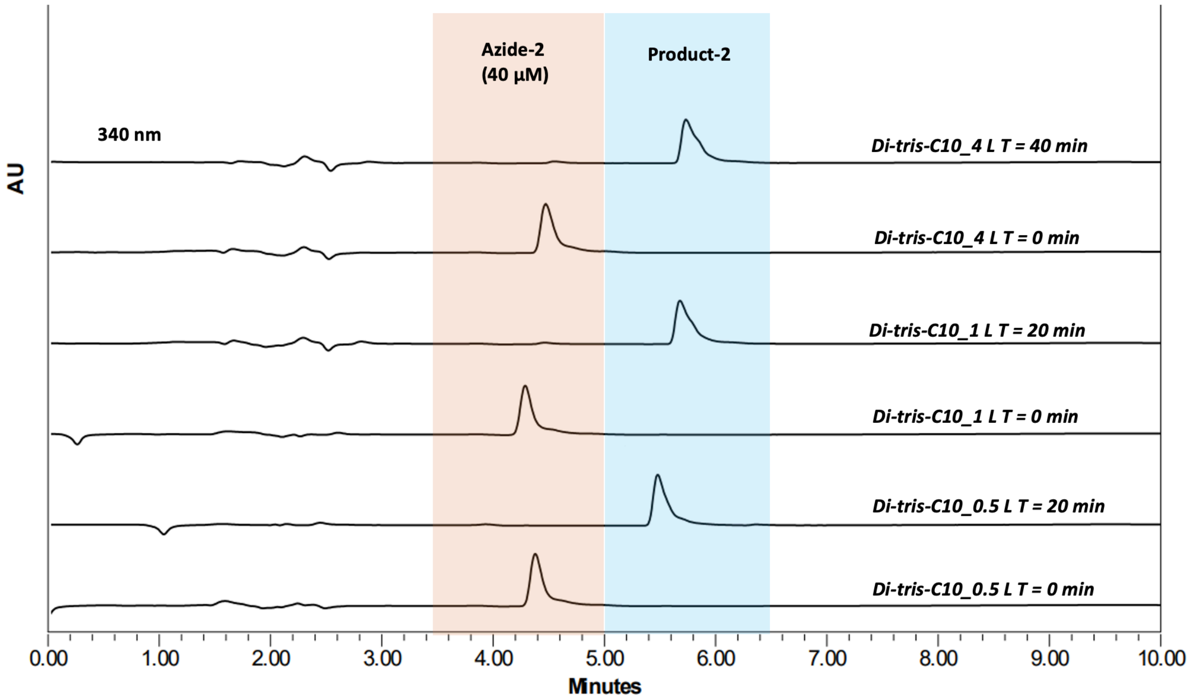

Figure S33*: Representative HPLC chromatogram overlay (taken at 340 nm), showing, the transformation of azide-2 to product-2 with Cu-MNRS made from mixing P123 and varying amount of DTA-C10 amphiphiles. The final ratio of tristriazole ligands for per one [Cu] ion are shown as 0.5 L, 1 L, and 4 L. T = 0 is the chromatogram taken before adding $CuSO_4$ and T = 20 min (for ratio of tristriazole ligands to Cu is 0.5:1 and 1:1), and T = 40 min (for ratio of tristriazole ligands to Cu is 4:1), after observing the florescence intensity plateau with fluorometer. Amphiphile = 2 mg/mL, [Alkyne-2] = 40 ± 5 µM, [Azide-2] = 40 ± 5 µM, [NaASC] = 2 mM, [$CuSO_4$] = 10 µM. The chromatograms are obtained with 10 min HPLC method.*

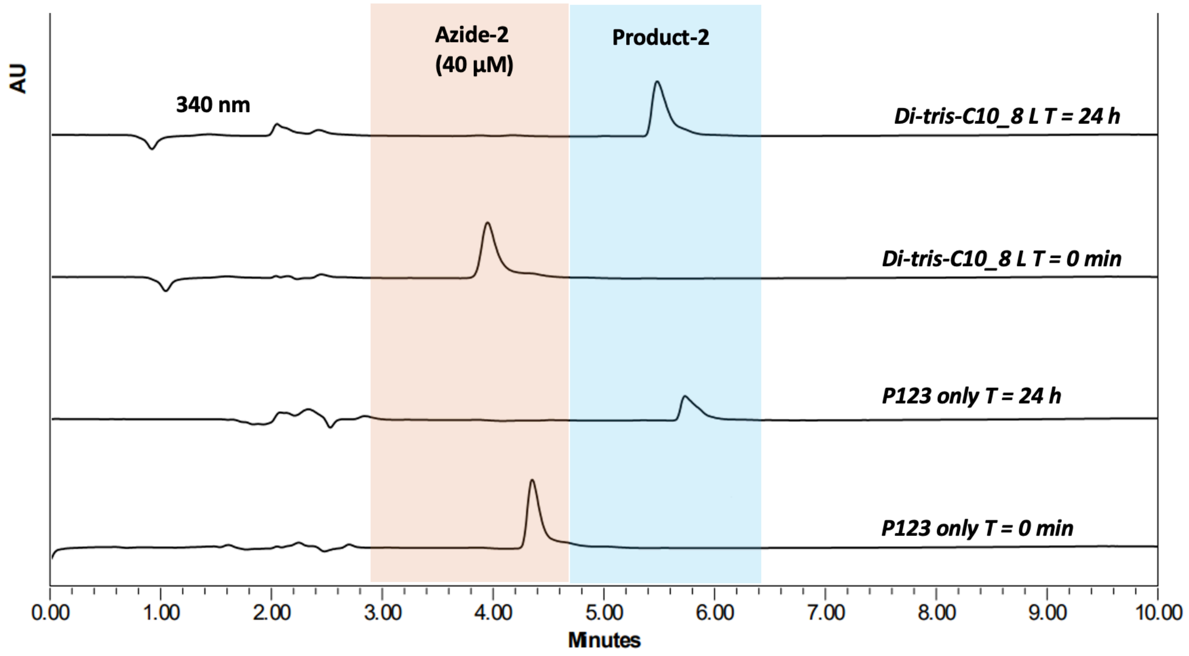

Figure S34: *Representative HPLC chromatogram overlay (taken at 340 nm), showing, the transformation of azide-2 to product-2 with Cu-MNRS made from P123 amphiphiles only, and by mixing P123 and DTA-C10 amphiphiles (ratio of tristriazole ligands to Cu is 8:1). T = 0 chromatogram was taken before adding $CuSO_4$ and T = 24 h is after observing the florescence intensity plateau with fluorometer. The sample was diluted by a factor of 2 with MeCN and injection volume was doubled. Amphiphile = 2 mg/mL, [Alkyne-2] = 40 ± 5 µM, [Azide-2] = 40 ± 5 µM, [NaASC] = 2 mM, [$CuSO_4$] = 10 µM. The chromatograms are obtained with 10 min HPLC method.*

Fluorescence measurements and Reaction rates

General procedure for setting up CuAAC in micelles.

Micelles were made by directly dissolving amphiphiles in PBS, pH 7.4 (either p123 only or by mixing p123 and DTA/MTA) such that the final solution has total of 0.2 % w/v of amphiphiles. 2 mL samples were aliquoted and 7 µL of Ak-2 and Az-2 (substrate stock solution in DMSO, 11.43 mM each, final concentrations of Ak-2 and Az-2 are 40 ± 5 µM each) were added to the micellar solution, along with 10 µL NaASC (stock solution in H_2O is 400 mM, final concentration = 2 mM). The final concentration of DMSO is less than one percent. The solutions were briefly vortexed, and the concentration of substrates was quantified by HPLC. Simultaneously, the same solution was used to measure the florescence of the product at (T = 0) using a fluorometer (λ_{exc} = 370 nm, λ_{em} = 475 nm). Finally, into initiate the CuAAC reaction, 3.5 µL of $CuSO_4$ (stock dissolved in H_2O is 2 mM, final concentration = 10 µM, final volume = 700 µL) was added to the micellar solution and the fluorescence spectra was obtained at predetermined time points. Note: All stock solutions ($CuSO_4$, substrate, and polymeric ligand) were sonicated for 5 - 10 minutes before using them.

Fluorescence spectra

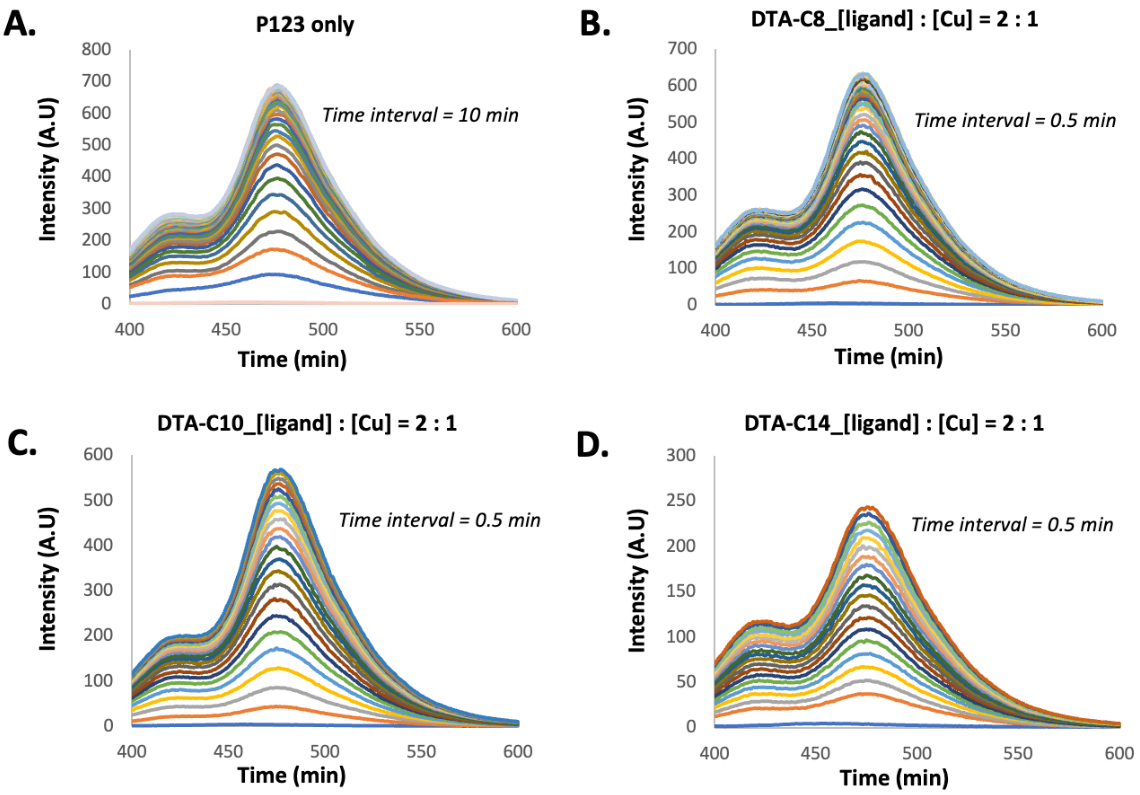

Figure S35: *Fluorescence spectra showing Product-2 formation with Cu-MNRs* ***A.*** *P123 micelles* ***B***. *P123 micelles with 10 µM DTA-C8.* ***C***. *P123 micelles with 10 µM DTA-C10.* ***D.*** *P123 micelles with 10 µM DTA-C14. For all experiments, [net Amphiphile] = 2 mg/mL, [Alkyne-2] = 40 ± 5 µM, [Azide-2] = 40 ± 5 µM, [NaASC] = 2 mM, [$CuSO_4$] = 10 µM. Reactions were conducted at 37°C.*

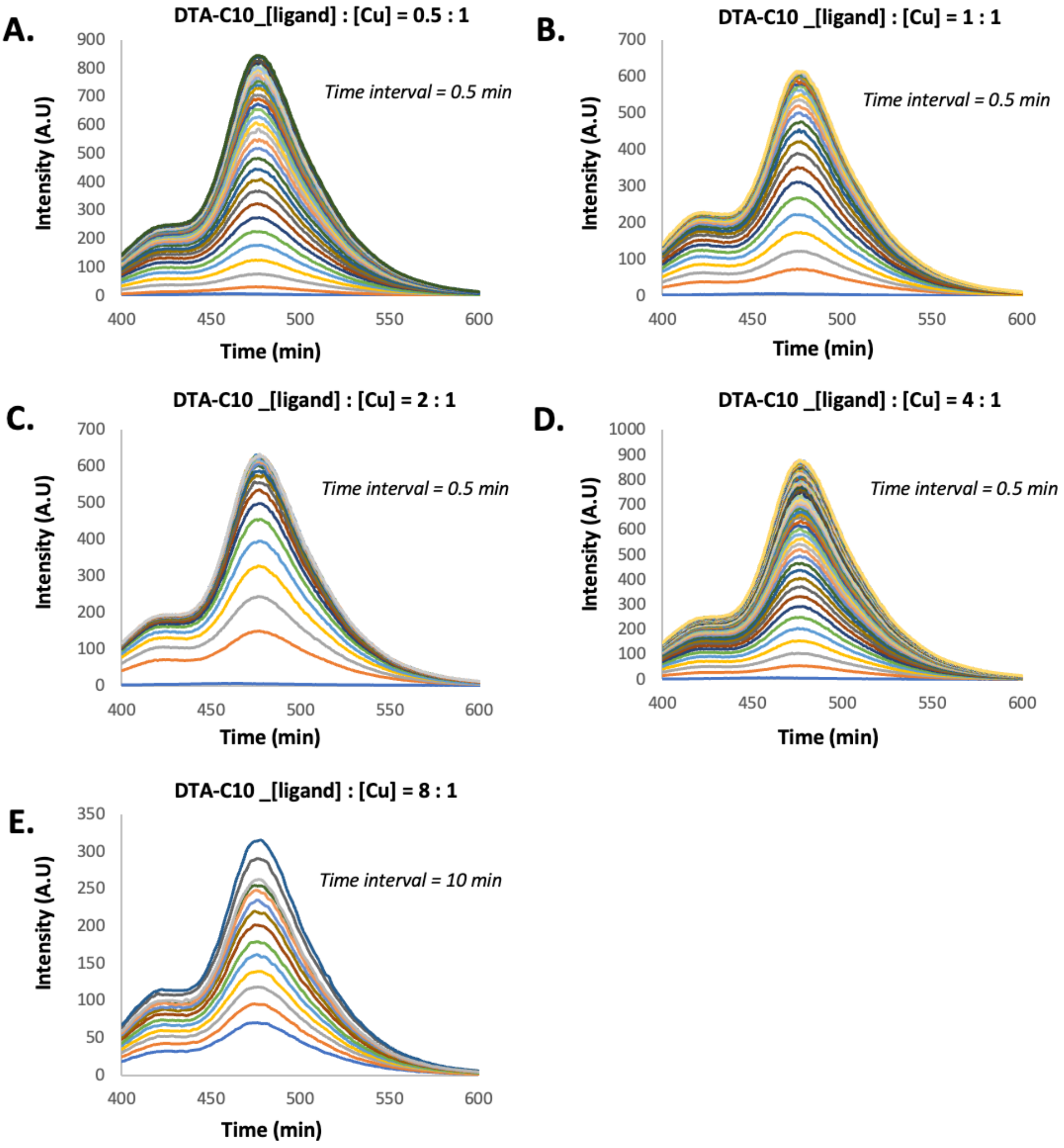

***Figure S36**: Fluorescence spectra showing Product-2 formation with P123 and DTA-C10 mixed micelles loaded with Cu **A**. P123 micelles with 2.5 µM DTA-C10. **B**. P123 micelles with 5 µM DTA-C10. **C.** P123 micelles with 10 µM DTA-C10. **D.** P123 micelles with 20 µM DTA-C10. **E.** P123 micelles with 40 µM DTA-C10. For all experiments, [net Amphiphile] = 2 mg/mL, [Alkyne-2] = 40 ± 5 µM, [Azide-2] = 40 ± 5 µM, [NaASC] = 2 mM, [$CuSO_4$] = 10 µM. Reactions were conducted at 37°C.*

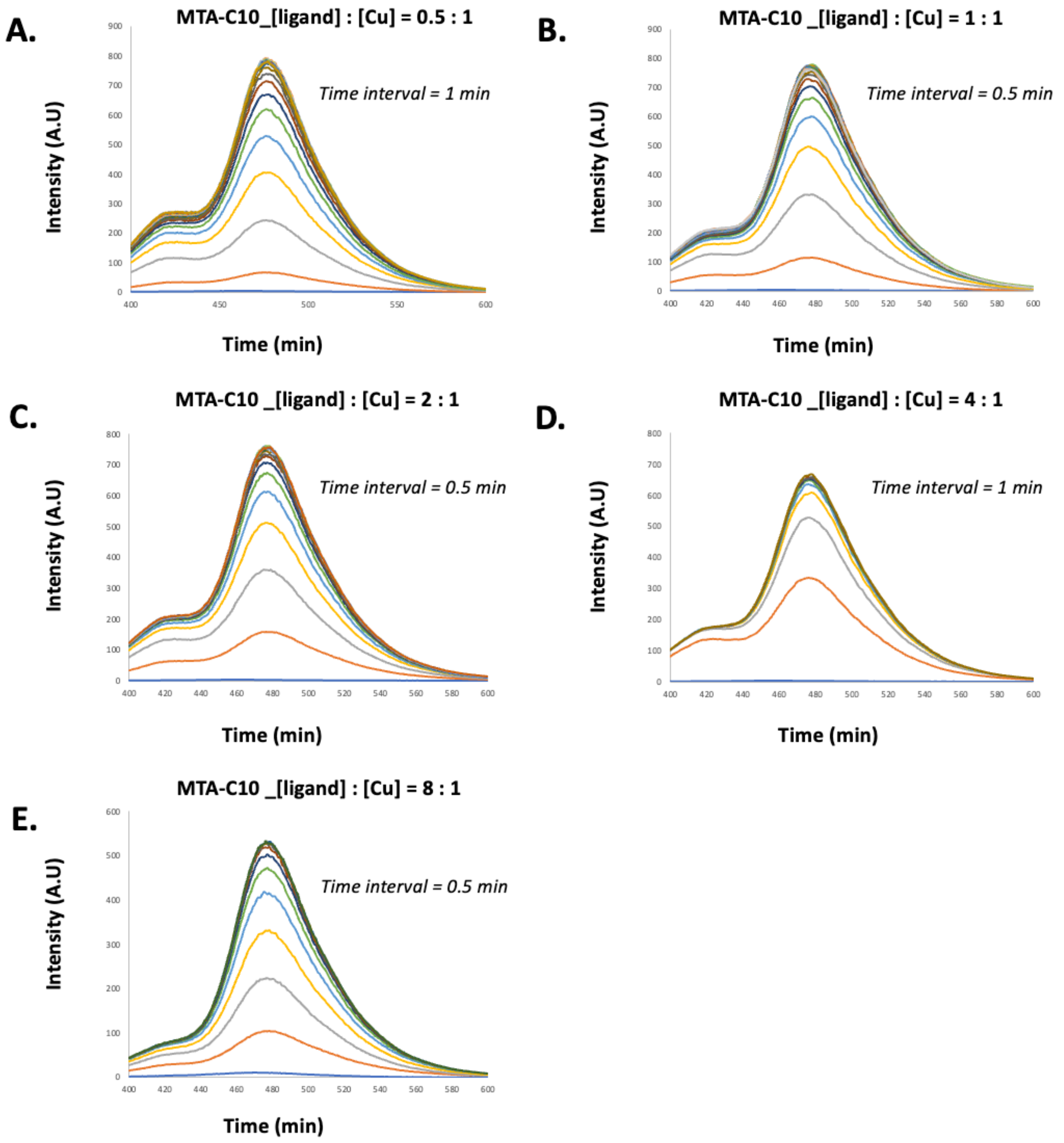

***Figure S37**: **A.** Fluorescence spectra showing Product-2 formation with P123 and MTA-C10 mixed micelles loaded with Cu **A**. P123 micelles with 5 µM MTA-C10. **B**. P123 micelles with 10 µM MTA-C10. **C.** P123 micelles with 20 µM MTA-C10. **D.** P123 micelles with 40 µM MTA-C10. **E.** P123 micelles with 80 µM MTA-C10. For all experiments, [net Amphiphile] = 2 mg/mL, [Alkyne-2] = 40 ± 5 µM, [Azide-2] = 40 ± 5 µM, [NaASC] = 2 mM, [$CuSO_4$] = 10 µM. Reactions were conducted at 37°C.*

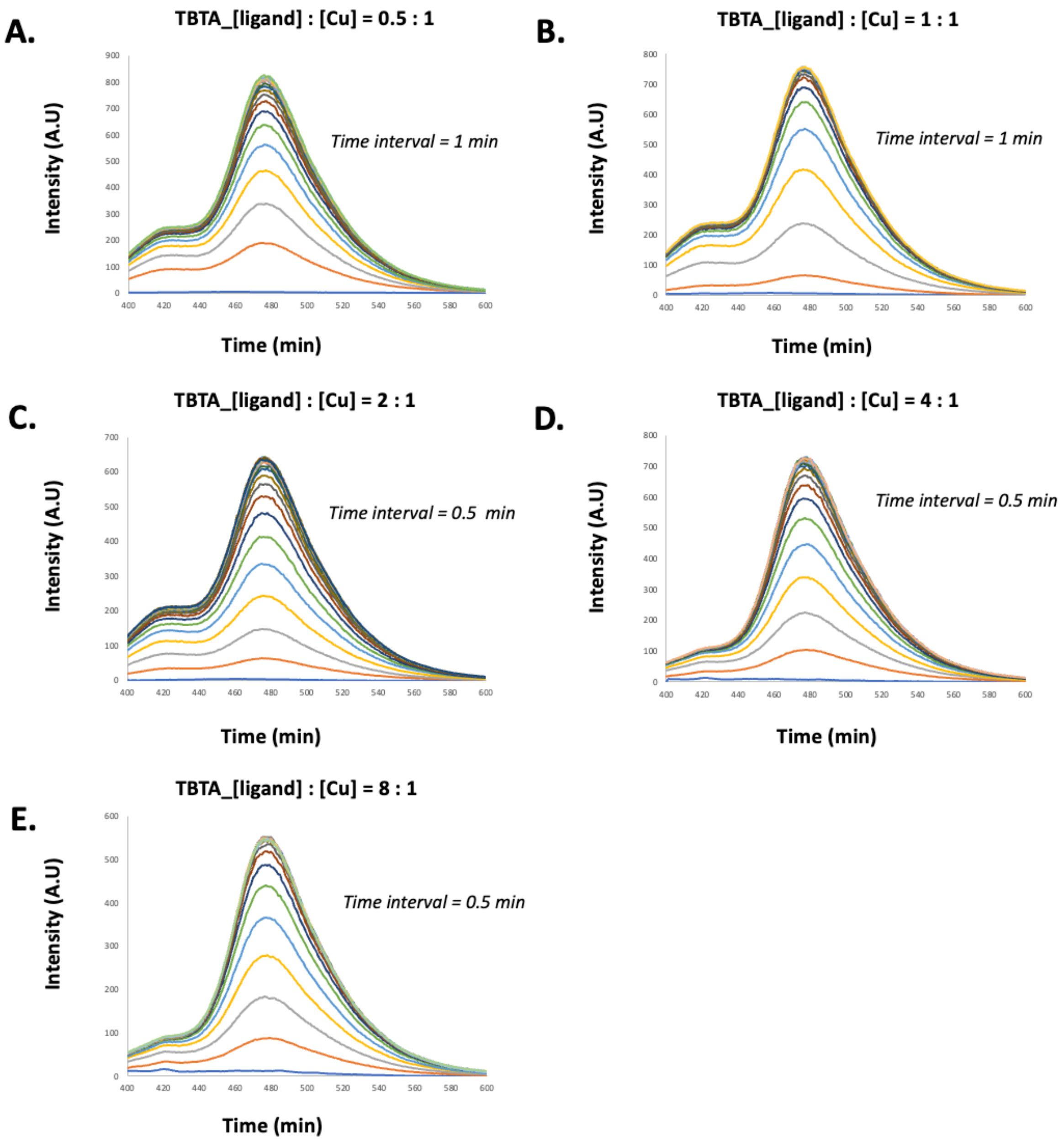

Figure S38: *Fluorescence spectra showing Product-2 formation with P123 micelles encapsulated with TBTA.* ***A****. P123 micelles with 5 µM TBTA.* ***B****. P123 micelles with 10 µM TBTA.* ***C****. P123 micelles with 20 µM TBTA.* ***D****. P123 micelles with 40 µM TBTA.* ***E****. P123 micelles with 80 µM TBTA. For all experiments, Amphiphile = 2 mg/mL, [Alkyne-2] = 40 ± 5 µM, [Azide-2] = 40 ± 5 µM, [NaASC] = 2 mM, [$CuSO_4$] = 10 µM. Reactions were conducted at 37°C.*

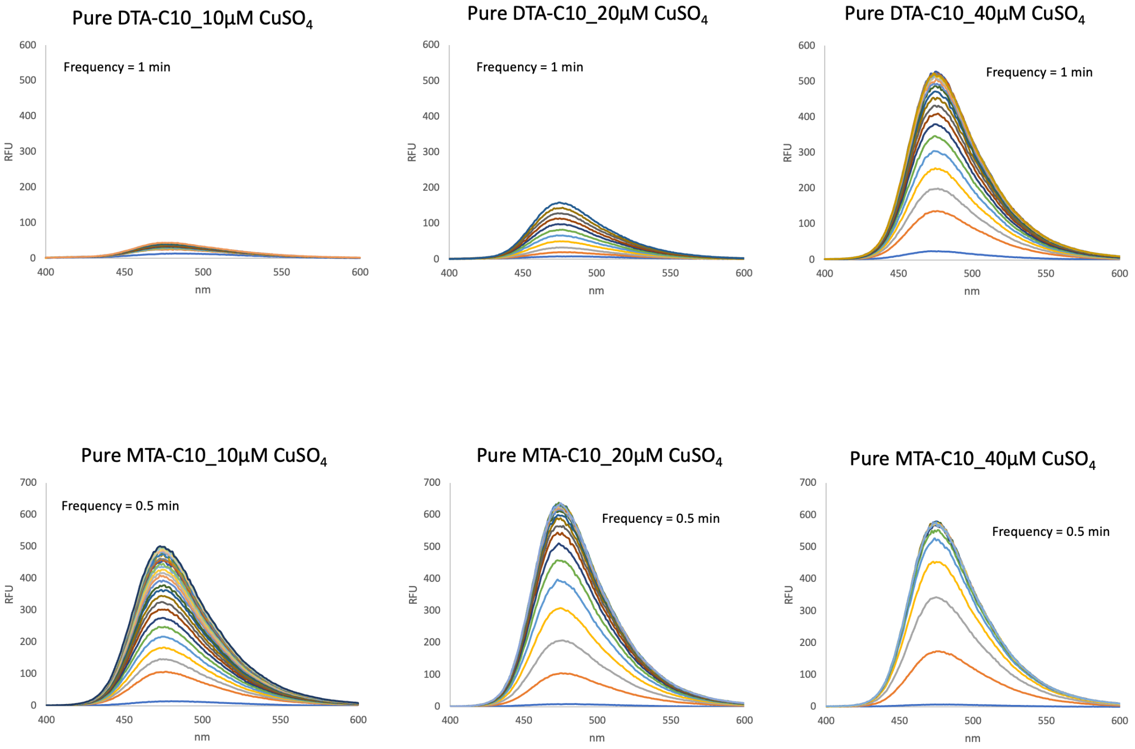

Figure S39: *Fluorescence spectra showing Product-2 formation with pure DTA-C10 and MTA-C10 micelles at different concentrations of [Cu]. For all experiments, amphiphile = 2 mg/mL, [Alkyne-2] = 40 ± 5 µM, [Azide-2] = 40 ± 5 µM, [NaASC] = 2 mM. All reactions were conducted at 37°C.*

Product-2 formation with Cu-MNRs made from P123 only

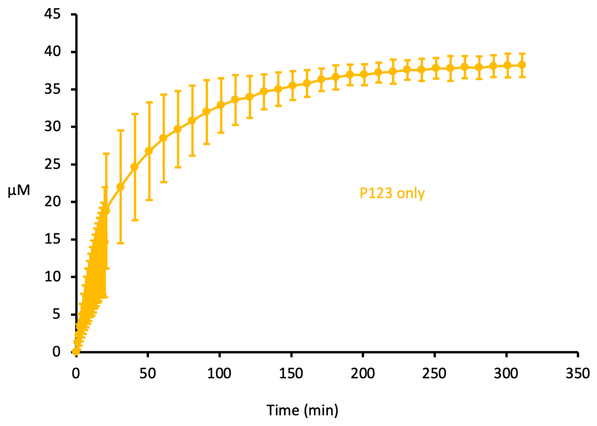

Figure S40*: Product-2 formation with P123 micelles loaded with Cu. Amphiphile = 2 mg/mL, [Alkyne-2] = 40 ± 5 µM, [Azide-2] = 40 ± 5 µM, [NaASC] = 2 mM, [$CuSO_4$] = 10 µM.*

Product-2 formation with Cu-MNRs from DTA-C10 and MTA-C10 at varying [Cu] Levels

The concentration of product-2 was calculated based on the calibration curve of product-2 in pure-DTA-C10, and MTA-C10 (2 mg/mL) (measured from a fluorometer).

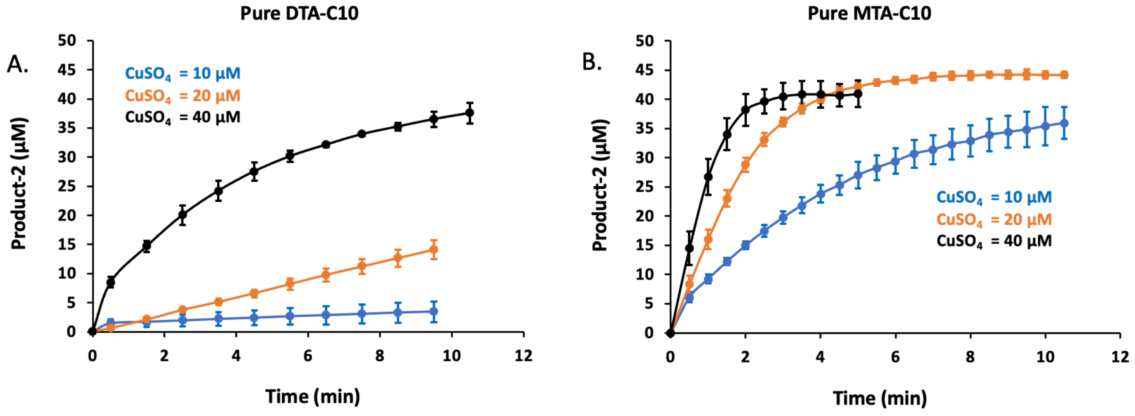

Figure S41: *Product-2 formation with pure micelles loaded with different [Cu]. Amphiphile = 2 mg/mL, [Alkyne-2] = 40 ± 5 μM, [Azide-2] = 40 ± 5 μM, [NaASC] = 2 mM.* ***A****. Pure DTA-C10, and* ***B****. Pure MTA-C10.*

Azide-2 consumption with Pluronic micelles spiked with DTA-C10, MTA-C10 and TBTA

The consumption of azide-2 over time was calculated by subtracting the product-2 formation rate (measured from a fluorometer) from the initial azide-2 concentration (measured from HPLC). This azide-2 consumption data was used to calculate first-order reaction rates.

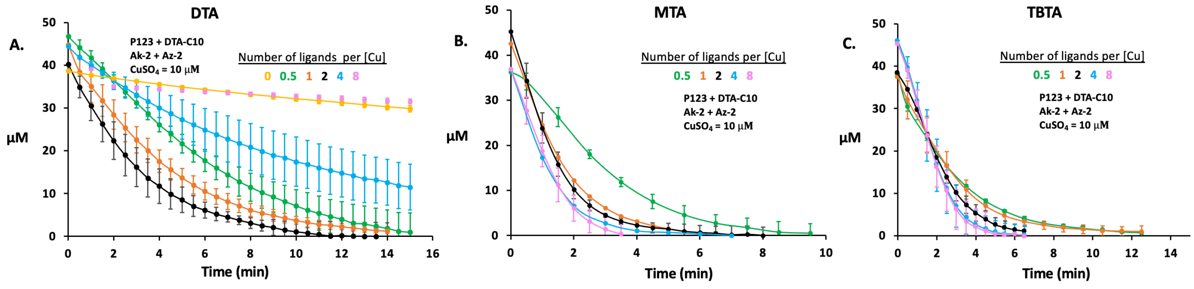

Figure S42: *Azide-2 consumption rate with P123 micelles spiked with different amounts of* ***A.*** *DTA-C10,* ***B.*** *MTA-C10, and* ***C.*** *TBTA. Amphiphile = 2 mg/mL, [Alkyne-2] = 40 ± 5 μM, [Azide-2] = 40 ± 5 μM, [NaASC] = 2 mM, [$CuSO_4$] = 10 μM.*

Azide-2 consumption with Cu-MNRs from DTA-C10 and MTA-C10 at varying [Cu] Levels

The consumption of azide-2 over time was determined by subtracting the rate of product-2 formation, as measured by a fluorometer, at each time point from its final concentration. For pure DTA-C10 micelles, when [Cu] = 10 µM and 20 µM, the consumption of azide-2 over time was calculated based on the initial azide-2 concentration measured by HPLC. This azide-2 consumption data was then used to calculate the first-order reaction rates.

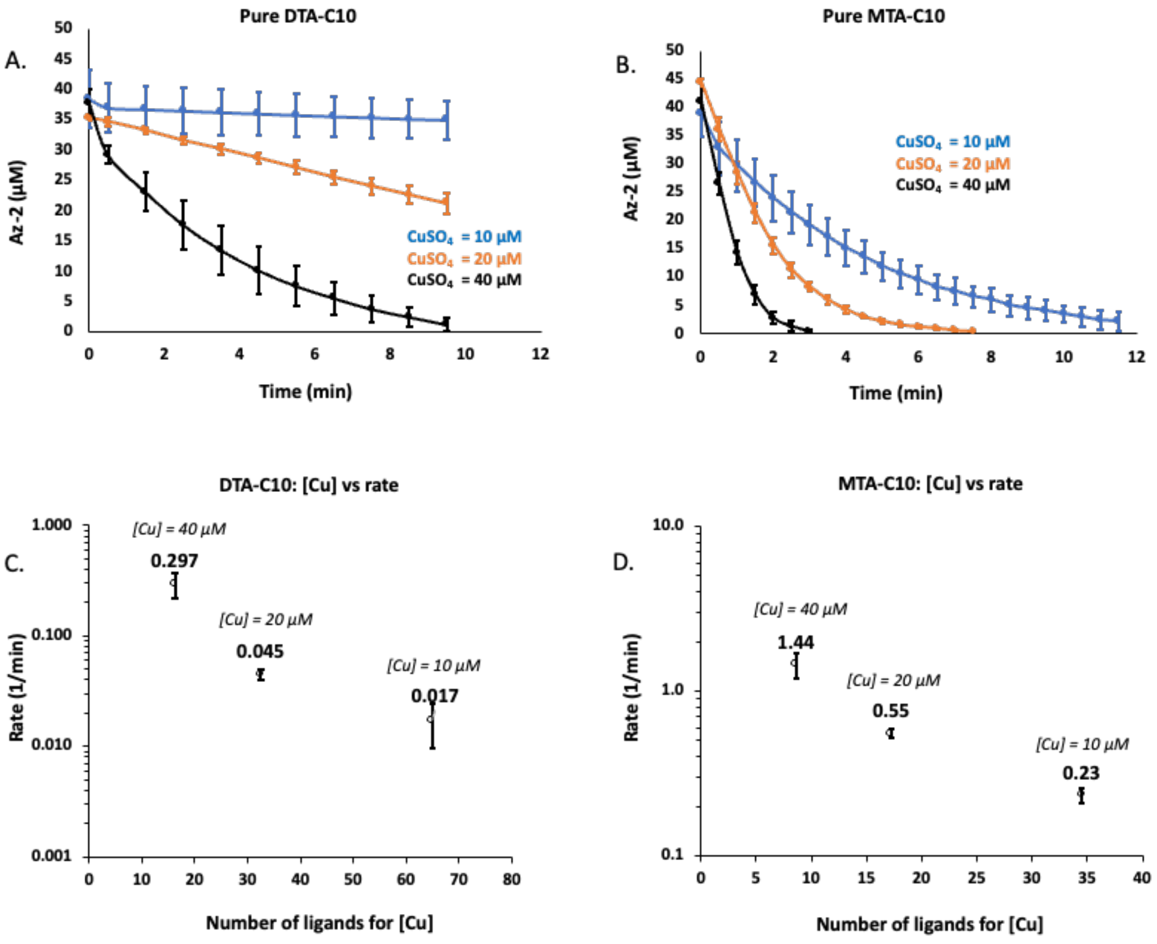

***Figure S43**: Azide-2 consumption rate with pure DTA-C10, and MTA-C10 micelles loaded with different amounts of [Cu]. Amphiphile = 2 mg/mL, [Alkyne-2] = 40 ± 5 µM, [Azide-2] = 40 ± 5 µM, [NaASC] = 2 mM **A.** Pure DTA-C10, **B.** Pure MTA-C10. **C** and **D** show the initial reaction rates obtained with different [Cu] for pure DTA-C10 and MTA-C10 respectively.*

Cu concentration in micelles

General procedure:

Micelles made with P123 amphiphiles and mixed micellar formulations (P123 with DTA-C10, MTA-C10, and TBTA, [amphiphile] = 2mg/mL) were loaded with $CuSO_4$ (10 µM final concentration, [Ligand] to [Cu] = 2 :1), vortexed briefly and the Cu content in the solution was analyzed with Inductively coupled plasma mass spectrometry (ICPS-MS).

Sample preparation for ICP-MS

0.2 to 0.5 mL of the sample solutions were mixed with 1 mL of concentrated nitric acid (65 % w/v) and 0.2 mL of concentrated hydrogen peroxide (30 % w/v) and hated at 95°C for 60 min. The solutions were diluted to the final volume of 10 mL with distilled water (MilliQ). Measurements were done on 7800 ICP-MS (Agilent), operated in helium mode with "general" plasma conditions according to the manufacturer's instructions. The signal of 63 copper isotope was measured with 3 replicates per measurement.

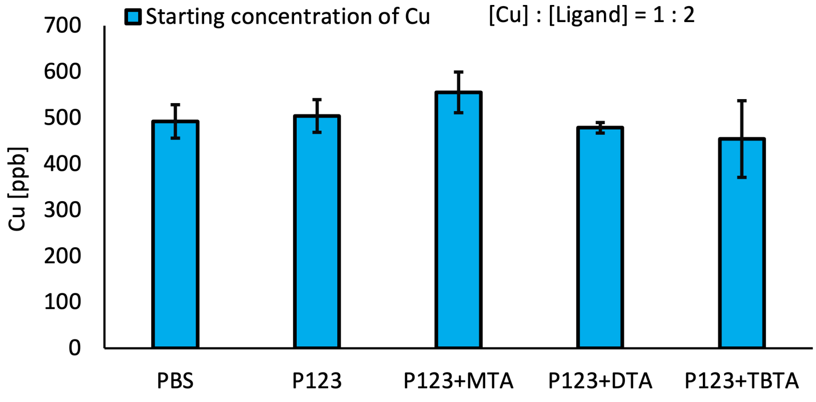

Figure S44: *Cu concentrations measured with ICP-MS.*

References:

(1) Schmitz, J.; Li, T.; Bartz, U.; Gütschow, M. Cathepsin B Inhibitors: Combining Dipeptide Nitriles with an Occluding Loop Recognition Element by Click Chemistry. *ACS Med. Chem. Lett.* **2016**, *7* (3), 211–216. https://doi.org/10.1021/acsmedchemlett.5b00474.

(2) Tevet, S.; Wagle, S. S.; Slor, G.; Amir, R. J. Tuning the Reactivity of Micellar Nanoreactors by Precise Adjustments of the Amphiphile and Substrate Hydrophobicity. *Macromolecules* **2021**, *54* (24), 11419–11426. https://doi.org/10.1021/acs.macromol.1c01755.

(3) Nozal, V.; García-Rubia, A.; Cuevas, E. P.; Pérez, C.; Tosat-Bitrián, C.; Bartolomé, F.; Carro, E.; Ramírez, D.; Palomo, V.; Martínez, A. From Kinase Inhibitors to Multitarget Ligands as Powerful Drug Leads for Alzheimer's Disease Using Protein-Templated Synthesis. *Angew. Chemie Int. Ed.* **2021**, *60* (35), 19344–19354. https://doi.org/https://doi.org/10.1002/anie.202106295.

(4) Mancuso, L.; Jürjens, G.; Hermane, J.; Harmrolfs, K.; Eichner, S.; Fohrer, J.; Collisi, W.; Sasse, F.; Kirschning, A. Bioreduction of Aryl Azides during Mutasynthesis of New Ansamitocins. *Org. Lett.* **2013**, *15* (17), 4442–4445. https://doi.org/10.1021/ol401989e.

(5) Jølck, R. I.; Sun, H.; Berg, R. H.; Andresen, T. L. Catalyst-Free Conjugation and In Situ Quantification of Nanoparticle Ligand Surface Density Using Fluorogenic Cu-Free Click Chemistry. *Chem. – A Eur. J.* **2011**, *17* (12), 3326–3331. https://doi.org/https://doi.org/10.1002/chem.201003131.
